# Root Proteomic Analysis of Two Grapevine Rootstock Genotypes Showing Different Susceptibility to Salt Stress

**DOI:** 10.3390/ijms21031076

**Published:** 2020-02-06

**Authors:** Bhakti Prinsi, Osvaldo Failla, Attilio Scienza, Luca Espen

**Affiliations:** Department of Agricultural and Environmental Sciences—Production, Landscape, Agroenergy (DiSAA), Università degli Studi di Milano, Via Celoria 2, 20133 Milano, Italy; bhakti.prinsi@unimi.it (B.P.); osvaldo.failla@unimi.it (O.F.);

**Keywords:** NaCl, *Vitis*, LC-ESI-MS/MS, carbon and energy metabolism, oxidative stress

## Abstract

Salinity represents a very limiting factor that affects the fertility of agricultural soils. Although grapevine is moderately susceptible to salinity, both natural causes and agricultural practices could worsen the impact of this abiotic stress. A promising possibility to reduce this problem in vineyards is the use of appropriate graft combinations. The responses of grapevine rootstocks to this abiotic stress at the root level still remain poorly investigated. In order to obtain further information on the multifaceted responses induced by salt stress at the biochemical level, in the present work we analyzed the changes that occurred under control and salt conditions in the root proteomes of two grapevine rootstock genotypes, M4 and 101.14. Moreover, we compared the results considering that M4 and 101.14 were previously described to have lower and higher susceptibility to salt stress, respectively. This study highlighted the greater capability of M4 to maintain and adapt energy metabolism (i.e., synthesis of ATP and NAD(P)H) and to sustain the activation of salt-protective mechanisms (i.e., Na sequestration into the vacuole and synthesis of osmoprotectant compounds). Comparitively, in 101.14 the energy metabolism was deeply affected and there was an evident induction of the enzymatic antioxidant system that occurred, pointing to a metabolic scenario typical of a suffering tissue. Overall, this study describes for the first time in grapevine roots some of the more crucial events that characterize positive (M4) or negative (101.14) responses evoked by salt stress conditions.

## 1. Introduction

It is estimated that about one fifth of the world’s cultivated soils is negatively affected by salinity. In addition to natural causes, land clearing and irrigation are among the main causes that are increasing this phenomenon [1]. Soil is classified as saline when it presents an electrical conductivity of more than 4 dS/m, corresponding to about 40 mM NaCl, a concentration that affects the productivity of most crops [2]. 

Salt stress is composed of osmotic and ionic components that characterize the two phases of the stress [2]. The first phase is mainly due to the increase of the osmotic pressure component of the soil solution and, likewise in drought conditions, determines a reduction of water availability, while the second phase depends on toxic effects consequent to the onset in plant cells of high concentrations of Na^+^ and/or Cl^−^. In these different phases, the transport activities at the membrane level play a crucial role, being involved in root ion uptake, cellular compartmentation (i.e., transport into the vacuole) and movement to the shoot, that mainly depends on transport from the symplast to the xylem apoplast [1,2,3]. According to an alteration in water balance, plants reduce transpiration, thus affecting many biological processes, like growth, photosynthesis and ion movement among tissues [3]. Moreover, the increases in Na^+^ and/or Cl^−^ dramatically affect the homeostasis of other mineral nutrients and metabolic functionality, as well as inducing the formation of reactive oxygen species (ROS) [1,2,3,4]. In this context, stress sensing and signaling components also play a very important role in the plant responses to salinity [5].

Although in many cases the toxic action of NaCl is linked to the accumulation of exceedingly high concentrations of Na^+^, in woody perennial species, like grapevine, the effects of this abiotic stress are mainly associated with an accumulation of Cl^−^ in the leaves [6]. A promising possibility to reduce the impact of increasing soil salt concentrations in vineyards is the use of appropriate graft combinations that exploit the genetic characteristics of the rootstocks (interspecific hybrids of different *Vitis* species, such as *V. berlandieri*, *V. riparia* and *V. rupestris*) concerning the capability to exclude the salt present in the soil and/or to reduce salt translocation to the shoot [7,8,9,10,11,12]. In this view, many studies focalized their attention on the transport activities at the root level as well as on the physiological, biochemical and molecular responses occurring in the shoot organs (i.e., leaves and fruits) in different grapevine graft combinations exposed to salt stress. However, the responses at the root level still remain poorly investigated [7,10,13,14,15,16].

In previous work, we compared the responses to increasing salt concentrations in soil solution of two rootstocks, i.e., M4 [(*V. vinifera* x *V. berlandieri*) x *V. berlandieri* cv. Resseguier no. 1] and 101.14 (*V. riparia* x *V. rupestris*) [17]; the study, conducted by applying gradual salt stress and following the responses for 21 days in order to analyze mainly the toxicity of the ion component [18], highlighted a lower and a greater susceptibility to salt stress of M4 and 101.14, respectively [17]. 

In order to obtain further information on the multifaceted responses at the biochemical levels that occur under salt stress in the roots (i.e., rootstock), in the present work we adopted the same experimental design to compare root proteomes of M4 and 101.14 genotypes in control and salt stress conditions, at the final time previously defined (i.e., 21 days of exposure to NaCl). 

## 2. Results and Discussion

As previously reported [17], the gradual exposure to NaCl (i.e., addition of 5 mmol NaCl daily for 21 days) induced in both the 101.14 and M4 rootstock genotypes a progressive reduction in stomatal conductance, photosynthetic activity and shoot growth, together with a decrease in the leaf water potential and a concomitant increase in osmolytes. Nevertheless, this previous study revealed that the negative effects induced by salt conditions were of a lesser extent in M4 than in 101.14, as highlighted by the lower inhibition of the photosynthetic performance and the higher accumulation of osmolytes. Moreover, M4 showed a greater ability to counteract the toxic action of Na^+^ in the leaves maintaining an adequate level of K^+^. Finally, a greater capability of M4 to preserve integrity and therefore functionality of the roots was observed [17]. Starting from this information and using the same experimental design, we focalized the present study on the longest duration of salt exposure.

First of all, the comparison of the morphology of the whole root organs highlighted that the salt exposure reduced the root volume and the number of young roots in 101.14, whilst these inhibitory effects appeared less evident in M4 (Figure S1, Supplementary Materials 1), confirming different responses in the two genotypes. To gain new knowledge at a biochemical level, we analyzed the accumulation of Na^+^ and Cl^−^ in the roots as well as the changes occurring in the root proteomes. 

### 2.1. Accumulation of Na^+^ and Cl^−^ in the Roots

**Figure 1:**
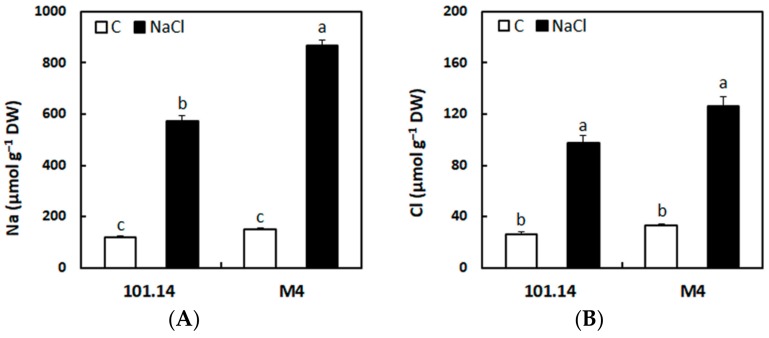
Levels of Na^+^ (**A**) and Cl^−^ (**B**) in roots of M4 and 101.14 grapevine rootstocks grown for 21 days in control (□) or salt stress (■) conditions. The values are the means ± Standard Error (SE) of three biological replicates (*n* = 3). The statistical significance was assessed by analysis of variance (ANOVA) test (*p* < 0.05, Tukey post hoc method).

The concentrations of Na^+^ and Cl^−^ significantly increased in both genotypes treated with NaCl for 21 days (Figure 1). The accumulation of Na^+^ was higher than that of Cl^−^, suggesting that the toxic effect occurring at this time could be ascribed mainly to the accumulation of this cation. Despite the greater ability to respond to salt stress [17], the levels of Na^+^ were higher in M4 than in 101.14, supporting the idea of a possible difference between the two genotypes in the capability to compartmentalize Na^+^ in the vacuole. 

The sequestration of Na^+^ into the vacuole represents an important strategy in the salt stress tolerance mechanism, because it participates in the maintenance of an adequate cytoplasmic K^+^/Na^+^ ratio [19]. Interferences of Na^+^ with K^+^ in the cytoplasm, in fact, can deeply affect the overall metabolic processes [2]. The tonoplast-localized Na^+^/H^+^ exchanger 1 (NHX1) plays a pivotal role in the vacuolar sequestration of Na^+^ [5,19]. Its activity can be energized by the vacuolar H^+^-ATPase (V-ATPase) and/or the H^+^-PPase (V-PPase) [5,20,21]. 

In order to investigate the possible differences in the vacuolar Na^+^ compartmentalization capability between the two genotypes, we conducted Western blot (WB) analyses to evaluate the protein abundance of NHX1, V-PPase and V-ATPase. For this last protein, the analysis was performed with an antibody produced against a conserved peptide of subunit E (see Materials and Methods for details). The transcription of this subunit is induced in salt stress conditions [22,23]. This result was confirmed at the protein level together with evidence sustaining a possible role of subunit E in the modulation of V-ATPase activity [23]. 

**Figure 2:**
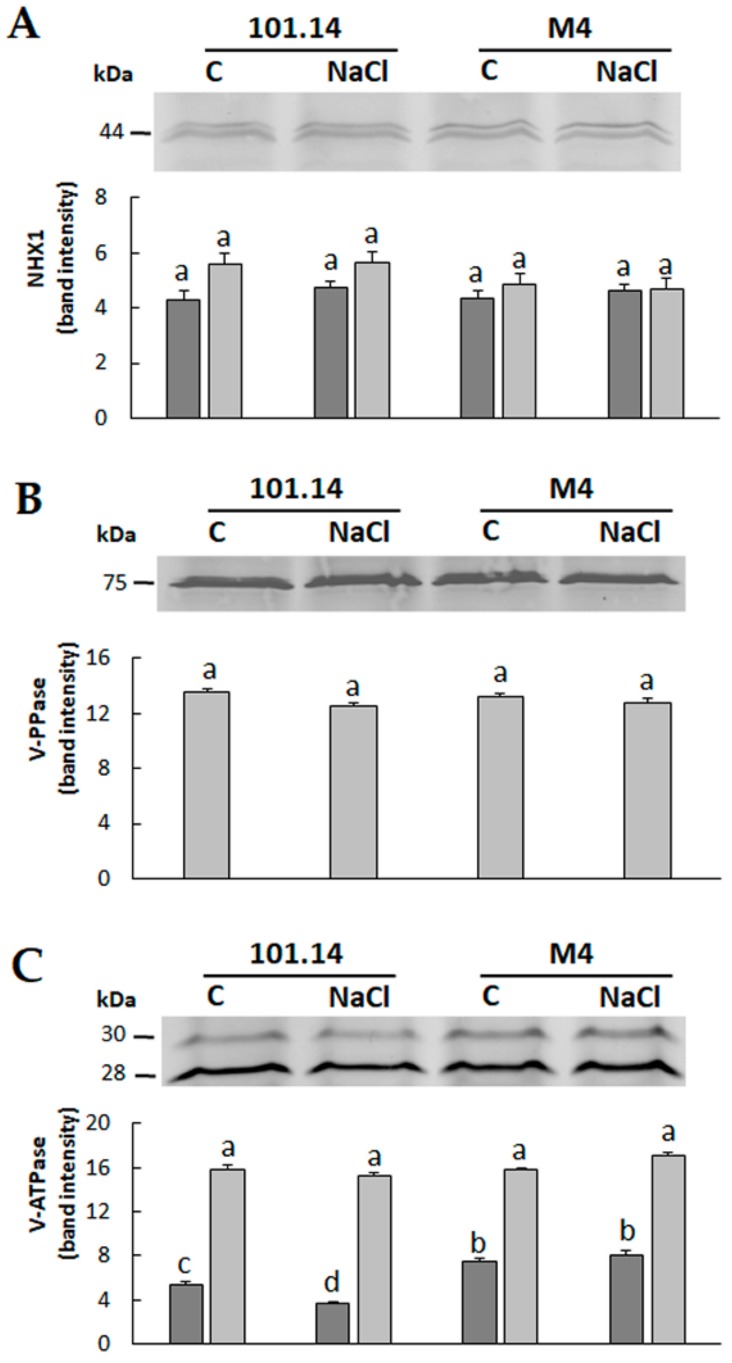
Western blot (WB) analyses of the NHX1 (**A**), V-PPase (**B**) and subunit E of V-ATPase (**C**) extracted from roots of 101.14 and M4 grapevine rootstocks grown for 21 days in control (C) or salt stress (NaCl) conditions. When two bands were detected, dark-grey bars refer to the band with higher MW, while light-grey bars to the band with lower MW. The intensity of bands described by the histograms was quantified by densitometric analysis with ImageJ. The values are the means ± Standard Error (SE) of three independent WB analyses (*n* = 3). The statistical significance was assessed by analysis of variance (ANOVA) test (*p* < 0.05, Tukey post hoc method).

In both genotypes, WB analyses did not reveal significant changes in protein abundance of the NHX1 and V-PPase (Figure 2A,B), whilst some differences occurred in the protein quantity of V-ATPase. Two distinct bands related to the subunit E of V-ATPase were visualized. This result was consistent with the presence of two protein isoforms, accordingly to the known sequences of *Vitis vinifera* deposited in the NCBI database. While the density of the band with a deduced molecular weight (MW) of 28 kDa did not show significant differences in the two genotypes, the band of 30 kDa decreased under the salt condition in 101.14 and remained unchanged in M4. In this genotype the abundances of this isoform were significantly higher than in 101.14 under both the control and the salt conditions (Figure 2C).

Taken together, these results highlighted that 101.14 and M4 had a similar capacity to transport Na^+^ into the vacuole in the control condition and that this activity was not affected by the salt treatment. Differently, the two genotypes could have a different capability of sustaining the proton gradient necessary to drive the sequestration of Na^+^ into the vacuole. In other words, the comparison between the two genotypes supports the idea that 101.14 could have a constitutively lower capability than M4 to pump H^+^ into the vacuole, that is further reduced under salt stress conditions. This aspect could be related to the previous observation that M4 showed a greater capability to cope with an adverse condition represented by salt stress [17]. In this view, the greater amounts of Na^+^ absorbed from the soil by M4 may be transported more efficiently into the vacuolar compartment (Figure 1A and Figure 2C).

Further studies may clarify the possible role of the 30 kDa isoform, that specifically responds to NaCl, in the modulation of V-ATPase activity [22,23].

### 2.2. Proteomic Analyses

The proteomic study was performed using the GeLC-MS/MS (gel liquid chromatography- tandem mass spectrometry) approach [24]. In detail, we combined the protein extraction method previously optimized for the root proteomes of grapevine plants grown in the soil [25] with an analytical streamlined procedure recently proposed [26], based on partial 1D SDS-PAGE purification and in gel-digestion procedure. The technical parameters related to protein identification and quantitation were very similar for the two genotypes and highlighted the good reliability of the adopted protocol (Table 1). Further information about the results is reported in Tables S1 and S2 (Supplementary Materials 1).

Proteomic analysis allowed the identification and quantification of a total of 280 and 271 proteins for the 101.14 and M4 genotypes, respectively. Among these, 31% and 34% changed in abundance under salt stress conditions in 101.14 and M4 genotypes, respectively. In 101.14, 39 proteins increased/appeared, and 48 proteins decreased/disappeared, while in M4, 40 proteins increased/appeared and 50 proteins decreased/disappeared (Tables S1 and S2). The proteins were classified from the functional point of view according to the bin hierarchical tree developed by MapMan ontology [27].

#### 2.2.1. Functional Distribution of the Identified Proteins

The functional distribution of the identified proteins is summarized in Figure S2 (Supplementary Materials 1). In the controls, the functional distribution was very similar in the two genotypes (Figure S2A,B). In this experimental condition the most represented categories were carbon and energy metabolism, protein and miscellaneous enzyme family. Salt exposure induced evident changes in all the functional categories, some of which were different in the two genotypes. Among these, carbon and energy metabolism, protein and lipid metabolism and miscellaneous enzyme families showed the greatest changes (Figure S2C,F). This result was quite similar to those found in studies concerning root proteomes of plants subjected to salt stress, even if a few differences were apparent, perhaps attributable to the experimental conditions or to peculiar responses of different species [28,29]. Among the functional classes with the higher number of proteins that appeared/increased under salt stress there were carbon and energy metabolism, the miscellaneous enzyme families and redox categories for 101.14 and carbon and energy metabolism, protein, and cell/signaling/ development categories for M4 (Figure S2C,D). Finally, in both genotypes an evident decrease/disappearance of proteins belonging to the categories of carbon and energy metabolism, lipid metabolism, and miscellaneous enzyme family was observed (Figure S2E,F). Taken together, these results showed that in optimal growth conditions the same activities were operating in roots of 101.14 and M4, whilst the addition of NaCl induced deep changes in the metabolism, some of which were different in the two genotypes.

#### 2.2.2. Metabolic Pathways Affected by Salt Stress

**Figure 3:**
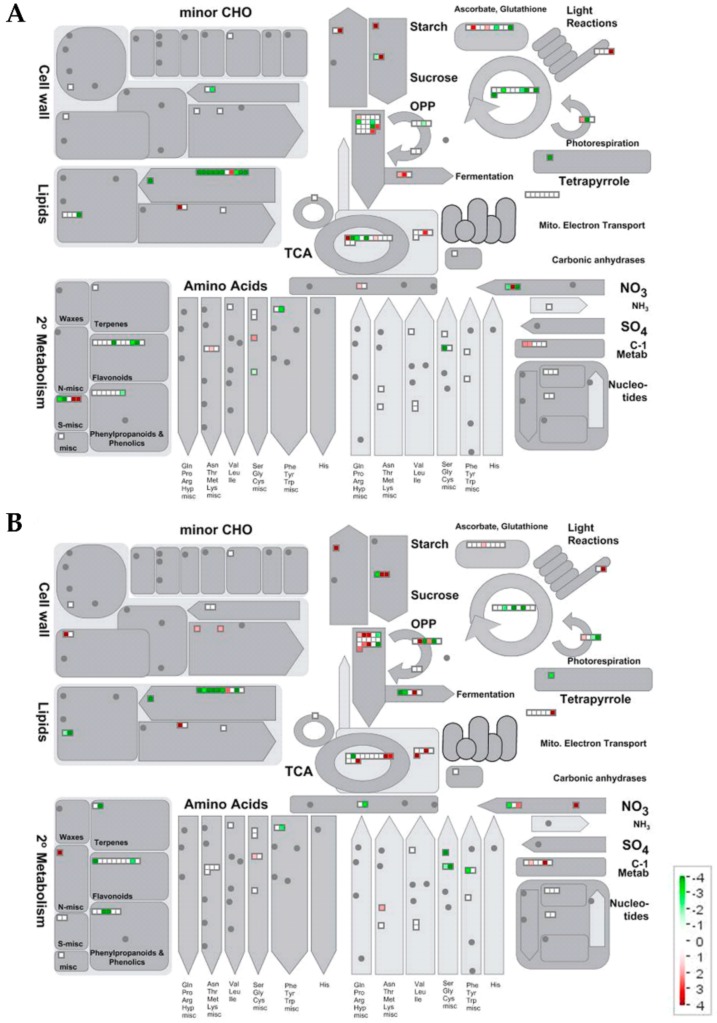
MapMan metabolism overview maps of the changes induced by salt stress condition. Changes in protein abundances (salt stress versus control) occurring in 101.14 (**A**) and M4 (**B**) grapevine rootstocks. Values are given in logarithmically scaled (base 1.2) signal intensities: red, increase; white, no change; green, decrease (see color scale).

**Figure 4:**
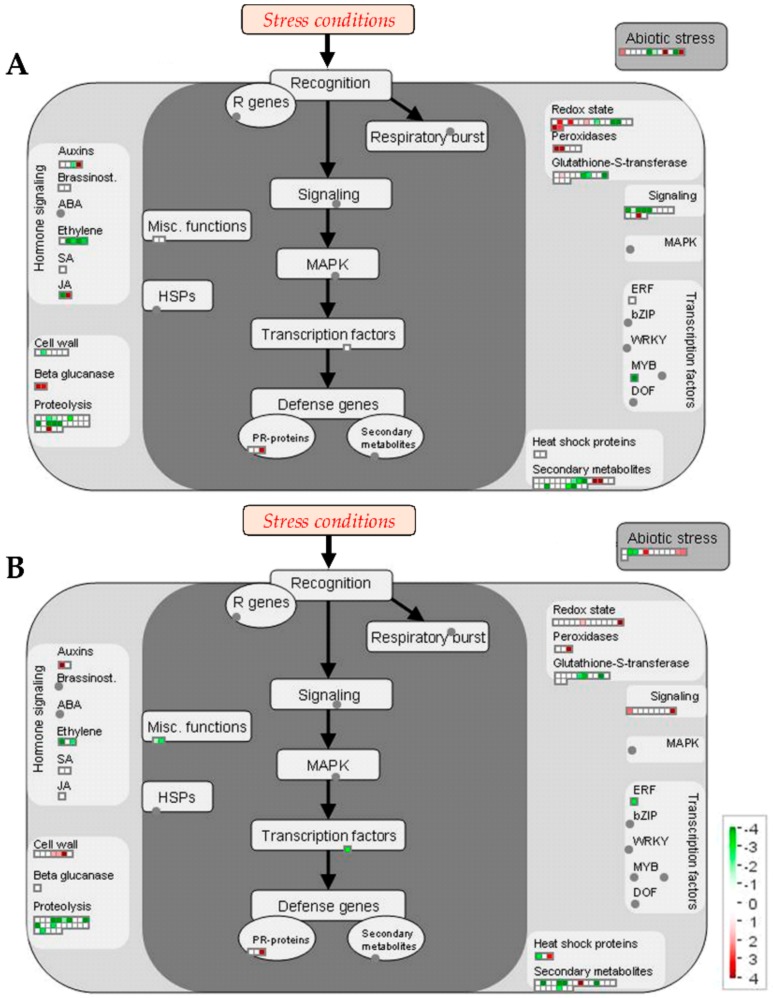
Changes in the MapMan overview related to stress pathways induced by salt stress. Changes in protein abundances (salt stress versus control) occurring in 101.14 (**A**) and M4 (**B**) grapevine rootstocks. Values are given in logarithmically scaled (base 1.2) signal intensities: red, increase; white, no change; green, decrease (see color scale).

Datasets containing all the identified proteins were displayed in a MapMan metabolism overview map (Figure 3) and in a MapMan map summarizing pathways known to be involved in stress responses (Figure 4). Table 2 and Table 3 show the proteins that significantly changed under salt stress in 101.14 and M4, respectively. According to a different capability to respond to salt stress, the analysis of the proteomic results highlighted many differences between the two genotypes.

##### Proteomic Changes Involved in Carbon and Energy Metabolism

The proteomic results revealed that many pathways involved in carbon and energy metabolism, like glycolysis, TCA cycle, ATP synthesis and oxidative pentose phosphate pathway (OPP), were affected by salt treatment in both genotypes, but in very different ways (Figure 3, Table 2 and Table 3).

In 101.14 a few enzymes of glycolysis and TCA cycle, such as fructose-bisphosphate aldolase (#24), glyceraldehyde-3-phosphate dehydrogenase (#61), and the dihydrolipoamide acetyltransferase component of pyruvate dehydrogenase complex (#120) were negatively affected by salt exposure, whilst pyruvate kinase (#58) and the E1 component subunit β of mitochondrial pyruvate dehydrogenase (#185) increased under stress conditions (Table 2). 

In M4, a greater number of enzymes involved in energy metabolism changed in abundance under the salt stress condition (Table 3). Glyceraldehyde-3-phosphate dehydrogenase (#42), fructose-bisphosphate aldolase (#45), and fructokinase (#13) decreased, whilst others, like cytoplasmic phosphoglucomutase (#64), pyruvate kinase (#97), and three subunits of mitochondrial succinate dehydrogenase (#227, #130 and #172), increased. Only in M4, an increase of the subunit O of mitochondrial ATP synthase took place (#133). Moreover, in this genotype, the α-subunit and β-subunit of pyrophosphate-fructose 6-phosphate 1-phosphotransferase increased and decreased, respectively (#91 and #100). This latter result could be due to a change in the possible forms described for this enzyme, composed by β-doublet β-single and α + β subunits, respectively [30,31]. Further work is requested to verify if the different ratio between the two subunits observed in the two experimental conditions could be a specific response to modulate the activity of this enzyme also in non-photosynthetic tissues. 

Taken together, the proteomic analyses highlighted that salt stress deeply influenced the main pathways involved in the biosynthesis of ATP, and this occurred in a manner similar to that described in roots of other species [28,29,30,31,32]. At the same time, the comparison between the two genotypes highlighted the greater capability of M4 to sustain the request of metabolic energy necessary to counteract the toxic effects of Na^+^ and/or Cl^−^, a crucial point in coping with a high salt concentration [33]. Although a few glycolytic enzymes diminished in a similar way in both genotypes, a few enzymes crucial in the TCA cycle and ATP synthesis increased only in M4. In this context, we also observed that only in 101.14 exposed to NaCl the abundance of two enzymes involved in the anaerobic metabolism for ATP production, i.e., pyruvate decarboxylase (#86) and alcohol dehydrogenase (#64), increased. At the same time, under salt stress, an evident decrease of pyruvate decarboxylase 1 occurred in M4 (#265). The results appear to show that, in 101.14, as reported for other species [34], salt stress can induce the activation of the fermentation pathway as a response to low ATP levels.

The reduction in the energetic metabolism may also depend on factors other than those referable to direct toxic effects of NaCl in root cells. In this view, an important aspect to consider is that the sugar availability in the root could decrease, since photosynthesis is affected by salt stress. Previously, in the same experimental conditions, a reduction of the net CO_2_ assimilation by 80% in 101.14 and by 35% in M4 was measured [17]. In this context, it is interesting to stress the dramatic increase in abundance of sucrose synthase in 101.14 (#184, + 150 folds) and its much lower increase in M4 (#89 and #152, + 11 and + 3-folds, respectively). This result fits well with a greater and lesser necessity of the two genotypes (101.14 and M4, respectively) to strengthen the import of photoassimilates in roots, in which sucrose synthase plays a pivotal role [35]. It could be observed that the toxic effects occurring in the leaf tissue evoke in roots metabolic responses that are apt at increasing sink strength. In this stress condition, the carbon skeletons imported from the phloem would be mainly used to sustain energy metabolism, rather than to synthesize starch, as suggested by the decrease in abundance of a few plastidial enzymes involved in carbohydrate metabolism. According to this observation, the effect was more evident in 101.14 than in M4 (Figure 3, Table 2 and Table 3). 

The multifaceted role of NADPH, involved in several biosynthetic pathways as well as in energy metabolism and in sustaining some antioxidant systems, is well known [36]. In non-photosynthetic tissues, the production of the reduced form NADPH depends on the activity of enzymes like glucose-6-phosphate dehydrogenase (G6PDH), 6-phosphogluconate dehydrogenase (6PGDH), and NADP-dependent malic enzyme (NADP-ME). In both genotypes, this latter enzyme is increased under salt stress (#7 and #18 in 101.14 and M4, respectively), suggesting that *Vitis* NADP-ME is also involved in the root responses to high NaCl concentration [37]. Only in M4 the salt stress induced an increase of G6PDH (#259) and 6PGDH (#31), suggesting that this genotype was able to also sustain the reduction of NADP^+^ by enhancing the operativeness of OPP (Figure 3, Table 2 and Table 3). 

At the same time, the increase in OPP could also be linked to the requirement of precursors for the synthesis of specific metabolite(s), which might contribute to counteracting the cellular effects of salt stress. Genes codifying G6PDH are classified among those that respond early to saline stress [38], being strictly involved in the response to osmotic stress [39]. In this context, it was shown that the salt stress responses could be linked to the expression of specific isoforms [40]. Under salt stress, an evident increase in the accumulation of a betaine aldehyde dehydrogenase (#235), involved in the synthesis of glycine-betaine, occurred in M4. The osmoprotectant glycine-betaine, whose synthesis requires reducing power, plays a central role in improving salinity and drought tolerance [41]. The result obtained in our study reinforces the relationship between G6PDH and the synthesis of osmolytes and highlights a further aspect involved in the capability of M4 to respond to salt stress conditions. 

##### Proteomic Changes Involved in Lipid Metabolism

Lipid metabolism was deeply affected by salt stress (Figure 3, Table 2 and Table 3). In both genotypes, only a phospholipase D (PLD, #100 and #111 in 101.14 and M4, respectively) increased under the salt stress condition. This enzyme, hydrolyzing structural phospholipids, produces phosphatidic acid that plays a key role in the signaling cascades involved in the control of many physiological processes as well as in the responses to stress conditions like salinity [42,43]. Consistent with the literature, our proteomic analysis revealed that in roots of grapevine, PLDs were also involved in the perception of salt stress. In both genotypes, the same enzymes, such as biotin carboxylase 1, an enoyl-[acyl-carrier-protein] reductase [NADH], 3-hydroxyacyl-[acyl-carrier-protein] dehydratase FabZ, biotin carboxyl carrier protein of acetyl-CoA carboxylase 2, 3-oxoacyl-[acyl-carrier-protein] reductase 2, and dihydroceramide fatty acyl 2-hydroxylase FAH1 (#41, #70, #182, #211, #105, #173 and 65#, 60#, 168#, 203#, 88#, #125, in 101.14 and M4, respectively), known to be involved in the plastidic biosynthetic pathway of fatty acids [44,45] were affected by salt stress. Interestingly, this response seemed more marked in 101.14 (Table 2 and Table 3). Although further work is necessary to clarify this point, the observed evident reduction of lipid metabolism could be a consequence of a different use of carbon skeletons and/or cellular energy. Moreover, the capability to counteract the reduction in fatty acids biosynthesis induced by salinity could also represent in roots a crucial point in the determination of salt tolerance, considering that deficiencies in this pathway can determine premature cell death and morphological alterations [46].

##### Proteomic Changes Involved in N, Amino Acid and Protein Metabolism

Under salt stress a few enzymes involved in the amino acid metabolism were affected in both genotypes (Figure 3, Table 2 and Table 3). The effects of salt stress conditions on amino acid metabolism are well known. This could be a direct consequence of the toxic effects of salt (particularly Na^+^) on the energy metabolism and/or on carbon skeleton availability, could depend on changes in protein turnover (i.e., different ratio between protein biosynthesis and degradation), but also could be linked to the biosynthesis of specific amino acids with an osmoprotective/antioxidant role [41,47,48]. A previous study, conducted in our laboratory using the same salt stress conditions adopted in the present work, showed that the total amino acid contents increased in both genotypes, whilst the total protein contents were not affected [17]. 

Whilst some changes in amino acid metabolism were common in the two genotypes, others were observed only in one of them. In both genotypes, a down-accumulation of a bifunctional 3-dehydroquinate dehydratase/shikimate dehydrogenase (#191 and #155 in 101.14 and M4, respectively) and a glyoxylate/hydroxypyruvate reductase A HPR2 (#116 and #139 in 101.14 and M4, respectively) occurred under salt stress. These enzymes are involved in the synthesis of aromatic amino acids and in the metabolism of amino acids belonging to the glycine group, respectively [49,50]. At the same time, salt stress induced in both genotypes a decrease of the cytosolic form of glutamine synthetase (#36 and #26 in 101.14 and M4, respectively), that could be linked to a change in plant N recycling [51,52]. Only in M4, nitrite reductase 1 (#231) increased under salt stress, supporting the idea that M4 might have a greater capacity to sustain N assimilation under the salt stress condition adopted. At the same time, an up-accumulation of a serine hydroxymethyl-transferase (#27), that catalyzes the conversion of glycine to serine and is reported to play an important role in leaf tissue(s) in counteracting (a)biotic stresses [53], occurred in 101.14. 

Some peptidases like cysteine proteinase RD21A (#157 and #121 in 101.14 and M4, respectively), a carboxypeptidase (#195 and #185 in 101.14 and M4, respectively), procardosin-A (#56 and #63 in 101.14 and M4, respectively), and a peptidase_S10 domain-containing protein (#135 and #221 in 101.14 and M4, respectively) decreased in abundance under salt stress in both genotypes, consistent with an overall reduction in protein degradation. At the same time, only in 101.14 a 26S proteasome non-ATPase regulatory subunit 2 homolog (#149) increased and two chaperonins 60 subunit α 2 (#48 and #122) decreased. Differently, in M4 a proteasome subunit β type (#165) decreased, whilst two elongation factors (#30 and #105) and a 40S ribosomal protein SA (#80), required for the assembly and/or stabilization of the 40S ribosomal subunit, increased under salt stress. Taken together, these data highlight the greater capability of M4 than 101.14 to sustain activation of protein synthesis in response to salt stress.

In both genotypes, a regulatory subunit A β isoform of serine/threonine-protein phosphatase 2A (PP2A) was positively affected by the salt-stress condition (#266 and #260 in 101.14 and M4, respectively). PP2As are involved in reversible protein phosphorylation, a post-translational modification that plays a central role in a plethora of processes among which are environmental stress responses [54,55]. Further studies may clarify the specific role, if any, of this regulatory protein in the response to salt stress.

##### Proteomic Changes Involved in Secondary Metabolism, Stress and Redox Metabolisms

Salt stress negatively affected proteins classified in the secondary metabolism functional class (Figure 3, Table 2 and Table 3). Among the identified proteins a flavanone 3-hydroxylase (#93 and #101 in 101.14 and M4, respectively) was present, that decreased in both genotypes, suggesting a reduction in the synthesis of flavonoids. The effect of salt stress on the phenolic metabolism was more evident in 101.14, where cinnamyl alcohol dehydrogenase 8 (#161) and chalcone synthase (#155) also decreased in abundance. Only in 101.14, salt stress induced the appearance of a bifunctional nitrilase/nitrile hydratase NIT4B-like (#261). This enzyme, that is involved in the metabolism of cyanide (i.e., β-CAS pathway), removes β-cyanoalanine, producing NH_4_^+^ and aspartate [56]. The relationship between cyanide metabolism and ethylene synthesis under stress conditions is described [57,58,59]. Although we did not observe any change in the enzymes involved in ethylene metabolism, it was realistic to hypothesize that the salt stress condition induced in 101.14 the synthesis of this hormone, consistent with the suffering state detectable by morphological analysis of the roots of 101.14 (Figure S1, Supplementary Materials 1).

In both genotypes, a chitinase class I basic (#51 and #54 in 101.14 and M4, respectively) was up-accumulated under salt stress conditions (Table 2 and Table 3). A similar result was previously found in roots of the 101.14 and M4 genotypes in response to drought stress [25], thus reinforcing the idea that enhanced synthesis of this enzyme could be a response useful to counteracting the risk of infection in stress-weakened plants [60,61]. In 101.14, two germin-like proteins (#201, #214) showed an evident increase in abundance, while another one slightly diminished (#128). The germin subfamily is a heterogeneous class of proteins described to be involved in the defense response to different stress conditions, such as salt stress [62,63]. Salt stress induced in M4 an increase in MLP-like protein 34 (#110). Although further work is necessary to define the specific role, it could be stressed that the increase of MLP-like proteins have been related to a greater tolerance to stress conditions [64].

Many stresses, including salinity, are characterized by an evident increase in reactive oxygen species (ROS), that attack cell membranes and macromolecules finally affecting cell/tissue structures and metabolism functionality [65,66]. Although ROS play a key role in the responses to abiotic stress, excess levels of these compounds induce the typical activation of enzymatic and non-enzymatic systems to remove them [67]. In our experimental conditions, both 101.14 and M4 genotypes showed an increase in antioxidant enzymes under salt stress, even if the entity of the response was much more evident in 101.14 than in M4 (Table 2 and Table 3, Figure 3 and Figure 4). Whilst in M4 only a catalase (#16) and a peroxidase 53 (#263) increased, in 101.14, several antioxidant enzymes like a superoxide dismutase [Cu-Zn] (#222), a monodehydroascorbate reductase 5, mitochondrial isoform X1 (#59), a protein disulfide-isomerase (#29), a catalase (#12), a monodehydroascorbate reductase (#44) and two peroxidases (#153 and #94), were up-accumulated. The greater activation of these enzymes that occurred in 101.14 suggests that this genotype must respond to a more severe oxidative stress than that occurring in M4. In other words, the lesser capability to counteract the toxic effects induced by the presence of salt concentration, suggested by several results of the present study (see above), may lead to an increase in ROS. In this view, a decrease of a GDP-mannose 3,5-epimerase (#45), involved in ascorbate biosynthesis [68,69], did occur in 101.14, suggesting a further difficulty of this genotype in counteracting oxidative stress. Our proteomic analyses revealed the presence of the same glutathione transferase (GST) isoforms in both genotypes, all decreased under salt stress conditions (#30, #139 and #134 in 101.14, #36, #86 and #107 in M4). GSTs are a large group of multifunctional enzymes that show different responses to salt stress [70] and that in some cases resulted in the improvement of salt tolerance [71]. Further work is necessary to define the biochemical/physiological role(s) of the GSTs whose levels have been observed to change in the present study.

#### 2.2.3. Final Considerations

**Figure 5:**
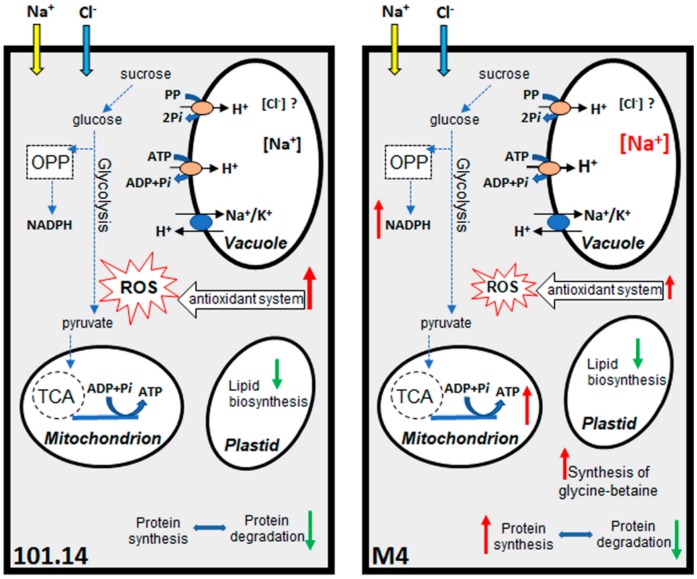
Schematic overview summarizing the main differences between 101.14 and M4 genotypes in biochemical processes highlighted by the proteomic analysis. Red arrows indicate an increase while green arrows indicate a decrease in the process.

This work provides new information about the responses to salt stress of the root organ of grapevine plants. The comparative proteomic analysis between two genotypes, which were previously shown to have lower (M4) or higher (101.14) susceptibility to high NaCl concentrations [17], allow for the identification of a few crucial traits that seem to play a central role in the biochemical responses and/or in relieving the salt effects. The main changes occurring under salt stress in the two genotypes are summarized in Figure 5. According to studies conducted in other plant species, the capability to sequestrate Na^+^ into the vacuole appears to play a key role in the salt response also in the roots of this woody plant. The capability to sustain the energy cost required by the salt-protective mechanisms is a very central point in the response [33,72]. In this context, the M4 genotype turned-out to better sustain the pathways involved in the synthesis of ATP and NADPH. The ability to maintain protein synthesis as well as to produce osmoprotectant compounds, such as glycine-betaine, are other traits found only in M4. On the contrary, in 101.14 a very critical situation emerged. In this more sensitive genotype, the energy metabolism was deeply affected. This deficiency could depend on several factors, like a lesser capability to sequester Na^+^ into the vacuole, but also the higher difficulty to import sugars from the shoot into the root. In the salt stress condition, an evident induction of the enzymatic antioxidant system occurred, even if the simultaneous difficulty to adequately sustain the production of reducing power (i.e., the synthesis of NADPH and ascorbate) seemed to undermine the capability of 101.14 root tissues to operate against the salt stress condition.

Overall, this study provides new knowledge about biochemical responses occurring in grapevine rootstocks exposed to salt stress conditions. This information may be useful in future investigations needed to verify the performance of these genotypes in different graft combinations.

## 3. Materials and Methods 

### 3.1. Sample Material and Growth Conditions

The 101.14 Millardet et de Grasset (*V. riparia* x *V. rupestris*) and M4 [(*V. vinifera* x *V. berlandieri*) x *V. berlandieri* cv. Resseguier no. 1] grapevine rootstock genotypes were grown as previously described [17]. In detail, two-year-old plants were grown in 3-liter pots filled with sand–peat mixture (7:3 *v*/*v*). After a period of two weeks, to permit the acclimation of plants in the greenhouse conditions, an adequate number of uniform plants (i.e., similar height, number of sprouts and total leaf area) were selected for the experiment. The control plants were grown in a soil in which the field capacity was maintained at 80%. Salt stress was induced by adding 5 mmol of NaCl each day, to achieve a final NaCl concentration of ca. 120 mM, and maintaining the same soil field capacity of the control condition. After 21 days, the whole root system was sampled removing the soil by gentle shaking, rinsed twice with distilled water, blotted with paper towels, weighed and frozen in liquid N_2_. Samples were then grinded in liquid N_2_ to obtain a fine powder that was stored at –80 °C. Aliquots of the samples were used for the different analyses. For each experimental condition, three biological samples, each derived from six randomly plants, were obtained.

### 3.2. Chloride and Sodium Quantification

Powder samples were suspended in three volumes of extraction solution (0.2 mM HNO_3_), boiled for 15 min and centrifuged at 10,000× *g* for 10 min. After centrifugation, the supernatants were collected (SN1), pellets were resuspended in 3 mL of distilled water and centrifuged at 10,000× *g* for 10 min to obtain SN2. The two supernatants were pooled (SN1 + SN2) and distilled water was added to a final volume of 10 mL. The chloride content was then evaluated by QuantiChrom™ Chloride Assay Kit (BioAssay Systems, Hayward, CA, USA) following the manufacturer’s instructions. Sodium concentration was measured by ICP-MS as previously described [17].

### 3.3. Protein Extraction 

The total protein fraction was extracted from three biological replicates for each experimental condition as previously described [25,73] and dissolved in SDS-buffer (150 mM Tris-HCl, pH 6.8, 10% (*w*/*w*) glycerol, 2% (*w*/*w*) sodium dodecyl sulphate (SDS), 2% (*v*/*v*) 2-mercaptoethanol). After 5 min at 95 °C, samples were centrifuged at 10,000× *g* for 10 min and the supernatant was collected and stored at −80 °C. The protein concentration was determined by the 2-D Quant Kit (GE Healthcare Europe GmbH, Freiburg, Germany). 

### 3.4. Western Blot Analyses

Protein samples (15 μg) were diluted with an equal volume of SDS-buffer added with 0.01% (*w*/*v*) bromophenol blue, heated for 5 min at 90 °C, separated by SDS-PAGE using 10.0% (*w*/*v*) acrylamide [74] and then electrophoretically transferred onto a polyvinylidene difluoride (PVDF) filter using the Trans-Blot Turbo System (Bio-Rad Laboratories, Hercules, CA, USA) in the presence of a buffer containing 25 mM Tris, 192 mM glycine pH 8.3 and 20% (*v*/*v*) methanol. Filters were blocked for 1 h with TBS-T buffer (50 mM Tris–HCl (pH 7.6), 200 mM NaCl, and 0.1 % (*v*/*v*) Tween 20) supplemented with 5% (*w*/*v*) of albumin. After three washings of 5 min in TBS-T, filters were further blocked for 1 h with TBS-T supplemented with 5% (*w*/*v*) nonfat dried milk. After three washings (5 min each) in TBS-T, filters were incubated overnight at 4 °C with primary polyclonal antibodies raised against the E (i.e., ε) subunit of tonoplast H^+^-ATPase (1:2000 dilution, Agrisera, AS07 213), the Na^+^/H^+^ antiporter, sodium/hydrogen exchanger (1:1000 dilution, Agrisera, AS09 484) and the vacuolar H^+^-pyrophosphatase (1:2000 dilution, Agrisera, AS12 1849). After washing with TBS-T, the filters were incubated for an additional 2 h at room temperature with a secondary antibody (alkaline phosphatase-conjugated anti-rabbit IgG, Sigma A3687). The blot was developed with nitroblue tetrazolium and 5-bromo-4-chloro-3-indolyl phosphate (FAST-BCIP/NBT, Sigma, B5655). Three technical replicates were performed, and quantification of the bands was conducted through densitometric analysis by using the software ImageJ (https://imagej.net/).

### 3.5. Gel Electrophoresis, In-Gel Digestion and Mass Spectrometry Analysis

Gel electrophoresis, in gel-digestion and mass spectrometry analyses were performed as previously described [26]. Briefly, 15 µg of proteins were purified by partial 1D SDS-PAGE on 16% (*w*/*v*) polyacrylamide gel, accordingly to Leammli procedure [74] at 60 mV for 30 min. After Coomassie Brilliant Blue staining, gel bands were subjected to tryptic digestion [75], with the refinements described in [26].

All mass spectrometry experiments were conducted with an Agilent 6520 Q-TOF mass spectrometer equipped with an HPLC Chip Cube (Agilent Technologies, Cernusco sul Naviglio, Italy), as previously described [26]. In detail, chromatography was performed in a Polaris-HR-Chip-3C18 (Agilent Technologies), consisting of a 360-nL trap column and a 75 µm × 150-mm analytical column (Polaris C18-A, 180 Å, 3 µm), applying a 100-min non-linear gradient of acetonitrile from 5% to 50% (*v*/*v*) at 0.4 µL min^−1^. Acquisition and analysis of the MS/MS spectra were performed with the following adaptations. The search was conducted against the *Vitis* (ID 3603) protein database downloaded from UniProtKB/Swiss-Prot (http://www.uniprot.org/) and concatenated with the reverse one. The threshold used for protein identification was peptide false discovery rate (FDR) ≤ 1% and number of unique peptides *per* protein ≥ 2. Peptide quantification was obtained as the spectrum intensity (SI) of the precursor (MH^+^). Protein quantification was obtained by summing the SI of all the identified peptides in the protein. Protein abundance was normalized as the percentage with respect to the abundance of all validated proteins in the sample [%(SI)]. Two technical replicates were performed for each biological sample (*n* = 3). Proteins showing a fold-change of at least 1.4 between the two conditions (salt stress versus control) and for which the change was significant according to the Student’s t-test (*p* < 0.05) were considered as significantly changed in abundance. The identified proteins were classified into metabolic functional classes according to the MapMan BIN ontology. The schematic metabolic pathways were obtained by MapMan software as previously described [25].

## Figures and Tables

**Table 1:** Evaluation of the comparative proteomic analyses in roots of 101.14 and M4 rootstock genotypes.

Parameter	101.14	M4
*n*. of peptides *per* genotype	15,105	15,131
Average of unique peptide *per* protein (±SE)	5.0 ± 0.2	5.2 ± 0.2
Average protein intensity	1.43 ± 0.039 (×10^6^)	1.72 ± 0.049 (×10^6^)
Dynamic range of protein intensity	4.2 × 10^3^–2.3 × 10^7^	7.7 × 10^3^–2.5 × 10^7^
Average protein score (Spectrum Mill)	78.4 ± 3.7	79.7 ± 3.9
Average amino acid coverage % (±SE)	17.4 ± 0.7	17.4 ± 0.7
*n*. of identified proteins (i.e. protein groups)	280	271
*n*. of differentially accumulated proteins (%)	87 (31%)	90 (34%)

**Table 2:** Proteins showing significant changes in responses to salt stress in the 101.14 genotype.

#	Accession	Name (f.c.)	ΔSS/C
*Carbon and energy metabolism (1-9, 25)*			
184	A5C6H7	Sucrose synthase (2)	150.46
185	F6I1P0	Pyruvate dehydrogenase E1 component subunit beta, mitochondrial (8)	3.49
146	F6H710	Galactokinase, putative (3)	2.17
64	F6I0F6	Alcohol dehydrogenase (5, 26)	1.78
7	A0A1Z2THL4	NADP-dependent malic enzyme (8)	1.75
58	C5DB68	Pyruvate kinase, cytosolic isozyme (4, 11)	1.70
175	F6GX20	4-hydroxy-4-methyl-2-oxoglutarate aldolase (25)	1.52
86	D7TJI9	Pyruvate decarboxylase 1 (5)	1.42
101	A5BEM8	Putative oxidoreductase GLYR1 (7)	−1.43
24	F6HFL6	Fructose-bisphosphate aldolase (4)	−1.59
120	F6HFN8	Dihydrolipoamide acetyltransferase component of pyruvate dehydrogenase complex (8, 11)	−1.74
61	D7T0U8	Glyceraldehyde-3-phosphate dehydrogenase (1)	−1.77
144	F6HI27	Pyruvate dehydrogenase E1 component subunit beta-2, chloroplastic (8, 1, 11)	−4.93
60	F6GTG3	Enolase 1, chloroplastic-like (4)	−6.75
168	D7SY46	Dihydrolipoyl dehydrogenase 2, chloroplastic (8, 11, 21)	−26.09
*Lipid metabolism (11)*			
100	F6HXC8	Phospholipase D (11, 1, 27)	3.90
41	F6H9P9	Biotin carboxylase 1, chloroplastic (11)	−2.57
70	F6HLJ7	Enoyl-[acyl-carrier-protein] reductase [NADH] 1, chloroplastic (11)	−2.13
182	D7STF0	3-hydroxyacyl-[acyl-carrier-protein] dehydratase FabZ (11)	−3.22
211	D7T4I1	Biotin carboxyl carrier protein of acetyl-CoA carboxylase 2, chloroplastic (11)	−4.35
105	D7TAP7	3-oxoacyl-[acyl-carrier-protein] reductase 2, chloroplastic (11, 26)	−12.24
173	A5C5V3	Dihydroceramide fatty acyl 2-hydroxylase FAH1 (11)	−32.25
188	D7TVI4	3-oxoacyl-[acyl-carrier-protein] synthase I, chloroplastic (11)	d.
*N and amino acid metabolism (12, 13)*			
27	F6GWF3	Serine hydroxymethyltransferase (13, 1)	1.49
36	P51119	Glutamine synthetase cytosolic isozyme 2 (12)	−1.54
191	A5ATW2	Bifunctional 3-dehydroquinate dehydratase/shikimate dehydrogenase (13)	−1.79
116	A5CAL1	Glyoxylate/hydroxypyruvate reductase A HPR2 (13, 1, 26)	−2.59
*Secondary metabolism (16)*			
256	F6HHQ2	Nitrile-specifier protein 5 (16)	New
261	F6I080	Bifunctional nitrilase/nitrile hydratase NIT4B-like (16, 26)	New
161	D7TRU0	Cinnamyl alcohol dehydrogenase 8 (16)	−1.50
215	D7U0Q6	Probable plastid-lipid-associated protein 1, chloroplastic (16, 17, 26)	−1.78
93	A0A0M5I8D0	Flavanone 3-hydroxylase (16)	−1.78
155	O22519	Chalcone synthase (16)	−14.75
250	S5FNE7	Protein SRG1 (16, 17)	d.
*Hormone metabolism (17)*			
85	D7TUK1	Perakine reductase isoform X1 (17, 26)	5.71
207	A3QRC1	Allene oxide cyclase 2, chloroplastic (17, 20)	−2.06
224	F6HX49	Gibberellin 20 oxidase 1 (17, 16, 26)	−2.39
*Stress (20)*			
201	Q0MYQ7	Germin-like protein 2 (20, 15, 31)	44.61
51	A3QRB7	Chitinase class I basic (20)	2.47
214	F6HZ19	Germin-like protein subfamily 1 member 17 (20, 12, 27, 34)	2.46
96	A5ASS2	Thaumatin (20)	1.60
128	A5BAY1	Germin-like protein 9-2 (20, 15)	−1.42
165	D7TKM8	Putative germin-like protein 2-1 (20, 12, 27, 30, 34)	−2.42
*Redox (21)*			
222	F6HTY5	Superoxide dismutase [Cu-Zn] (21)	2.11
59	A5C8L8	Monodehydroascorbate reductase 5, mitochondrial isoform X1 (21)	1.84
29	E0CR49	Protein disulfide-isomerase (21)	1.81
12	Q8S568	Catalase (21)	1.69
44	A5JPK7	Monodehydroascorbate reductase (21)	1.43
45	F6HDW4	GDP-mannose 3,5-epimerase (10, 21)	−1.61
*Miscellaneous enzyme families (26)*			
153	A7NY33	Peroxidase 4 (26, 16)	New
156	Q69D51	Beta-1,3-glucanase (26)	New
94	F6GWS4	Peroxidase (26)	4.88
22	Q9M563	Beta-1,3-glucanase (26)	1.90
90	A5AKD8	Peptidyl-prolyl cis-trans isomerase (26)	1.44
30	F6HR72	Glutathione S-transferase (26)	−1.66
139	F6GT84	Glutathione S-transferase U9 (26)	−1.97
104	D7TE48	Soluble epoxide hydrolase (26)	−2.41
134	A5AZ36	Glutathione S-transferase U25 (26)	−2.85
249	F6HL77	Tropinone reductase homolog At1g07440 (34, 2)	−29.52
190	D7T8G2	Purple acid phosphatase (26)	−38.33
170	F6HZD8	Short-chain dehydrogenase reductase 3b-like (26)	d.
*DNA/RNA (27,28)*			
121	A5B427	Cyclase (28)	1,41
223	A5AXT8	Pentatricopeptide repeat-containing protein At5g66520-like (27, 26)	d.
*Protein (29)*			
277	F6H2W4	Aspartate--tRNA ligase 2, cytoplasmic (29)	New
266	D7SIX7	Serine/threonine-prot. phosphatase 2A 65 kDa regulatory sub. A isoform (29)	5.60
149	E0CTI4	26S proteasome non-ATPase regulatory subunit 2 homolog (29)	2.92
56	F6H7H1	Procardosin-A (29)	−1.40
157	F6GZY7	Cysteine proteinase RD21A (29)	−1.54
135	A5BIH7	Peptidase_S10 domain-containing protein (29)	−1.82
195	F6H1D7	Carboxypeptidase (29)	−2.08
186	D7SHN2	Heme-binding protein 2-like (29, 19)	−2.63
48	F6GWA8	Chaperonin 60 subunit alpha 2, chloroplastic (29, 1)	−3.30
122	D7SLM9	Chaperonin 60 subunit beta 2, chloroplastic (29, 1)	−3.48
*Cell/signaling/development (30, 31, 33)*			
218	A5ARE0	Glutelin type-A 1-like (33, 28)	6.08
89	A5BXT5	Guanosine nucleotide diphosphate dissociation inhibitor (30)	2.05
131	D7T2N7	Late embryogenesis abundant protein Lea14-A (33)	1.52
73	A5AKB1	Plastid-lipid-associated protein 1, chloroplastic (31)	−1.85
202	D7T9L8	Coatomer subunit delta (31)	−2.21
*Transport (34)*			
246	F6H9B5	Glucose-6-phosphate/phosphate translocator 1, chloroplastic	4.78
133	F6HXK4	Plasma membrane ATPase (34)	2.32
210	F6HS56	Potassium channel beta, putative (34, 17)	−1.59
*Others (15, 18)*			
227	F6H2P8	Protein DJ-1 homolog B (18)	6.81
*Not assigned (35)*			
87	F6HHU9	Uncharacterized protein (35)	16.51
37	F6HUS6	Uncharacterized protein (35)	1.78
136	F6HJB9	Uncharacterized protein (35)	−2.30
238	F6H0J2	DPP6 N-terminal domain-like protein (35)	−2.53
219	A5B729	Uncharacterized protein	d.

Numbers reported in brackets refer to bin code (i.e., major functional categories). **#**: identification number. **f.c.**: bin code of functional categories. **ΔSS/C**: fold changes in salt-stressed plants compared to the control ones (up: %(SI)WS/%(SI)C, down: - %(SI)C/%(SI)WS). **new**: not present in the controls; **d.**: disappeared, not present in salt-stressed plants.

**Table 3:** Proteins showing significant changes in responses to salt stress in the M4 genotype.

#	Accession	Name (f.c.)	ΔSS/C
*Carbon and energy metabolism (1-9, 25)*			
89	A5C6H7	Sucrose synthase (2)	10.91
235	D7SHY3	Betaine aldehyde dehydrogenase 1, chloroplastic (5, 16)	8.62
64	F6HFF7	Phosphoglucomutase, cytoplasmic 1 (4)	3.46
227	A5BF93	Succinate–CoA ligase [ADP-forming] subunit beta, mitochondrial (8)	3.44
152	F6HGZ9	Sucrose synthase (2)	3.11
133	D7T300	ATP synthase subunit O, mitochondrial (9)	3.06
259	F6HHP3	Glucose-6-phosphate 1-dehydrogenase (7, 30)	3.01
130	F6H9T6	Succinate-semialdehyde dehydrogenase, mitochondrial (8)	2.45
18	A0A1Z2THL4	NADP-dependent malic enzyme (8)	2.24
187	F6I5I7	Methylenetetrahydrofolate reductase (25)	2.24
91	F6I6W5	Pyrophosphate–fructose 6-phosphate 1-phosphotransferase subunit alpha (4)	1.97
172	D7SPF1	Succinate dehydrogenase [ubiquinone] flavoprotein subunit, mitochondrial (8)	1.85
97	C5DB68	Pyruvate kinase (4, 11)	1.60
31	F6HGH4	6-phosphogluconate dehydrogenase, decarboxylating (7)	1.53
23	F6I0H8	UTP–glucose-1-phosphate uridylyl transferase (4)	1.44
42	D7T0U8	Glyceraldehyde-3-phosphate dehydrogenase (1)	−1.56
45	F6HFL6	Fructose-bisphosphate aldolase (4)	−1.72
13	A5B8T3	Fructokinase (2)	−1.86
72	D7TJI9	Pyruvate decarboxylase 1 (5)	−1.90
132	A5BEM8	Oxidoreductase GLYR1 (7)	−2.60
100	D7TR81	Pyrophosphate–fructose 6-phosphate 1-phosphotransferase subunit beta (4)	−2.96
46	F6I134	Triosephosphate isomerase, chloroplastic (1, 21)	−3.06
207	C0KY93	Leucoanthocyanidin dioxygenase (1, 7, 13, 16, 17, 26)	−3.29
192	F6HI27	Pyruvate dehydrogenase E1 component subunit beta-2, chloroplastic (1, 8, 11)	−3.75
265	F6GY71	Pyruvate decarboxylase 1 (5)	−18.60
*Cell Wall (10)*			
233	F6I390	Pectinesterase (10)	5.87
57	F6I6R4	Beta-xylosidase/alpha-L-arabinofuranosidase 2 (10)	1.44
*Lipid metabolism (11)*			
111	F6HXC8	Phospholipase D (11,1)	3.51
73	A5AS18	Putative quinone reductase (11)	−1.42
60	F6HLJ7	Enoyl-[acyl-carrier-protein] reductase [NADH] 1, chloroplastic isoform X1 (11)	−1.68
65	F6H9P9	Biotin carboxylase 1, chloroplastic ()	−1.76
168	D7STF0	3-hydroxyacyl-[acyl-carrier-protein] dehydratase FabZ (11)	−1.94
203	D7T4I1	Biotin carboxyl carrier protein of acetyl-CoA carboxylase 2, chloroplastic (11)	−2.98
88	D7TAP7	3-oxoacyl-[acyl-carrier-protein] reductase 2, chloroplastic (11, 26)	−3.19
125	A5C5V3	Dihydroceramide fatty acyl 2-hydroxylase FAH1 (11)	−7.02
141	D7TVI4	3-oxoacyl-[acyl-carrier-protein] synthase I, chloroplastic (11)	d.
*N and amino acid metabolism (12, 13)*			
231	F6HQA7	Nitrite reductase 1 (12)	4.11
25	A5C5K3	Adenosyl homocysteinase (13)	1.43
139	A5CAL1	Glyoxylate/hydroxypyruvate reductase A HPR2 (13, 1, 26)	−1.45
155	A5ATW2	Bifunctional 3-dehydroquinate dehydratase/shikimate dehydrogenase (13)	−1.61
26	P51119	Glutamine synthetase cytosolic isozyme 2 (12)	−1.72
160	D7SW04	Bifunctional aspartate aminotransferase and glutamate/aspartate-prephenate aminotransferase isoform X2 (13)	−1.80
*Secondary metabolism (16)*			
101	A0A0M5I8D0	Flavanone 3-hydroxylase (16, 17, 29)	−1.63
50	F6H775	Class I-like SAM-binding methyltransferase superfamily (16, 26)	−4.95
257	F6GX19	Isopentenyl-diphosphate Delta-isomerase I (16)	−6.85
213	A5BVM7	O-methyltransferase YrrM (16)	−7.23
*Hormone metabolism (17)*			
241	A5B174	Perakine reductase (17)	10.76
*Stress (20)*			
54	A3QRB7	Chitinase class I basic (20)	2.16
43	D7TS57	Chaperonin CPN60-2, mitochondrial (20, 29)	1.82
163	D7TKM8	Germin-like protein 2-1 (12, 20, 27, 30, 34)	1.59
110	D7T8R2	MLP-like protein 34 (20)	1.54
264	D7TNE5	Hypersensitive-induced response protein 1-like isoform X1 (20)	−1.64
252	A5AKX5	SOUL heme-binding protein (19, 29)	−1.71
71	F6GTP0	Heat shock protein, putative (20, 27)	−1.73
251	D7UE33	PLAT domain-containing protein 3-like (20)	−1.81
*Redox (21)*			
16	Q8S568	Catalase (21)	2.61
*Miscellaneous enzyme families (26)*			
263	F6GUF3	Peroxidase 53 (26)	New
218	F6HIC8	Dienelactone hydrolase (26)	1.42
48	F6I4V3	ADP-ribosylation factor 1-like 2 (26, 33)	−1.51
266	D7TUE8	Glycosyltransferase (26)	−1.56
86	F6GT84	Glutathione S-transferase U9 (26)	−1.66
36	F6HR72	Glutathione S-transferase (26)	−1.93
67	A0A024FS61	Polyphenol_oxidase (26)	−1.90
107	A5AZ36	Glutathione S-transferase U25 (26)	−3.16
234	D7TE48	Soluble epoxide hydrolase (26)	−4.66
167	F6HZD8	Short-chain dehydrogenase reductase 3b-like (26)	−10.74
188	D7T8G2	Purple acid phosphatase (26)	−54.69
*DNA/RNA (27,28)*			
225	A5ARE0	Glutelin type-A 1-like (28, 33)	7.40
28	D7TCM7	UPI00053F79C7 (*RNA helicase*) (27)	1.48
118	A5B427	Cyclase (28)	1.41
*Protein (29)*			
260	D7SIX7	Serine/threonine-prot. phosphatase 2A 65 kDa regulatory sub. A isoform (29)	New
105	F6I455	Probable elongation factor 1-gamma 2 (29)	4.34
30	F6H4T7	Elongation factor 2 (29)	2.16
80	A5BUU4	40S ribosomal protein SA (29)	1.95
134	E0CV68	Importin subunit beta-1 (29)	1.52
121	F6GZY7	Cysteine proteinase RD21A (29, 34)	−1.47
165	E0CR38	Proteasome subunit beta type (29)	−1.71
63	F6H7H1	Procardosin-A (29)	−2.12
185	F6H1D7	Carboxypeptidase (29)	−5.53
221	A5BIH7	Peptidase_S10 domain-containing protein (29)	−7.81
197	A5AKL4	Cysteine protease, putative (29, 34)	d.
201	D7TW90	Cucumsin (29)	d.
*Cell/signaling/development (30, 31, 33)*			
224	D7SJV3	Clathrin heavy chain (31)	New
12	A5BTZ8	Annexin (31)	1.43
8	F6I0I5	Actin-8 (31)	−1.42
190	D7T9L8	Coatomer subunit delta (31)	−2.20
*Transport (34)*			
39	F6HBF2	ADP, ATP carrier protein, mitochondrial (34, 2)	3.37
*Not assigned (35)*			
228	D7T9K4	Uncharacterized protein (35)	3.04
226	D7SJF5	Uncharacterized protein (35)	1.96
174	A5B729	Uncharacterized protein (35)	−21.21

Numbers reported in brackets refer to bin code (i.e., major functional categories). **#**: identification number. **f.c.**: bin code of functional categories. **ΔSS/C**: fold changes in salt-stressed plants compared to the control ones (up: %(SI)WS/%(SI)C, down: - %(SI)C/%(SI)WS). **new**: not present in the controls; **d.**: disappeared, not present in salt-stressed plants.

## Supplementary Materials

Supplementary Materials can be found at https://www.mdpi.com/1422-0067/21/3/1076/s1.
