# Supplementary material for: Root Proteomic Analysis of Two Grapevine Rootstock Genotypes Showing Different Susceptibility to Salt Stress
Source: Int J Mol Sci. 2020 Feb 6;21(3):1076. doi: 10.3390/ijms21031076 (PMC7037071; doi:10.3390/ijms21031076)
Supplement: Supplementary file 1 [file ijms-21-01076-s001.pdf]

SUPPLEMENTARY DATA 1

Root proteomic analysis of two grapevine rootstock genotypes showing different susceptibility to salt stress

Bhakti Prinsi, Osvaldo Failla, Attilio Scienza, Luca Espen

Department of Agricultural and Environmental Sciences—Production, Landscape, Agroenergy (DiSAA), Università degli Studi di Milano, Via Celoria 2, 20133 Milano, Italy

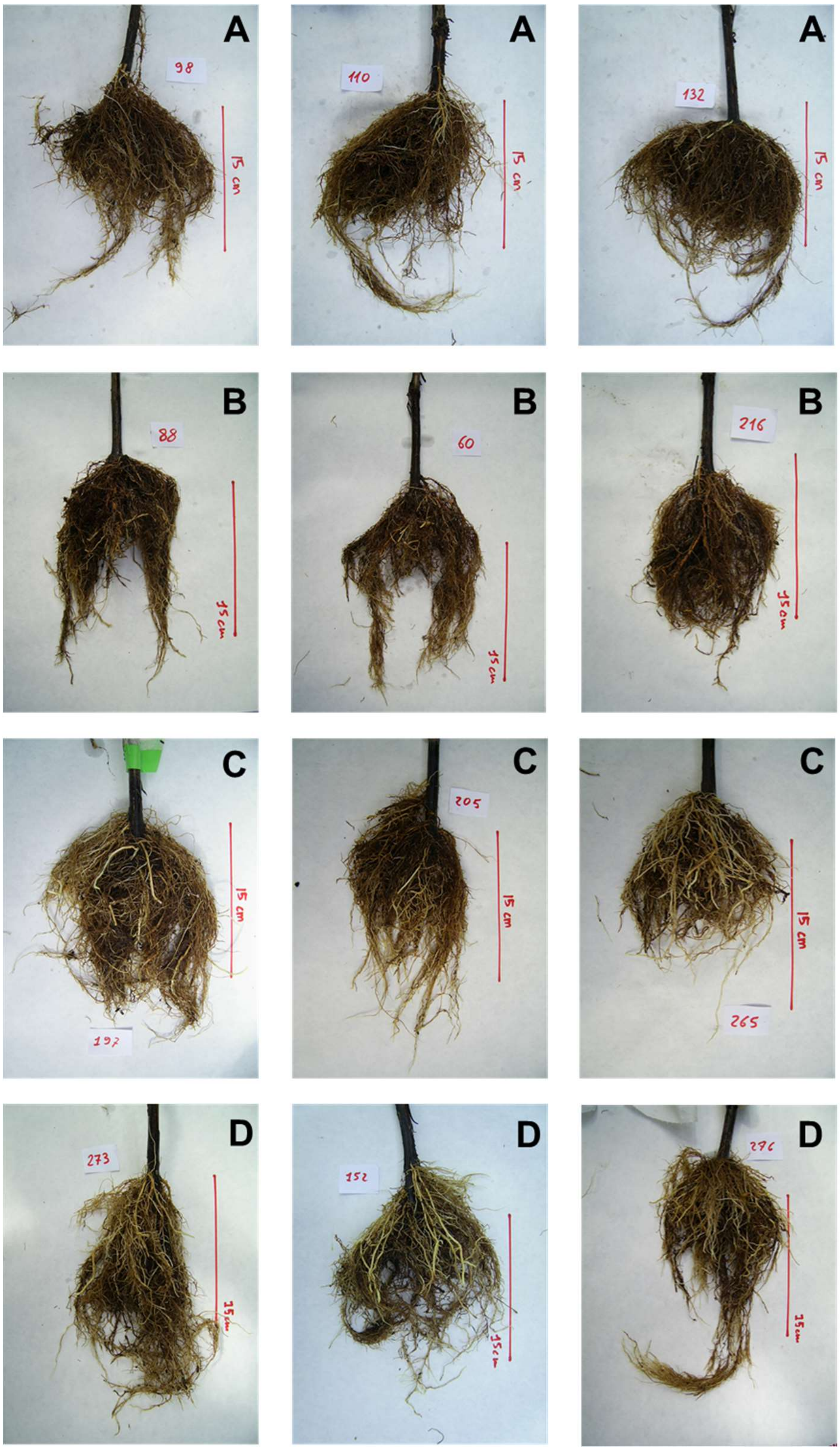

Figure S1. Comparison of morphology of the whole root organ in grapevine rootstocks in control and salt stress conditions. Roots of control (**A**) and salt stressed (**B**) plants of the 101.14 genotype; roots of control (**C**) and salt stressed **(D)** plants of the M4 genotype.

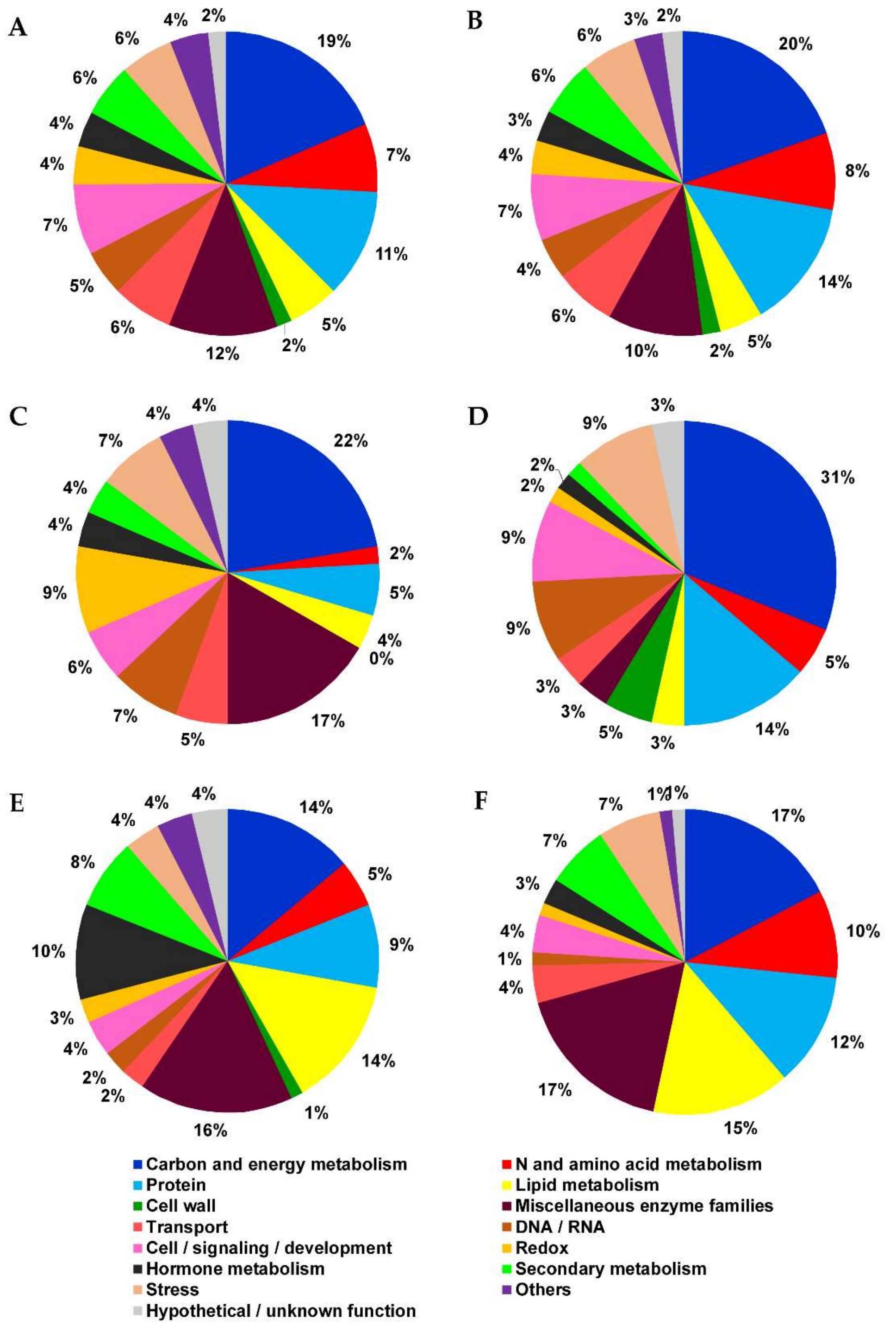

Figure S2. Functional distribution of the identified proteins in roots of 101.14 (**A**, **C,** and **E**) and M4 (**B**, **D,** and **F**) rootstocks. **A**, **B:** distribution of all the proteins identified in the control condition. **C, D:** proteins that increased in abundance or appeared in salt stress. **E, F:** proteins that decreased in abundance or disappeared in salt stress.

SUPPLEMENTARY DATA 1

Root proteomic analysis of two grapevine rootstock genotypes showing different susceptibility to salt stress.

Bhakti Prinsi, Osvaldo Failla, Attilio Scienza, Luca Espen
Department of Agricultural and Environmental Sciences—Production, Landscape, Agroenergy (DiSAA), Università degli Studi di Milano, Via Celoria 2, 20133 Milano, Italy

Table S1. Protein quantification by LC-ESI-MS/MS in the 101.14 root proteome. Protein quantification was obtained by summing the spectrum intensity (SI) of all the identified peptides in the protein. Protein abundance was normalized as the percentage with respect to the abundance of all validated proteins in the sample [%(SI)]. The results are the mean of three biological samples (n=3).

	Accession										Control		WS			
#	SwisProt	NCBI	GI	Name	BINCODE		Function	amino acid coverage %	# unique identified peptide	Protein MS/MS Search Score	Mean %(SI)	SE	Mean %(SI)	SE	Fold changes (ΔSS/C)	t-test
1	F6HNX5	XP_002283532.2	359486799	Heat shock cognate 70 kDa protein 2	20	20.2.1	stress.abiotic.heat	45,5	20	418,39	3,379	0,15412	3,136	0,08366	-1,08	0,195
2	F6GTT2	XP 002280824.1	225456079	ATP synthase subunit beta, mitochondrial	9	9.9	mitochondrial electron transport / ATP synthesis.F1-ATPase	52,3	19	386,97	4,392	0,27279	4,331	0,25703	-1,01	0,874
3	F6HMN8	XP_002276438.2	225439223	5-methyltetrahydropteroyltriglutamate--homocysteine methyltransferase	13	13.1.3.4.3	amino acid metabolism.synthesis.aspartate family.methionine.methionine synthase	30,4	19	309,15	1,414	0,09575	1,901	0,10931	1,34	0,007
4	F6GSG7	XP_002263145.2	359491599	Glyceraldehyde-3-phosphate dehydrogenase	4	4.1.8	glycolysis.cytosolic branch.glyceraldehyde 3-phosphate dehydrogenase (GAP-DH)	62,9	18	296,63	3,301	0,04052	3,537	0,08251	1,07	0,028
5	A5B118	CAN71905.1	147781269	Fructose-bisphosphate aldolase	1	1.3.6	PS.calvin cycle.aldolase	48	15	255,73	1,064	0,06759	1,180	0,05651	1,11	0,216
					4	4.1.10	glycolysis.cytosolic branch.aldolase									
6	D7SS06	CBI18438.3	297735751	V-type proton ATPase catalytic subunit A	34	34.1.1	transport.p- and v-ATPases.H+-transporting two-sector ATPase	31,1	15	247,87	0,710	0,00780	0,664	0,03338	-1,07	0,209
7	A0A1Z2THL4	CBI28009.3	297738764	NADP-dependent malic enzyme	8	8.2.10	TCA / organic transformation.other organic acid transformations.malic	35,8	15	233,13	0,514	0,08314	0,899	0,07845	1,75	0,007
8	F6H5R4	XP_002283198.1	225452767	Tubulin alpha chain	31	31.1	cell.organisation	38,2	13	232,54	2,079	0,10510	1,924	0,10254	-1,08	0,314
9	F6I0I5	XP_002282516.1	225431585	Actin-8	31	31.1	cell.organisation	32,8	13	214,27	2,771	0,04447	2,359	0,10913	-1,17	0,006
10	B6VJY3	YP_002608395.1	224365668	ATP synthase subunit alpha	9	9.9	mitochondrial electron transport / ATP synthesis.F1-ATPase	24,9	13	212,53	1,779	0,01909	1,637	0,06214	-1,09	0,055
11	A5C3B4	CAN81774.1	147856322	14-3-3-like protein GF14 psi	30	30.7	signalling.14-3-3 proteins	48,4	13	210,84	1,912	0,06578	1,677	0,07690	-1,14	0,043
12	Q8S568	Q8S568	75303534	Catalase	21	21.6	redox.dismutases and catalases	31,7	13	208,91	0,814	0,04350	1,374	0,08150	1,69	0,000
13	A5ATG8	XP_002283056.1	225440131	Tubulin beta chain	31	31.1	cell.organisation	35,5	13	204,5	1,256	0,05591	1,118	0,04024	-1,12	0,074
14	A5B8T3	CAN67659.1	147820522	Fructokinase-4	2	2.2.1.1	major CHO metabolism.degradation.sucrose.fructokinase	41,5	12	211,13	1,099	0,01458	0,790	0,03719	-1,39	0,000
15	A5B038	AAZ79355.1	73647513	Aldehyde dehydrogenase	5	5.10	fermentation.aldehyde dehydrogenase	28	12	175,26	0,427	0,04479	0,345	0,01421	-1,24	0,110
					29	29.2.2	protein.synthesis.ribosome biogenesis									
16	D7TFJ4	XP_002271514.1	225425280	Transaldolase	7	7.2.2	OPP.non-reductive PP.transaldolase	33,1	11	214,58	1,021	0,06367	0,930	0,05049	-1,10	0,293
17	F6H5H5	XP_002278190.1	225446489	Chavicol O-methyltransferase	16	16.2	secondary metabolism.phenylpropanoids	36,6	11	204,98	2,534	0,18802	2,246	0,08839	-1,13	0,195
18	F6HKH3	XP_002283632.1	225441000	Enolase 1	4	4.1.11	glycolysis.cytosolic branch.3-phosphoglycerate kinase (PGK)	33,7	11	190	1,190	0,15805	1,595	0,09916	1,34	0,055
19	D7SYK8	XP_002278181.1	225431960	ATP-citrate synthase beta chain protein 2	8	8.2.11	TCA / organic transformation.other organic acid transformations.atp-citrate lyase	28,7	11	189,64	0,647	0,03104	0,659	0,01806	1,02	0,745
20	A5BTZ8	CAN81470.1	147861246	Annexin	31	31.1	cell.organisation	37,2	11	185,49	1,031	0,03900	1,197	0,02871	1,16	0,007
21	F6I0H8	XP_002282276.1	225431563	UTP--glucose-1-phosphate uridylyltransferase	4	4.1.1	glycolysis.cytosolic branch.UGPase	27,6	11	183,69	0,569	0,06240	0,788	0,04379	1,38	0,017
22	Q9M563	ABB82365.1	82547239	Beta-1,3-glucanase	26	26.4.1	misc.beta 1,3 glucan hydrolases.glucan endo-1,3-beta-glucosidase	38	11	182,93	0,521	0,06077	0,989	0,04663	1,90	0,000
23	E0CQW9	XP_002285897.1	225459744	V-type proton ATPase subunit B1	34	34.1.1.1	transport.p- and v-ATPases.H+-transporting two-sector ATPase.subunit B	25	11	174,35	0,420	0,03532	0,449	0,03076	1,07	0,547
24	F6HFL6	XP_010662289.1	731369971	Fructose-bisphosphate aldolase	4	4.1.10	glycolysis.cytosolic branch.aldolase	32,3	11	143,18	0,696	0,06400	0,437	0,03134	-1,59	0,005
25	A0A1Z2THL9	ASA69248.1	1207771375	Malate dehydrogenase	8	8.1.9	TCA / organic transformation.TCA.malate DH	55,1	10	194,99	1,715	0,18684	1,330	0,10102	-1,29	0,100
26	F6HZK0	XP_002278712.1	225438145	Malate dehydrogenase, cytoplasmic	8	8.2.9	TCA / organic transformation.other organic acid transformations.cyt MDH	38,6	10	192,91	1,590	0,05346	1,626	0,10466	1,02	0,765
27	F6GWF3	XP_002266276.1	225433510	Serine hydroxymethyltransferase	1	1.2.5	PS.photorespiration.serine hydroxymethyltransferase	25	10	169,16	0,368	0,01973	0,549	0,04494	1,49	0,004
					13	13.1.5.2	amino acid metabolism.synthesis.serine-glycine-cysteine group.glycine									
28	F6HNF4	XP_002272669.1	225449018	adenosine kinase 2	23	23.3.2.1	nucleotide metabolism.salvage.nucleoside kinases.adenosine kinase	43,4	10	167,69	0,258	0,01840	0,291	0,02542	1,13	0,323
29	E0CR49	CBI19003.3	302141800	Protein disulfide-isomerase (21)	21	21.1	redox.thioredoxin	28,8	10	167,33	0,192	0,02064	0,347	0,05476	1,81	0,024
30	F6HR72	XP_002262767.1	225462609	Glutathione S-transferase	26	26.9	misc.glutathione S transferases	40,1	10	160,55	0,815	0,12380	0,490	0,03266	-1,66	0,029
31	F6GTE2	XP_002263490.1	225456550	Alpha-1,4-glucan-protein synthase [UDP-forming], putative	10	10.5.5	cell wall.cell wall proteins.RGP	37,4	10	159,33	0,516	0,05541	0,511	0,05432	-1,01	0,952
32	D7TCM7	CBI27885.3	297738640	UPI00053F79C7	27	27.1.2	RNA.processing.RNA helicase	24,4	10	144,37	0,457	0,04424	0,432	0,03781	-1,06	0,672
33	D7T7Y3	CBI26642.3	297737441	Aconitate hydratase 3, mitochondrial	8	8.1.3	TCA / organic transformation.TCA.aconitase	14	10	136,59	0,412	0,03362	0,546	0,04235	1,33	0,033
34	F6HTU8	XP_002275990.1	225451235	Cysteine synthase	13	13.1.5.3.1	amino acid metabolism.synthesis.serine-glycine-cysteine group.cysteine.OASTL	20,3	9	178,25	0,987	0,05159	0,745	0,05072	-1,33	0,007
35	A5CAF6	XP_002263950.1	225464999	Phosphoglycerate kinase	4	4.1.11	glycolysis.cytosolic branch.3-phosphoglycerate kinase (PGK)	31,1	9	154,39	0,709	0,02308	0,744	0,02211	1,05	0,292
36	P51119	P51119.1	1707959	Glutamine synthetase cytosolic isozyme 2	12	12.2.2	N-metabolism.ammonia metabolism.glutamine synthetase	33,7	9	141,48	0,953	0,06978	0,619	0,05193	-1,54	0,003
37	F6HUS6	XP_003633800.1	359488672	Uncharacterized protein	35	35.2	not assigned.unknown	11,1	9	138,65	0,276	0,01830	0,490	0,02896	1,78	0,000
38	F6GT74	XP_002284909.1	225457407	Malate dehydrogenase, mitochondrial	8	8.1.9	TCA / organic transformation.TCA.malate DH	32,4	9	138,11	0,742	0,06530	0,651	0,06886	-1,14	0,362
39	Q3KN68	NP_001268171.1	526118006	Isoflavone reductase-like protein 5	16	16.8.5.1	secondary metabolism.flavonoids.isoflavonols.isoflavone reductase	33,9	9	134,32	0,929	0,04398	1,070	0,03873	1,15	0,037
40	A5C5K3	CAN64242.1	147783188	Adenosylhomocysteinase	13	13.2.3.4	amino acid metabolism.degradation.aspartate family.methionine	23,2	9	129,82	0,346	0,01905	0,410	0,03245	1,19	0,117
41	F6H9P9	CBI24059.3	297736021	Biotin carboxylase 1, chloroplastic (11)	11	11.1.1.2.4	lipid metabolism.FA synthesis and FA elongation.acetyl CoA carboxylation.heteromeric complex.biotin carboxylase	20,5	9	127,05	0,550	0,03276	0,214	0,01919	-2,57	0,000
42	F6HYJ1	XP_002276114.1	225445867	Leucine aminopeptidase 1 isoform X1	29	29.5	protein.degradation	24	9	116,63	0,208	0,02915	0,255	0,01239	1,23	0,166
43	F6H440	XP_002275325.1	225451581	Mitochondrial phosphate transporter	34	34.9	transport.metabolite transporters at the mitochondrial membrane	24,2	9	114,64	0,570	0,07413	0,546	0,03314	-1,04	0,773
44	A5JPK7	NP_001267971.1	526118253	Monodehydroascorbate reductase (31)	21	21.2.1	redox.ascorbate and glutathione.ascorbate	1,427	8	153,21	0,447	0,02953	0,638	0,03980	1,43	0,003
45	F6HDW4	XP_002283898.1	225432858	GDP-mannose 3,5-epimerase	10	10.1.5	cell wall.precursor synthesis.UXS	25,4	8	146,85	0,497	0,02665	0,310	0,03508	-1,61	0,002
					21	21.2.1.1	redox.ascorbate and glutathione.ascorbate.GME									
46	F6HGH4	XP_002270984	225425053	6-phosphogluconate dehydrogenase, decarboxylating	7	7.1.3	OPP.oxidative PP.6-phosphogluconate dehydrogenase	17,4	8	124,97	0,439	0,03514	0,553	0,02342	1,26	0,023
47	F6H775	NP_001268100.1	526117731	Class I-like SAM-binding methyltransferase superfamily	16	16.2.1.9	secondary metabolism.phenylpropanoids.lignin biosynthesis.COMT	13,1	8	122,57	0,231	0,02044	0,279	0,01422	1,20	0,087
					26	26.6	misc.O-methyl transferases									
48	F6GWA8	XP_002277357.2	359479362	Chaperonin 60 subunit alpha 2, chloroplastic	1	1.3.13	PS.calvin cycle.rubisco interacting	16,2	8	118,47	0,651	0,12931	0,197	0,03397	-3,30	0,007
					29	29.6	protein.folding									
49	F6H3U9	XP_002285385.1	225430398	Aspartate aminotransferase	13	13.1.1.2.1	amino acid metabolism.synthesis.central amino acid metabolism.aspartate.aspartate aminotransferase	20,1	8	106,57	0,187	0,01626	0,255	0,01359	1,36	0,009
50	F6HFF7	XP_002284729.1	225424316	Phosphoglucomutase, cytoplasmic 1	4	4.1.2	glycolysis.cytosolic branch.phosphoglucomutase (PGM)	16,4	8	97,34	0,315	0,08220	0,160	0,04984	-1,97	0,137
51	A3QRB7	NP_001267891.1	526117918	Chitinase class I basic (20)	20	20.1.7	stress.biotic.PR-proteins	31,2	7	139,14	0,575	0,04377	1,423	0,06894	2,47	0,000
52	A5BV65	CAN70587.1	147784332	Triosephosphate isomerase	4	4.1.7	glycolysis.cytosolic branch.triosephosphate isomerase (TPI)	46,8	7	136,08	0,751	0,09327	0,726	0,07976	-1,03	0,843
53	F6I4V3	CBI39638.3	296089819	ADP-ribosylation factor 1-like 2	26	26.1	misc.misc2	25	7	130,02	0,848	0,05140	0,670	0,01449	-1,26	0,008
54	F6HAM6	XP_002266494.2	359490179	Transketolase, chloroplastic	7	7.2.1	OPP.non-reductive PP.transketolase	13	7	124,64	0,656	0,10706	0,640	0,11728	-1,03	0,920

**55**	F6HBF2	XP_002279748.1	225450149	ADP,ATP carrier protein, mitochondrial	2	2.1.2.5	major CHO metabolism.synthesis.starch.transporter	14,8	7	124,42	**0,815**	0,14562	**1,047**	0,17144	**1,28**	**0,326**
					34	34.14	transport.unspecified cations									
**56**	F6H7H1	XP_003632941.1	359483345	Procardosin-A	29	29.5.4	protein.degradation.aspartate protease	16,6	7	117,05	**0,892**	0,06870	**0,637**	0,07405	**-1,40**	**0,030**
**57**	F6I5Y5	XP_002285358.1	225428898	D-3-phosphoglycerate dehydrogenase	13	13.1.5.1.1	amino acid metabolism.synthesis.serine-glycine-cysteine group.serine.phosphoglycerate dehydrogenase	19,7	7	112,39	**0,408**	0,04331	**0,573**	0,06728	**1,40**	**0,067**
**58**	C5DB68	CAQ58629.1	239056192	Pyruvate kinase, cytosolic isozyme	4	4.1.14	glycolysis.cytosolic branch.pyruvate kinase (PK)	20,7	7	109,62	**0,202**	0,02846	**0,344**	0,03037	**1,70**	**0,007**
					11	11.1.30	lipid metabolism.FA synthesis and FA elongation.pyruvate kinase									
**59**	A5C8L8	CAN64338.1	147834040	Monodehydroascorbate reductase 5, mitochondrial isoform X1	21	21.2.1	redox.ascorbate and glutathione.ascorbate	10,9	7	108,8	**0,171**	0,02492	**0,315**	0,01105	**1,84**	**0,000**
**60**	F6GTG3	XP_002274334.1	225455784	Enolase 1, chloroplastic-like	4	4.1.13	glycolysis.cytosolic branch.enolase	18,6	7	108,5	**0,194**	0,02069	**0,029**	0,02397	**-6,75**	**0,000**
**61**	D7T0U8	CBI24168.3	297736130	Glyceraldehyde-3-phosphate dehydrogenase (13)	1	1.3.4	PS.calvin cycle.GAP	19,8	7	107,92	**0,587**	0,03708	**0,331**	0,01488	**-1,77**	**0,000**
**62**	F6HPC0	XP_002275338.2	359473386	Glutathione S-transferase U10	26	26.9	misc.glutathione S transferases	29,5	7	107,16	**0,450**	0,08209	**0,559**	0,03468	**1,24**	**0,250**
**63**	A5BX54	CBI27352.3	297738151	Isocitrate dehydrogenase [NADP]	8	8.1.4	TCA / organic transformation.TCA.IDH	20,1	7	100,89	**0,218**	0,03215	**0,238**	0,02340	**1,09**	**0,624**
**64**	F6I0F6	XP_002281334.1	225431501	Alcohol dehydrogenase	5	5.3	fermentation.ADH	11,5	7	100,37	**0,231**	0,02782	**0,412**	0,04691	**1,78**	**0,008**
					26	26.11.1	misc.alcohol dehydrogenases.cinnamyl alcohol dehydrogenase									
**65**	A5C7J5	CAN70962.1	147821099	Thioredoxin family protein	24	21.1	redox.thioredoxin	26	7	100,19	**0,285**	0,02694	**0,268**	0,02785	**-1,07**	**0,660**
**66**	A5C4C2	XP_002279598.1	225439902	Elongation factor 1-alpha	29	29.2.4	protein.synthesis.elongation	21	7	80,57	**0,428**	0,03138	**0,395**	0,03611	**-1,08**	**0,511**
**67**	A5AS18	CBI20462.3	302143167	Quinone reductase	11	11.8	lipid metabolism.exotics (steroids, squalene etc)	40,3	6	122,94	**0,815**	0,08083	**0,656**	0,06267	**-1,24**	**0,152**
**68**	A5ANT9	A5ANT9.1	158514257	Chalcone--flavonone isomerase 2	16	16.8.2.2	secondary metabolism.flavonoids.chalcones.chalcone isomerase	34,1	6	117,26	**0,557**	0,04664	**0,584**	0,05116	**1,05**	**0,705**
**69**	F6HNS4	CBI31594.3	296086153	Peptidase	29	29.5.1	protein.degradation.subtilases	6,6	6	107,99	**0,750**	0,03852	**0,739**	0,05948	**-1,01**	**0,887**
**70**	F6HLJ7	XP_019077386.1	1105499058	Enoyl-[acyl-carrier-protein] reductase [NADH] 1, chloroplastic	11	11.1.6	lipid metabolism.FA synthesis and FA elongation.enoyl ACP reductase	19,9	6	104,67	**0,462**	0,02604	**0,217**	0,01930	**-2,13**	**0,000**
**71**	F6H4T7	XP_002266780	225462164	Elongation factor 2	29	29.2.4	protein.synthesis.elongation	10,3	6	101,05	**0,587**	0,18821	**1,014**	0,12032	**1,73**	**0,085**
**72**	F6HJH9	XP_002264055.2	359489076	Glutathione S-transferase	26	26.9	misc.glutathione S transferases	26,4	6	98,72	**0,653**	0,02665	**0,611**	0,04067	**-1,07**	**0,417**
**73**	A5AKB1	CAN69132.1	147787229	Plastid-lipid-associated protein 1, chloroplastic	31	31.1	cell.organisation	39,5	6	96,98	**0,174**	0,01861	**0,094**	0,01907	**-1,85**	**0,013**
**74**	A5BVL9	CAN63896.1	147799894	Glutathione S-transferase GST 19	26	26.9	misc.glutathione S transferases	25,3	6	96,25	**0,414**	0,01924	**0,551**	0,02646	**1,33**	**0,002**
					28	28.1	DNA.synthesis/chromatin structure									
					33	33.99	development.unspecified									
**75**	C5DB50	XP_002266205.1	225439064	2,3-bisphosphoglycerate-independent phosphoglycerate mutase	4	4.1.12	glycolysis.cytosolic branch.phosphoglycerate mutase	11,9	6	95,51	**0,298**	0,01915	**0,330**	0,02607	**1,11**	**0,341**
**76**	F6HVD5	XP_002264626.1	225465837	Aspartyl protease AED3	27	27.3.67	RNA.regulation of transcription.putative transcription regulator	18,5	6	93,03	**0,380**	0,04361	**0,457**	0,01251	**1,20**	**0,120**
**77**	A5BUU4	A5BUU4.1	229891614	40S ribosomal protein SA	29	29.2.1.2.1.31	protein.synthesis.ribosomal protein.eukaryotic.40S subunit.SA	23	6	92,71	**0,292**	0,01596	**0,255**	0,03702	**-1,15**	**0,378**
					29	29.2.2	protein.synthesis.ribosome biogenesis									
**78**	F6H5Z7	CBI22354.3	302143601	UPI0008FECB57 - Ubiquitin	29	29.2.2	protein.synthesis.ribosome biogenesis	5,9	6	91,43	**0,745**	0,05126	**0,723**	0,03308	**-1,03**	**0,730**
**79**	A5BH43	CAN63486.1	147811059	Carboxypeptidase	29	29.5.5	protein.degradation.serine protease	15,5	6	90,54	**0,219**	0,01333	**0,276**	0,04139	**1,26**	**0,219**
**80**	A5AKK0	CAN75773.1	147789187	Putative ripening-related protein	20	20.1	stress.biotic	35,7	6	90,53	**0,416**	0,01779	**0,334**	0,03460	**-1,25**	**0,061**
**81**	F6I134	XP_002274871.1	225427917	Triosephosphate isomerase, chloroplastic	1	1.3.5	PS.calvin cycle.TPI	26,2	6	89,29	**0,223**	0,05682	**0,108**	0,02067	**-2,06**	**0,086**
**82**	F6H5H9	XP_002278316.1	225446496		16	16.2	secondary metabolism.phenylpropanoids	21,4	6	86,11	**0,428**	0,03462	**0,516**	0,03668	**1,20**	**0,114**
					26	26.6	misc.O-methyl transferases									
**83**	F6HHQ7	XP_002265690.1	225447510	Acetyl-CoA acetyltransferase 1	13	13.2.3.5	amino acid metabolism.degradation.aspartate family.lysine	22,8	6	82,02	**0,177**	0,02954	**0,220**	0,01383	**1,25**	**0,211**
					13	13.2.4.5	amino acid metabolism.degradation.branched chain group.isoleucine									
					16	16.1.2.1	secondary metabolism.isoprenoids.mevalonate pathway.acetyl-CoA C-acyltransferase									
**84**	F6GTA6	XP_002272188.1	225456674	Hypersensitive-induced response protein 1 isoform X2	20	20.1.99	stress.biotic.misc	19	6	81,27	**0,573**	0,04627	**0,445**	0,03628	**-1,29**	**0,056**
**85**	D7TUK1	XP_002278850.1	225451316	Perakine reductase isoform X1 (auxin-induced protein PCNT115 isoform 1)	17	17.2.3	hormone metabolism.auxin.induced-regulated-responsive-activated	26,8	6	79,25	**0,035**	0,01682	**0,202**	0,02830	**5,71**	**0,000**
					26	26.10	misc.cytochrome P450									
**86**	D7TJI9	CBI30661.3	297740479	Pyruvate decarboxylase 1	5	5.2	fermentation.PDC	10,7	6	73,77	**0,236**	0,02455	**0,336**	0,02787	**1,42**	**0,023**
**87**	F6HHU9	CAN77321.1	147799889	Uncharacterized protein	35	35.1.26	not assigned.no ontology.DC1 domain containing protein	11,3	6	66,25	**0,014**	0,00820	**0,236**	0,03538	**16,51**	**0,000**
**88**	F6GTP0	XP_002279101.1	225456004	Heat shock protein, putative	20	20.1.5	stress.biotic.regulation of transcription	9,4	6	60,85	**0,130**	0,01671	**0,165**	0,02161	**1,27**	**0,229**
					20	20.2.1	stress.abiotic.heat									
					27	27.3.3	RNA.regulation of transcription.AP2/EREBP, APETALA2/ethylene-responsive element binding protein family									
**89**	A5BXT5	CBI24770.3	296084382	Guanosine nucleotide diphosphate dissociation inhibitor (30)	30	30.5	signalling.G-proteins	16,6	6	60,29	**0,036**	0,01189	**0,073**	0,00917	**2,05**	**0,032**
**90**	A5AKD8	CAN64643.1	147787082	Peptidyl-prolyl cis-trans isomerase (26)	26	26.8	misc.nitrilases, nitrile lyases, berberine bridge enzymes, reticuline oxidases, troponine reductases	29,6	5	103,56	**1,738**	0,10035	**2,508**	0,02576	**1,44**	**0,000**
**91**	Q2HZF5	NP_001268056.1	526117561	Aquaporin PIP2	34	34.19.1	transport.major intrinsic proteins.PIP	32,5	5	95,67	**1,369**	0,07939	**1,362**	0,06098	**-1,01**	**0,945**
**92**	A3FA65	NP_001267921.1	526118028	Aquaporin PIP1;3	34	34.19.1	transport.major intrinsic proteins.PIP	23,6	5	91,36	**0,336**	0,04360	**0,373**	0,05016	**1,11**	**0,593**
**93**	A0A0M5I8D0	NP_001268034.1	526117485	Flavanone 3-hydroxylase	16	16.8.3.2	secondary metabolism.flavonoids.dihydroflavonols.flavanone 3-hydroxylase	20,9	5	86,18	**0,306**	0,02999	**0,172**	0,01384	**-1,78**	**0,002**
**94**	F6GWS4	XP_002275309.1	225431269	Peroxidase	26	26.12	misc.peroxidases	20,1	5	83,59	**0,045**	0,01835	**0,221**	0,02749	**4,88**	**0,000**
**95**	A0A024FS61	BAO79387.1	633259653	Polyphenol_oxidase	26	26.7	misc.oxidases - copper, flavone etc	8,6	5	83,54	**0,692**	0,02570	**0,602**	0,04184	**-1,15**	**0,098**
**96**	A5ASS2	CAN71616.1	147797244	Thaumatin	20	20.1	stress.biotic	27,1	5	81,37	**0,306**	0,03366	**0,489**	0,06507	**1,60**	**0,031**
**97**	Q1AFF4	AAZ79357.1	73647738	Ascorbate peroxidase	21	21.2.1	redox.ascorbate and glutathione.ascorbate	26	5	81,19	**0,403**	0,05017	**0,410**	0,02227	**1,02**	**0,912**
**98**	A5AVX9	XP_010661131.1	731419756	UDP-glucose 6-dehydrogenase	10	10.1.4	cell wall.precursor synthesis.UGD	13,3	5	80,38	**0,115**	0,01619	**0,089**	0,01608	**-1,29**	**0,281**
**99**	D7TBD9	XP_002283310.1	225445041	Mitochondrial-processing peptidase subunit alpha-2, chloroplastic/mitochondrial	29	29.3.2	protein.targeting.mitochondria	16,4	5	79,44	**0,155**	0,02455	**0,120**	0,01718	**-1,29**	**0,267**
**100**	F6HXC8	NP_001267897.1	526117940	Phospholipase D	1	1.1.5.1	PS.lightreaction.other electron carrier (ox/red).plastocyanin	8,2	5	77,37	**0,031**	0,01087	**0,120**	0,01327	**3,90**	**0,000**
					11	11.9.3.1	lipid metabolism.lipid degradation.lysophospholipases.phospholipase D									
					27	27.3.29	RNA.regulation of transcription.TCP transcription factor family									
**101**	A5BEM8	CBI25677.3	297736660	Putative oxidoreductase GLYR1	7	7.1.3	OPP.oxidative PP.6-phosphogluconate dehydrogenase	26,1	5	75,31	**0,214**	0,01690	**0,150**	0,02172	**-1,43**	**0,042**
**102**	F6GUN2	XP_002285722.1	225435760	Mitochondrial dicarboxylate/tricarboxylate transporter DTC	34	34.9	transport.metabolite transporters at the mitochondrial membrane	16,2	5	75,25	**0,199**	0,02471	**0,213**	0,01640	**1,07**	**0,641**
**103**	E0CV68	CBI23029.3	302143924	Importin subunit beta-1	29	29.3.1	protein.targeting.nucleus	11,4	5	73,41	**0,175**	0,05063	**0,293**	0,06079	**1,68**	**0,165**
**104**	D7TE48	CBI28771.3	297739120	Soluble epoxide hydrolase	26	26.1	misc.misc2	21	5	73,15	**0,198**	0,04084	**0,082**	0,01688	**-2,41**	**0,026**
**105**	D7TAP7	CBI27570.3	297738369	3-oxoacyl-[acyl-carrier-protein] reductase 2, chloroplastic	11	11.1.4	lipid metabolism.FA synthesis and FA elongation.ACP oxoacyl reductase	20,3	5	71,36	**0,176**	0,02926	**0,014**	0,01095	**-12,24**	**0,000**
					26	26.8	misc.nitrilases, nitrile lyases, berberine bridge enzymes, reticuline oxidases, troponine reductases									
					26	26.22	misc.short chain dehydrogenase/reductase (SDR)									
**106**	D7TVV9	XP_002277375.1	225426492	Cinnamyl alcohol dehydrogenase 1 isoform X1	15	15.2	metal handling.binding, chelation and storage	18	5	70,72	**0,122**	0,00834	**0,166**	0,03288	**1,36**	**0,220**
					16	16.2.1.10	secondary metabolism.phenylpropanoids.lignin biosynthesis.CAD									

**107**	D7TQB0	CBI32630.3	297741498	glutathione S-transferase	26	26.9	misc.glutathione S transferases	22,3	5	70,29	**0,150**	0,02559	**0,150**	0,02320	**-1,00**	**0,996**
**108**	F6H9T6	XP_002265514.1	225462297	Succinate-semialdehyde dehydrogenase, mitochondrial	8	8.1.99	TCA / organic transformation.TCA.misc	12,2	5	69,28	**0,215**	0,04995	**0,175**	0,05096	**-1,23**	**0,586**
					8	8.2.99	TCA / organic transformation.other organic acid transformations.misc									
**109**	D7SZ58	CBI23542.3	296083547	Ras-related protein RABD2b	30	30.5	signalling.G-proteins	32,8	5	66,57	**0,366**	0,05570	**0,379**	0,02830	**1,03**	**0,847**
**110**	F6GSZ7	XP_003634222.1	359491108	Omega-hydroxypalmitate O-feruloyl transferase	16	16.2	secondary metabolism.phenylpropanoids	10,2	5	66,08	**0,139**	0,02191	**0,176**	0,02385	**1,27**	**0,279**
**111**	D7UC33	XP_002267348.2	359489616	UPI0008FEC8A7 - PREDICTED: (+)-neomenthol dehydrogenase isoform X1	26	26.22	misc.short chain dehydrogenase/reductase (SDR)	25,2	5	65,64	**0,291**	0,03929	**0,251**	0,01986	**-1,16**	**0,386**
**112**	A5AXI6	CAN80537.1	147843260	60S acidic ribosomal protein P0	29	29.2.1.2.2.80	protein.synthesis.ribosomal protein.eukaryotic.60S subunit.P0	29,3	5	65,58	**0,459**	0,04303	**0,331**	0,04175	**-1,39**	**0,059**
**113**	F6HIK4	CBI28956.3	297739305	Peroxidase	26	26.12	misc.peroxidases	8	5	64,86	**0,110**	0,01983	**0,053**	0,02340	**-2,09**	**0,091**
**114**	F6I4H0	XP_010659744.1	731416007	S-adenosylmethionine synthase 4	13	13.1.3.4.11	amino acid metabolism.synthesis.aspartate family.methionine.S-adenosylmethionine synthetase	14	5	63,35	**0,132**	0,04234	**0,054**	0,01238	**-2,45**	**0,108**
**115**	F6H0X2	NP_001268096.1	526117715	Phospho-2-dehydro-3-deoxyheptonate aldolase	13	13.1.6.1.1	amino acid metabolism.synthesis.aromatic aa.chorismate.3-deoxy-D-arabino-heptulosonate 7-phosphate synthase	11,6	5	61,54	**0,059**	0,01776	**0,066**	0,01427	**1,11**	**0,783**
					34	34.16	transport.ABC transporters and multidrug resistance systems									
**116**	A5CAL1	CAN84153.1	147843670	Glyoxylate/hydroxypyruvate reductase A HPR2	1	1.2.6	PS.photorespiration.hydroxypyruvate reductase	19,8	5	61,36	**0,153**	0,00674	**0,059**	0,00671	**-2,59**	**0,000**
					13	13.2.5.2	amino acid metabolism.degradation.serine-glycine-cysteine group.glycine									
					26	26.1	misc.misc2									
**117**	F6KV60	XP_002274242.1	225431844	Pathogenesis-related protein 10	20	20.1.7	stress.biotic.PR-proteins	32,2	4	86,47	**2,282**	0,04048	**2,717**	0,03901	**1,19**	**0,000**
					27	27.1.19	RNA.processing.ribonucleases									
					34	34.16	transport.ABC transporters and multidrug resistance systems									
**118**	A0A0F7J172	ABH09331.1	111379088	Aquaporin TIP2;1	34	34.19.2	transport.major intrinsic proteins.TIP	23,2	4	82,27	**1,615**	0,27746	**1,362**	0,10951	**-1,19**	**0,417**
**119**	A0T2X7	ABK81651.1	118406886	Glutathione S-transferase	26	26.9	misc.glutathione S transferases	23,3	4	79,44	**0,976**	0,07969	**0,994**	0,08535	**1,02**	**0,879**
**120**	F6HFN8	XP_002282287.1	225423947	Dihydrolipoamide acetyltransferase component of pyruvate dehydrogenase complex	8	8.1.1.2	TCA / organic transformation.TCA.pyruvate DH.E2	11,4	4	78,02	**0,263**	0,04278	**0,151**	0,01966	**-1,74**	**0,039**
					11	11.1.31	lipid metabolism.FA synthesis and FA elongation.pyruvate DH									
**121**	A5B427	CAN60921.1	147838052	Cyclase	28	28.99	DNA.unspecified	15,8	4	74,46	**0,285**	0,00824	**0,401**	0,00390	**1,41**	**0,000**
**122**	D7SLM9	CBI16557.3	297746501	Chaperonin 60 subunit beta 2, chloroplastic	1	1.3.13	PS.calvin cycle.rubisco interacting	8	4	74,05	**0,138**	0,01516	**0,040**	0,00678	**-3,48**	**0,000**
					29	29.6	protein.folding									
**123**	D7TRS2	XP_002272479.1	225469754	Mannitol dehydrogenase	16	16.2.1.10	secondary metabolism.phenylpropanoids.lignin biosynthesis.CAD	17,7	4	73,7	**0,407**	0,05758	**0,555**	0,08396	**1,36**	**0,178**
**124**	F6HC36	XP_002280158.1	225448675	Chalcone-flavonone isomerase family protein	16	16.8.2	secondary metabolism.flavonoids.chalcones	24,2	4	72,79	**0,284**	0,03334	**0,236**	0,03262	**-1,20**	**0,329**
**125**	F6HPF2	XP_002276636.1	225424908	Mitochondrial outer membrane protein porin 1	34	34.20	transport.porins	19,2	4	72,61	**0,260**	0,00541	**0,268**	0,00890	**1,03**	**0,459**
**126**	F6I6R4	XP_002264183.2	359485890	Beta-xylosidase/alpha-L-arabinofuranosidase 2	10	10.6.1	cell wall.degradation.cellulases and beta-1,4-glucanases	6,9	4	72,01	**0,193**	0,01601	**0,152**	0,01508	**-1,27**	**0,093**
					10	10.6.2	cell wall.degradation.mannan-xylose-arabinose-fucose									
**127**	A5BM68	XP_010649714.1	731388721	Translationally-controlled tumor protein homolog	33	33.99	development.unspecified	28,1	4	70,06	**0,300**	0,04449	**0,199**	0,02423	**-1,51**	**0,074**
**128**	A5BAY1	CAN61170.1	147781964	Germin-like protein 9-2	15	15.2	metal handling.binding, chelation and storage	34,6	4	68,85	**0,366**	0,03741	**0,258**	0,02199	**-1,42**	**0,032**
					20	20.2.99	stress.abiotic.unspecified									
**129**	A5AGI7	CAN66724.1	147767381	Citrate synthase	8	8.1.2	TCA / organic transformation.TCA.CS	6,1	4	65,93	**0,149**	0,02329	**0,175**	0,01810	**1,17**	**0,406**
**130**	D7TS82	XP_002285653.1	225433424	Acetyl-CoA C-acyltransferase - 3-ketoacyl-CoA thiolase 2, peroxisomal	11	11.9.4.5	lipid metabolism.lipid degradation.beta-oxidation.acyl-CoA thioesterase	15,6	4	65,92	**0,052**	0,01342	**0,117**	0,02653	**2,23**	**0,056**
					13	13.2.4.1	amino acid metabolism.degradation.branched chain group.shared									
					13	13.2.4.5	amino acid metabolism.degradation.branched chain group.isoleucine									
**131**	D7T2N7	CBI24768.3	296084380	Late embryogenesis abundant protein Lea14-A	33	33.2	development.late embryogenesis abundant	10,7	4	64,51	**0,156**	0,01132	**0,238**	0,01544	**1,52**	**0,002**
**132**	A5C1K6	CAN66258.1	147835636	60S ribosomal protein L12	15	15.2	metal handling.binding, chelation and storage	33,1	4	61,45	**0,313**	0,01636	**0,271**	0,00359	**-1,15**	**0,031**
**133**	F6HXK4	CAN80223.1	147853720	Plasma membrane ATPase	34	34.1.2	transport.p- and v-ATPases.H+-exporting ATPase	5,2	4	61,35	**0,069**	0,00784	**0,159**	0,02276	**2,32**	**0,004**
**134**	A5AZ36	CBI27343.3	297738142	Glutathione S-transferase U25	26	26.9	misc.glutathione S transferases	16,8	4	60,26	**0,326**	0,02717	**0,114**	0,02776	**-2,85**	**0,000**
**135**	A5BIH7	CAN67965.1	147839059	Peptidase_S10 domain-containing protein	29	29.5.5	protein.degradation.serine protease	11,7	4	59,31	**0,060**	0,00715	**0,033**	0,00362	**-1,82**	**0,007**
**136**	F6HJB9	XP_002265072.1	225468829	Uncharacterized protein	35	35.1	not assigned.no ontology	14,2	4	59,21	**0,143**	0,02018	**0,062**	0,00706	**-2,30**	**0,004**
**137**	A5B878	XP_002271352.1	225453350	Nucleoside diphosphate kinase	23	23.4.10	nucleotide metabolism.phosphotransfer and pyrophosphatases.nucleoside diphosphate kinase	27	4	57,94	**0,383**	0,04390	**0,470**	0,03795	**1,23**	**0,166**
**138**	A5AGN5	XP_002280094.1	225446579	Ketol-acid reductoisomerase	13	13.1.4.1.2	amino acid metabolism.synthesis.branched chain group.common.ketol-acid reductoisomerase	8,6	4	57,86	**0,150**	0,02417	**0,120**	0,02086	**-1,24**	**0,379**
**139**	F6GT84	XP_002269118.1	225456761	Glutathione S-transferase U9	26	26.9	misc.glutathione S transferases	16,2	4	57,66	**0,328**	0,03785	**0,167**	0,02246	**-1,97**	**0,004**
**140**	F6HC76	CAN61687.1	147839972	Superoxide dismutase	21	21.6	redox.dismutases and catalases	15,9	4	54,9	**0,137**	0,03249	**0,116**	0,01333	**-1,17**	**0,577**
**141**	D7TWQ4	XP_002278444.1	225452472	Formate dehydrogenase, mitochondrial	25	25.10	C1-metabolism.formate dehydrogenase	12,2	4	54,38	**0,115**	0,05720	**0,238**	0,04420	**2,07**	**0,120**
**142**	D7TLL7	XP_002282090.1	225449350	Ras-related protein Rab2BV	30	30.5	signalling.G-proteins	16,5	4	54,21	**0,252**	0,02838	**0,192**	0,01157	**-1,32**	**0,075**
**143**	F6HP23	XP_002264455.1	225470723	Stilbene synthase 1	16	16.8.2.1	secondary metabolism.flavonoids.chalcones.naringenin-chalcone synthase	13	4	53,58	**0,264**	0,00770	**0,309**	0,02123	**1,17**	**0,072**
**144**	F6HI27	XP_002269441.1	225430650	Pyruvate dehydrogenase E1 component subunit beta-2, chloroplastic	1	1.3.8	PS.calvin cycle.transketolase	11,1	4	53,54	**0,116**	0,01743	**0,023**	0,01084	**-4,93**	**0,001**
					8	8.1.1.1	TCA / organic transformation.TCA.pyruvate DH.E1									
					11	11.1.31	lipid metabolism.FA synthesis and FA elongation.pyruvate DH									
**145**	D7T300	CBI24881.3	297736243	ATP synthase subunit O, mitochondrial	9	9.9	mitochondrial electron transport / ATP synthesis.F1-ATPase	18,7	4	53,23	**0,062**	0,01670	**0,041**	0,00907	**-1,53**	**0,287**
**146**	F6H710	XP_002279647.1	225432012	Galactokinase, putative	3	3.8.1	minor CHO metabolism.galactose.galactokinases	10,9	4	53,01	**0,033**	0,00605	**0,071**	0,01067	**2,17**	**0,011**
**147**	A5ANH8	CAN71501.1	147765656	Probable mitochondrial-processing peptidase subunit beta, mitochondrial	29	29.3.2	protein.targeting.mitochondria	9,7	4	51,38	**0,168**	0,02463	**0,147**	0,00524	**-1,14**	**0,425**
**148**	A5BV59	XP_002281279.1	225456096	Guanine nucleotide-binding protein subunit beta-like protein	27	27.3.99	RNA.regulation of transcription.unclassified	15,9	4	50,45	**0,071**	0,01238	**0,082**	0,01181	**1,14**	**0,564**
					29	29.4	protein.postranslational modification									
					30	30.5	signalling.G-proteins									
					33	33.99	development.unspecified									
**149**	E0CTI4	CBI21899.3	302143338	26S proteasome non-ATPase regulatory subunit 2 homolog	29	29.5.11.20	protein.degradation.ubiquitin.proteasom	7,4	4	49,17	**0,012**	0,00313	**0,036**	0,01014	**2,92**	**0,049**
**150**	A5CAF8	XP_002263796.1	225464995	Phosphoglycerate kinase	1	1.3.3	PS.calvin cycle.phosphoglycerate kinase	9,8	4	47,52	**0,252**	0,03794	**0,184**	0,02757	**-1,37**	**0,180**
					4	4.1.11	glycolysis.cytosolic branch.3-phosphoglycerate kinase (PGK)									
**151**	D7SL25	XP_002285368.1	225435395	Cinnamoyl-CoA reductase-like protein	16	16.8.3	secondary metabolism.flavonoids.dihydroflavonols	10,8	4	46,78	**0,049**	0,00125	**0,056**	0,00964	**1,13**	**0,539**
**152**	A5BYC0	XP_003632316.1	359479647	Proteasome subunit beta type	29	29.5.11.20	protein.degradation.ubiquitin.proteasom	21,4	4	46,17	**0,159**	0,00770	**0,145**	0,00321	**-1,10**	**0,128**
**153**	A7NY33	A7NY33.1	223635590	Peroxidase 4	26	26.12	misc.peroxidases	13,7	4	45,47	**0,000**	0,00000	**0,057**	0,01239	**New**	**0,001**
**154**	F6HFI0	XP_002284364.1	225424230	Carboxypeptidase	29	29.5.5	protein.degradation.serine protease	13,7	4	42,06	**0,026**	0,00399	**0,016**	0,00473	**-1,62**	**0,137**
**155**	O22519	NP_001268064.1	526117591	Chalcone synthase	16	16.8.2.1	secondary metabolism.flavonoids.chalcones.naringenin-chalcone synthase	12,3	4	40,07	**0,135**	0,05343	**0,009**	0,00519	**-14,75**	**0,041**

156	Q69D51	AAR06588.1	37992763	Beta-1,3-glucanase	26	26.4.1	misc.beta 1,3 glucan hydrolases.glucan endo-1,3-beta-glucosidase	14,8	3	68,16	0,000	0,00000	0,232	0,03237	New	0,000
157	F6GZY7	XP_002284973.1	225458701	Cysteine proteinase RD21A	29	29.5.3	protein.degradation.cysteine protease	9,6	3	68,06	0,399	0,01326	0,258	0,01266	-1,54	0,000
158	L7NSW9	NP_001267918.1	526118016	Plasma membrane 11 aquaporin	34	34.19.1	transport.major intrinsic proteins.PIP	14,6	3	58,94	0,425	0,00857	0,376	0,01737	-1,13	0,030
159	A0A172QNQ9	XP_003631658.1	359475330	Glycine-rich RNA-binding protein 2A	27	27.3.75	RNA.regulation of transcription.GRP	24,6	3	58,4	0,215	0,01100	0,268	0,01796	1,24	0,031
					27	27.4	RNA.RNA binding									
160	D7SW04	CBI21453.3	296082448	Bifunctional aspartate aminotransferase and glutamate/aspartate-prephenate aminotransferase isoform X2	13	13.1.1.2.1	amino acid metabolism.synthesis.central amino acid metabolism.aspartate.aspartate aminotransferase	11,7	3	57,61	0,314	0,04393	0,215	0,03280	-1,46	0,100
					13	13.2.6.2	amino acid metabolism.degradation.aromatic aa.tyrosine									
161	D7TRU0	CBI33216.3	297741856	Cinnamyl alcohol dehydrogenase 8	16	16.2.1.10	secondary metabolism.phenylpropanoids.lignin biosynthesis.CAD	9,6	3	57,16	0,233	0,00467	0,155	0,02466	-1,50	0,011
162	D7U2H1	XP_002275765.1	225437537	GTP-binding protein SAR1A	30	30.5	signalling.G-proteins	23,8	3	57,11	0,213	0,03510	0,281	0,03258	1,32	0,185
163	A5BQN6	XP_003631573.1	359475042	Peptidyl-prolyl cis-trans isomerase	26	26.8	misc.nitrilases, nitrile lyases, berberine bridge enzymes, reticuline oxidases, troponine reductases	20,3	3	55,7	0,198	0,09900	0,158	0,02610	-1,25	0,704
					31	31.3.1	cell.cycle.peptidylprolyl isomerase									
164	F6HLE8	XP_002281550.1	225441583	40S ribosomal protein S5	29	29.2.1.2.1.5	protein.synthesis.ribosomal protein.eukaryotic.40S subunit.S5	24,1	3	54,4	0,181	0,03673	0,148	0,03544	-1,23	0,528
165	D7TKM8	CBI31050.3	297740868	Putative germin-like protein 2-1	12	12.2.2	N-metabolism.ammonia metabolism.glutamine synthetase	20,9	3	53,38	0,250	0,04382	0,103	0,00752	-2,42	0,008
					20	20.2.99	stress.abiotic.unspecified									
					27	27.3.69	RNA.regulation of transcription.SET-domain transcriptional regulator family									
					30	30.3	signalling.calcium									
					34	34.12	transport.metal									
166	E0CR38	CBI18992.3	302141789	Proteasome subunit beta type	29	29.5.11.20	protein.degradation.ubiquitin.proteasom	24,5	3	53,26	0,173	0,02425	0,157	0,01676	-1,11	0,587
167	D7T8R2	CBI26883.3	297737682	MLP-like protein 43 isoform X2	20	20.1	stress.biotic	19,5	3	53,04	1,010	0,05024	0,937	0,01510	-1,08	0,194
					20	20.2.99	stress.abiotic.unspecified									
168	D7SY46	CBI22903.3	296083267	Dihydrolipoyl dehydrogenase 2, chloroplastic (8, 11, 21)	8	8.1.1.3	TCA / organic transformation.TCA.pyruvate DH.E3	7,7	3	51,52	0,105	0,00791	0,004	0,00402	-26,09	0,000
					11	11.1.31	lipid metabolism.FA synthesis and FA elongation.pyruvate DH									
					21	21.2.2	redox.ascorbate and glutathione.glutathione									
169	A5ARL2	NP_001268191.1	526118089	Peroxiredoxin	21	21.5	redox.peroxiredoxin	20,9	3	51,23	0,207	0,02200	0,233	0,02063	1,13	0,399
170	F6HZD8	CBI37013.3	297744043	Short-chain dehydrogenase reductase 3b-like	26	26.22	misc.short chain dehydrogenase/reductase (SDR)	10,2	3	50,34	0,238	0,02748	0,000	0,00000	d.	0,000
171	D7TR81	CBI33004.3	297741775	Pyrophosphate--fructose 6-phosphate 1-phosphotransferase subunit beta	4	4.2.5	glycolysis.plastid branch.pyrophosphate-fructose-6-P phosphotransferase	6,5	3	49,89	0,037	0,00690	0,026	0,00100	-1,40	0,159
172	A0A0D5W623	AJZ72652.1	782909551	Aquaporin PIP21	34	34.19.1	transport.major intrinsic proteins.PIP	21,5	3	49,68	0,125	0,03483	0,125	0,03275	-1,00	0,997
173	A5C5V3	CAN81862.1	147860330	Dihydroceramide fatty acyl 2-hydroxylase FAH1	11	11.2.2	lipid metabolism.FA desaturation.a hydroxylase	17,3	3	48,23	0,103	0,01334	0,003	0,00318	-32,25	0,000
					11	11.8.1	lipid metabolism.exotics (steroids, squalene etc).sphingolipids									
174	A5B0T9	XP_002273982.1	225431840	Major allergen Pru ar 1	20	20.1.7	stress.biotic.PR-proteins	22,6	3	47,97	0,066	0,02500	0,068	0,00814	1,02	0,960
					27	27.1.19	RNA.processing.ribonucleases									
					34	34.16	transport.ABC transporters and multidrug resistance systems									
175	F6GX20	XP_002277981.1	225430941	4-hydroxy-4-methyl-2-oxoglutarate aldolase (25)	25	25	C1-metabolism	15,1	3	47,53	0,099	0,01403	0,150	0,01108	1,52	0,017
176	D7U318	XP_002272576.3	1105495218	Dolichyl-diphosphooligosaccharide--protein glycosyltransferase subunit 2	33	33.99	development.unspecified	5,8	3	47,35	0,202	0,02968	0,356	0,09772	1,76	0,162
177	D7U5C1	NP_001268084.1	526117669	Ripening-related protein-like	13	13.1.3.4	amino acid metabolism.synthesis.aspartate family.methionine	23,3	3	46,27	0,344	0,09952	0,389	0,10582	1,13	0,763
178	F6HE11	XP_002263180.1	225432110	Dihydrolipoyl dehydrogenase	8	8.1.1.3	TCA / organic transformation.TCA.pyruvate DH.E3	7,5	3	46,22	0,071	0,00751	0,063	0,00617	-1,13	0,407
					21	21.2.2	redox.ascorbate and glutathione.glutathione									
179	C0KY93	ACN38270.1	224038270	Leucoanthocyanidin dioxygenase	1	1.2.6	PS.photorespiration.hydroxypyruvate reductase	11,5	3	45,49	0,190	0,02770	0,165	0,02638	-1,15	0,533
					7	7.1.3	OPP.oxidative PP.6-phosphogluconate dehydrogenase									
					13	13.2.5.1	amino acid metabolism.degradation.serine-glycine-cysteine group.serine									
					13	13.2.5.2	amino acid metabolism.degradation.serine-glycine-cysteine group.glycine									
					16	16.8.1.1	secondary metabolism.flavonoids.anthocyanins.leucocyanidin dioxygenase									
					17	17.5.1	hormone metabolism.ethylene.synthesis-degradation									
					26	26.1	misc.misc2									
180	F6HXL1	CBI35797.3	297742930	Proteasome subunit alpha type	29	29.5.11.20	protein.degradation.ubiquitin.proteasom	11,6	3	45,42	0,081	0,00172	0,096	0,01243	1,18	0,272
181	F6HZ27	XP_002262842.1	225437288	Glutathione S-transferase	26	26.9	misc.glutathione S transferases	13	3	45,35	0,483	0,01125	0,450	0,00982	-1,07	0,055
182	D7STF0	CBI20064.3	296081541	3-hydroxyacyl-[acyl-carrier-protein] dehydratase FabZ	11	11.1.5	lipid metabolism.FA synthesis and FA elongation.beta hydroxyacyl ACP dehydratase	15,3	3	45,28	0,184	0,00885	0,057	0,00973	-3,22	0,000
183	D7U7S6	XP_002279266.1	225453909	Proteasome subunit beta type	17	17.6.3	hormone metabolism.gibberelin.induced-regulated-responsive-activated	23,7	3	44,81	0,169	0,03397	0,151	0,02165	-1,12	0,665
					26	26.10	misc.cytochrome P450									
					29	29.5.11.20	protein.degradation.ubiquitin.proteasom									
184	A5C6H7	CAN68704.1	147800323	Sucrose synthase	2	2.2.1.5	major CHO metabolism.degradation.sucrose.Susy	3,8	3	44,35	0,001	0,00082	0,123	0,00824	150,46	0,000
185	F6I1P0	XP_002264210.1	225425166	Pyruvate dehydrogenase E1 component subunit beta	8	8.1.1.1	TCA / organic transformation.TCA.pyruvate DH.E1	10,3	3	43,51	0,023	0,01008	0,081	0,01775	3,49	0,017
186	D7SHN2	CBI14992	297733745	heme-binding protein 2-like	19	19.99	tetrapyrrole synthesis.unspecified	10,4	3	43,13	0,153	0,01153	0,058	0,01625	-2,63	0,001
					29	29.5.11.5	protein.degradation.ubiquitin.ubiquitin protease									
187	F6I6W5	XP_002276269.1	225457674	Pyrophosphate--fructose 6-phosphate 1-phosphotransferase subunit alpha	4	4.1.5	glycolysis.cytosolic branch.pyrophosphate-fructose-6-P phosphotransferase	4,7	3	43	0,110	0,00255	0,106	0,00300	-1,04	0,258
188	D7TVI4	CBI34509	297742360	3-oxoacyl-[acyl-carrier-protein] synthase I, chloroplastic	11	11.1.3	lipid metabolism.FA synthesis and FA elongation.ketoacyl ACP synthase	9,6	3	42,54	0,037	0,00986	0,000	0,00000	d.	0,004
189	A5BE54	XP_002282471.1	225452700	Mitochondrial outer membrane protein porin 3	18	18.5.2.1	Co-factor and vitamine metabolism.folate and vitamine K.vitamine K.isochorismate synthase	8,3	3	40,71	0,191	0,01399	0,171	0,01142	-1,12	0,294
					34	34.2	transport.sugars									
					34	34.20	transport.porins									
190	D7T8G2	CBI26783.3	297737582	Purple acid phosphatase	26	26.13	misc.acid and other phosphatases	10,4	3	40,32	0,102	0,02368	0,003	0,00267	-38,33	0,002
191	A5ATW2	CAN60563.1	147837952	Bifunctional 3-dehydroquinate dehydratase/shikimate dehydrogenase	13	13.1.6.1.10	amino acid metabolism.synthesis.aromatic aa.chorismate.3-dehydroquinate dehydratase/shikimate dehydrogenase	3,9	3	40,28	0,053	0,00914	0,030	0,00115	-1,79	0,029
192	F6HDM0	XP_002280658.1	225432548	Stem-specific protein TSJT1	15	15	metal handling	19,9	3	40,26	0,033	0,01273	0,021	0,00635	-1,57	0,421
					17	17.2.3	hormone metabolism.auxin.induced-regulated-responsive-activated									
193	F6I5L8	XP_002265881.1	225452186	Prohibitin-3, mitochondrial	9	9.1.1	mitochondrial electron transport / ATP synthesis.NADH-DH (type I).complex I	15,5	3	40	0,115	0,02364	0,082	0,00983	-1,40	0,232
					31	31.2	cell.division									
194	F6H684	XP_002269439.1	225429053	Serine carboxypeptidase-like 7 isoform X1	29	29.5.5	protein.degradation.serine protease	5,9	3	39,91	0,048	0,00798	0,049	0,00881	1,02	0,941

195	F6H1D7	CBI19568.3	302142365	Carboxypeptidase (29)	29	29.5.5	protein.degradation.serine protease	7,1	3	38,84	0,245	0,03963	0,118	0,03174	-2,08	0,031
196	F6H727	XP_002277653.1	225431940	Universal stress protein PHOS32 isoform X1	20	20.2.2	stress.abiotic.cold	20	3	38,55	0,096	0,02275	0,114	0,01594	1,19	0,527
197	D7T9I6	XP_002278126.1	225423722	Proteasome subunit alpha type-2-B	29	29.5.11.20	protein.degradation.ubiquitin.proteasom	14,8	3	37,82	0,051	0,00909	0,068	0,01003	1,35	0,224
198	E0CP35	XP_002285252.1	225458816	Adenine phosphoribosyltransferase 1, chloroplastic isoform X1	23	23.3.1.1	nucleotide metabolism.salvage.phosphoribosyltransferases.adenine phosphoribosyltransferase (APRT)	29,6	3	34,69	0,043	0,01087	0,041	0,00919	-1,05	0,883
199	D7SGX1	CBI15651.3	297734404	Elongation factor 1-delta 1	29	29.2.4	protein.synthesis.elongation	12,2	3	34,55	0,165	0,01370	0,128	0,02529	-1,29	0,228
200	F6HKE8	CBI30191.3	297740009	Serine carboxypeptidase	29	29.5.5	protein.degradation.serine protease	4,2	3	34,27	0,037	0,01926	0,030	0,01645	-1,20	0,816
201	Q0MYQ7	NP_001267914.1	526118000	Germin-like protein 2	15	15.2	metal handling.binding, chelation and storage	13,3	3	34,07	0,003	0,00283	0,126	0,00836	44,61	0,000
					20	20.2.99	stress.abiotic.unspecified									
202	D7T9L8	CBI27189.3	297737988	Coatomer subunit delta	31	31.4	cell.vesicle transport	5,7	3	32,54	0,077	0,01756	0,035	0,00410	-2,21	0,043
203	E0CQ39	XP_002282774.1	225458305	Glucose-6-phosphate isomerase	4	4.1.3	glycolysis.cytosolic branch.glucose-6-phosphate isomerase	7,3	3	31,31	0,032	0,01453	0,023	0,01398	-1,43	0,645
204	A5AY42	XP_002277452.1	225449132	ATP synthase subunit d, mitochondrial	9	9.9	mitochondrial electron transport / ATP synthesis.F1-ATPase	15,4	3	30,75	0,050	0,01805	0,029	0,01823	-1,73	0,429
205	A5B620	CAN80832.1	147858936	Methyltransferase	17	17.3.1.2.1	hormone metabolism.brassinosteroid.synthesis-degradation.sterols.SMT1	6,6	3	29,23	0,000	0,00000	0,019	0,01056	New	0,106
206	F6GUF3	XP_002285652.2	359478431	Peroxidase 53	26	26.12	misc.peroxidases	8,2	3	29,12	0,000	0,00000	0,033	0,02016	New	0,133
207	A3QRC1	NP_001267900.1	526117952	Allene oxide cyclase 2, chloroplastic	17	17.7.1.4	hormone metabolism.jasmonate.synthesis-degradation.allene oxidase cyclase	12,2	3	28,76	0,127	0,00379	0,062	0,01957	-2,06	0,008
					20	20.2.3	stress.abiotic.drought/salt									
208	D7SYM2	XP_010664545.1	731381423	Uncharacterized protein	26	26.8	misc.nitrilases, nitrile lyases, berberine bridge enzymes, reticuline oxidases, troponine reductases	12,5	2	42,95	0,160	0,01371	0,209	0,02172	1,31	0,085
209	F6GU75	XP_002276130.2	359478860	26S proteasome regulatory subunit 6B homolog	29	29.5.11.20	protein.degradation.ubiquitin.proteasom	7,4	2	42,71	0,155	0,01461	0,143	0,02495	-1,08	0,698
210	F6HS56	XP_002285645.1	225433414	Potassium channel beta, putative	17	17.2.3	hormone metabolism.auxin.induced-regulated-responsive-activated	10	2	41,78	0,103	0,00780	0,065	0,00924	-1,59	0,010
					34	34.15	transport.potassium									
211	D7T4I1	CBI25413.3	297736542	Biotin carboxyl carrier protein of acetyl-CoA carboxylase 2, chloroplastic	11	11.1.1	lipid metabolism.FA synthesis and FA elongation.acetyl CoA carboxylation	15,2	2	41,59	0,177	0,02915	0,041	0,01137	-4,35	0,001
212	D7TP00	XP_002263386.1	225436699	Glutathione S-transferase F13	26	26.9	misc.glutathione S transferases	12,9	2	41,52	0,467	0,02834	0,502	0,03495	1,08	0,450
213	F6HSU5	XP_002284278.1	225438962	Peroxidase	26	26.12	misc.peroxidases	9,3	2	40,03	0,000	0,00000	0,009	0,00613	New	0,174
214	F6HZ19	CBI36810.3	296088365	Germin-like protein subfamily 1 member 17	12	12.2.2	N-metabolism.ammonia metabolism.glutamine synthetase	12,2	2	39,7	0,064	0,00545	0,158	0,00351	2,46	0,000
					20	20.2.99	stress.abiotic.unspecified									
					27	27.3.69	RNA.regulation of transcription.SET-domain transcriptional regulator family									
					34	34.12	transport.metal									
215	D7U0Q6	CBI36202.3	297743335	Probable plastid-lipid-associated protein 1, chloroplastic	16	16.5.1.1.1.11	secondary metabolism.sulfur-containing.glucosinolates.synthesis.aliphatic.glucosinolate 2-oxoglutarate-dependent dioxygenase (AOP)	8,5	2	39,08	0,126	0,01920	0,071	0,01466	-1,78	0,046
					17	17.5.1	hormone metabolism.ethylene.synthesis-degradation									
					17	17.6.1.11	hormone metabolism.gibberelin.synthesis-degradation.GA20 oxidase									
					26	26.14	misc.oxygenases									
216	A5BPW8	CAN63722.1	147811922	NAD(P)H dehydrogenaseFQR1-like	11	11.8	lipid metabolism.exotics (steroids, squalene etc)	17,9	2	36,67	0,088	0,02085	0,074	0,02321	-1,19	0,667
217	D7SPF1	CBI17530.3	297735168	Succinate dehydrogenase [ubiquinone] flavoprotein subunit, mitochondrial	8	8.1.7	TCA / organic transformation.TCA.succinate dehydrogenase	4,1	2	36,27	0,083	0,02565	0,141	0,01619	1,70	0,086
218	A5ARE0	CAN80347.1	147858030	Glutelin type-A 1-like	28	28.2	DNA.repair	8,9	2	35,32	0,015	0,00677	0,091	0,01583	6,08	0,001
					33	33.1	development.storage proteins									
219	A5B729	CAN78723.1	147860147	Uncharacterized protein	35	35.2	not assigned.unknown	9	2	34,81	0,105	0,02080	0,000	0,00000	d.	0,001
220	D7TRJ7	XP_010646198.1	731439308	Fumarylacetoacetase	13	13.2.6.2	amino acid metabolism.degradation.aromatic aa.tyrosine	6,2	2	34,69	0,136	0,01782	0,147	0,01342	1,08	0,634
221	F6I1D6	XP_002265749.2	359489134	Non-specific phospholipase C3 isoform X1	11	11.9.3.5	lipid metabolism.lipid degradation.lysophospholipases.phosphoinositide phospholipase C	4,6	2	34,35	0,056	0,00875	0,056	0,00794	1,01	0,966
222	F6HTY5	XP_002268436.1	225451122	Superoxide dismutase [Cu-Zn]	21	21.6	redox.dismutases and catalases	17,2	2	34,15	0,068	0,00243	0,143	0,01362	2,11	0,000
223	A5AXT8	CAN65024.1	147810325	Pentatricopeptide repeat-containing protein At5g66520-like	26	26.11	misc.alcohol dehydrogenases	5,4	2	33,82	0,108	0,04545	0,000	0,00000	d.	0,039
					27	27.1	RNA.processing									
224	F6HX49	CBI36206.3	297743339	Gibberellin 20 oxidase 1	16	16.5.1.1.1.11	secondary metabolism.sulfur-containing.glucosinolates.synthesis.aliphatic.glucosinolate 2-oxoglutarate-dependent dioxygenase (AOP)	7,9	2	33,67	0,125	0,01278	0,052	0,01050	-2,39	0,001
					17	17.5.1	hormone metabolism.ethylene.synthesis-degradation									
					17	17.6.1	hormone metabolism.gibberelin.synthesis-degradation									
					17	17.6.1.11	hormone metabolism.gibberelin.synthesis-degradation.GA20 oxidase									
					26	26.14	misc.oxygenases									
225	D7TW03	XP_002272936.1	225426405	Glutathione peroxidase 8 isoform X1	21	21.2.2	redox.ascorbate and glutathione.glutathione	14,7	2	32,86	0,019	0,01035	0,031	0,01003	1,67	0,404
226	D7SUQ2	CBI21001.3	296081996	GTP-binding nuclear protein	30	30.5	signalling.G-proteins	6,8	2	32,61	0,100	0,00527	0,102	0,00365	1,02	0,791
227	F6H2P8	CBI20205.3	302142910	Protein DJ-1 homolog B	18	18.2	Co-factor and vitamine metabolism.thiamine	5,3	2	31,85	0,009	0,00552	0,059	0,00690	6,81	0,000
228	D7TZC8	XP_002271007.4	1105501277	Uncharacterized protein LOC100247307	35	35.2	not assigned.unknown	13,2	2	31,85	0,074	0,02501	0,210	0,06322	2,84	0,073
229	E0CSF7	XP_019072855.1	1104683316	D-amino-acid transaminase, chloroplastic isoform X1	13	13.1	amino acid metabolism.synthesis	4,5	2	31,28	0,031	0,00105	0,057	0,01234	1,83	0,064
230	D7U564	XP_002278162.1	225428005	Proteasome subunit alpha type	17	17.8.1	hormone metabolism.salicylic acid.synthesis-degradation	10	2	30,8	0,080	0,00906	0,078	0,01190	-1,03	0,897
					29	29.4	protein.postranslational modification									
					29	29.5.11.20	protein.degradation.ubiquitin.proteasom									
					33	33.99	development.unspecified									
231	A5ACR7	XP_002282568.1	225452712	V-type proton ATPase subunit	34	34.1.1	transport.p- and v-ATPases.H+-transporting two-sector ATPase	7,1	2	30,76	0,039	0,00430	0,041	0,00785	1,04	0,852
232	F6HXK8	XP_002265171.1	225441977	Pectinesterase	10	10.8.1	cell wall.pectinesterases.PME	3,1	2	30,73	0,093	0,01017	0,122	0,02654	1,31	0,339
233	Q1KTA7	ABF06881.1	93352786	Stilbene synthase	16	16.8.2.1	secondary metabolism.flavonoids.chalcones.naringenin-chalcone synthase	5,6	2	30,29	0,157	0,01897	0,162	0,02054	1,03	0,875
234	F6HZ29	XP_010652165.1	731395413	Bifunctional protein FolD 2	20	20.1	stress.biotic	10,4	2	29,51	0,027	0,00413	0,033	0,00327	1,21	0,316
					25	25.5	C1-metabolism.methylenetetrahydrofolate dehydrogenase and methenyltetrahydrofolate cyclohydrolase									
235	F6H8F3	XP_003635669.1	359497849	Phosphoserine aminotransferase	13	13.1.5.1.2	amino acid metabolism.synthesis.serine-glycine-cysteine group.serine.phosphoserine aminotransferase	7,4	2	29,11	0,045	0,02514	0,022	0,00670	-1,98	0,416
					27	27.3.22	RNA.regulation of transcription.homeobox transcription factor family (HB)									
236	A5C2G6	XP_002277763.1	225434588	Peptidyl-prolyl cis-trans isomerase	31	31.3.1	cell.cycle.peptidylprolyl isomerase	13,7	2	28,92	0,049	0,00898	0,051	0,00581	1,03	0,894
237	F6I5I7	XP_002272730.1	225436253	Methylenetetrahydrofolate reductase	25	25.6	C1-metabolism.methylenetetrahydrofolate reductase	4,7	2	28,83	0,027	0,00910	0,066	0,02360	2,45	0,152
238	F6H0J2	XP_002285876.2	359492398	DPP6 N-terminal domain-like protein	35	35.1	not assigned.no ontology	3,3	2	27,7	0,034	0,00206	0,013	0,00549	-2,53	0,006
239	F6HIC8	CBI28822.3	297739171	Dienelactone hydrolase	26	26.1	misc.misc2	6,5	2	27,54	0,022	0,01003	0,018	0,00707	-1,19	0,780
240	F6HTU0	XP_002276988.1	225451257	26S proteasome non-ATPase regulatory subunit 11 homolog	29	29.5.11.20	protein.degradation.ubiquitin.proteasom	6,3	2	27,26	0,044	0,00643	0,041	0,01052	-1,07	0,824

**241**	D7TDF1	XP_003631258.1	359473184	Carbonic anhydrase	8	8.3	TCA / organic transformation.carbonic anhydrases	10,9	2	27,17	**0,068**	0,01568	**0,039**	0,00916	**-1,73**	**0,145**
					16	16.99	secondary metabolism.unspecified									
**242**	F6H120	XP_002283889.2	359492937	Aspartyl protease AED3	27	27.3.99	RNA.regulation of transcription.unclassified	9,1	2	27,03	**0,011**	0,00368	**0,009**	0,00588	**-1,18**	**0,816**
					29	29.5.4	protein.degradation.aspartate protease									
**243**	E0CP87	CBI19544.3	302142341	Patatin	33	33.1	development.storage proteins	9,7	2	26,99	**0,029**	0,01171	**0,023**	0,01362	**-1,26**	**0,751**
**244**	D7UE33	CBI40998.3	296090614	PLAT domain-containing protein 3-like	20	20.2	stress.abiotic	10,8	2	26,9	**0,159**	0,00181	**0,161**	0,00521	**1,01**	**0,698**
**245**	D7TUE8	CBI34123.3	296087534	Glycosyltransferase	26	26.2	misc.UDP glucosyl and glucoronyl transferases	7	2	26,89	**0,103**	0,01706	**0,072**	0,01614	**-1,41**	**0,230**
**246**	F6H9B5	XP_002281493.1	225461052	Glucose-6-phosphate/phosphate translocator 1, chloroplastic	2	2.1.2.5	major CHO metabolism.synthesis.starch.transporter	5,6	2	26,83	**0,007**	0,00448	**0,031**	0,00549	**4,78**	**0,006**
					2	2.2.2.6	major CHO metabolism.degradation.starch.transporter									
					34	34.8	transport.metabolite transporters at the envelope membrane									
**247**	A5B894	XP_002284591.1	225458573	Succinate dehydrogenase subunit 5, mitochondrial	20	20.2.1	stress.abiotic.heat	19	2	26,71	**0,011**	0,00674	**0,014**	0,00465	**1,29**	**0,715**
**248**	F6I455	XP_002278756.2	359493457	Probable elongation factor 1-gamma 2	29	29.2.4	protein.synthesis.elongation	8,2	2	26,42	**0,057**	0,02964	**0,138**	0,04889	**2,45**	**0,183**
**249**	F6HL77	CBI30508.3	297740326	Tropinone reductase homolog At1g07440	26	26.8	misc.nitrilases, nitrile lyases, berberine bridge enzymes, reticuline oxidases, troponine reductases	10,6	2	26,4	**0,021**	0,00788	**0,001**	0,00071	**-29,52**	**0,028**
**250**	S5FNE7	XP_002272119.1	225432270	Protein SRG1	16	16.8.4	secondary metabolism.flavonoids.flavonols	10,4	2	25,85	**0,098**	0,01450	**0,000**	0,00000	**d.**	**0,000**
					17	17.5.1	hormone metabolism.ethylene.synthesis-degradation									
**251**	A5C997	XP_002262780.1	225470692	Membrane steroid-binding protein 2	21	21.2	redox.ascorbate and glutathione	13,9	2	25,79	**0,074**	0,00369	**0,074**	0,00609	**-1,00**	**0,989**
**252**	F6I407	XP_002267428.1	225460394	Patellin-3	29	29.3.4.99	protein.targeting.secretory pathway.unspecified	3,1	2	25,69	**0,061**	0,00444	**0,050**	0,00615	**-1,21**	**0,196**
					34	34.99	transport.misc									
**253**	E0CU14	XP_002283209.1	225446791	Glutathione S-transferase F9	11	11.1.8	lipid metabolism.FA synthesis and FA elongation.acyl CoA ligase	21,7	2	25,03	**0,001**	0,00080	**0,009**	0,00371	**11,03**	**0,061**
					11	11.1.8	lipid metabolism.FA synthesis and FA elongation.acyl CoA ligase									
					26	26.9	misc.glutathione S transferases									
**254**	D7SNS5	XP_002267004.1	225433514	Selenium-binding protein 1	15	15	metal handling	5,7	2	25	**0,030**	0,03000	**0,053**	0,02347	**1,78**	**0,554**
**255**	Q38JC9	NP_001268165.1	526117984	Temperature-induced lipocalin	34	34.99	transport.misc	12,4	2	24,77	**0,004**	0,00170	**0,011**	0,00247	**2,49**	**0,057**
**256**	F6HHQ2	XP_002267128.1	225447524	Nitrile-specifier protein 5	16	16.5.1.3.2	secondary metabolism.sulfur-containing.glucosinolates.degradation.nitrilespecifier protein	7	2	24,62	**0,000**	0,00000	**0,067**	0,01364	**New**	**0,001**
					17	17.7.3	hormone metabolism.jasmonate.induced-regulated-responsive-activated									
					26	26.16	misc.myrosinases-lectin-jacalin									
**257**	F6GT83	CBI15230.3	297733983	glutathione S-transferase	26	26.9	misc.glutathione S transferases	9	2	24,52	**0,130**	0,01968	**0,090**	0,01444	**-1,44**	**0,138**
**258**	F6GWY9	XP_002273030.1	225430832	NAD(P)H dehydrogenase (quinone) FQR1-like 2	11	11.8	lipid metabolism.exotics (steroids, squalene etc)	16,7	2	24,16	**0,039**	0,01998	**0,021**	0,02146	**-1,84**	**0,554**
					26	26.8	misc.nitrilases, nitrile lyases, berberine bridge enzymes, reticuline oxidases, troponine reductases									
					27	27.3.99	RNA.regulation of transcription.unclassified									
**259**	D7U2N2	CBI36998.3	297744028	PII-like protein	30	30.1.1	signalling.in sugar and nutrient physiology.misc	12,6	2	23,61	**0,054**	0,00329	**0,041**	0,00214	**-1,32**	**0,008**
**260**	D7U851	CBI38733.3	296089030	Prohibitin-1, mitochondrial	9	9.1.1	mitochondrial electron transport / ATP synthesis.NADH-DH (type I).complex I	6,6	2	23,58	**0,042**	0,01290	**0,031**	0,00368	**-1,35**	**0,439**
					31	31.3	cell.cycle									
**261**	F6I080	CAN74858.1	147811874	Bifunctional nitrilase/nitrile hydratase NIT4B-like	16	16.5.1.3.3	secondary metabolism.sulfur-containing.glucosinolates.degradation.nitrilase	14,1	2	23,53	**0,000**	0,00000	**0,028**	0,00969	**New**	**0,015**
					26	26.8	misc.nitrilases, nitrile lyases, berberine bridge enzymes, reticuline oxidases, troponine reductases									
**262**	A5B0N6	XP_002264813.1	225462628	Glutathione S-transferase parA	26	26.9	misc.glutathione S transferases	6,7	2	23,17	**0,092**	0,02627	**0,048**	0,02100	**-1,94**	**0,212**
**263**	F6HLB4	NP_001267906.1	526117970	Glutamate dehydrogenase	12	12.3.1	N-metabolism.N-degradation.glutamate dehydrogenase	3,9	2	22,96	**0,091**	0,05031	**0,007**	0,00428	**-13,46**	**0,126**
**264**	G9FKG6	AEW31187.1	363805186	Glycosyltransferase	17	17.2.1	hormone metabolism.auxin.synthesis-degradation	4,8	2	22,91	**0,065**	0,04315	**0,032**	0,00930	**-2,07**	**0,461**
					26	26.2	misc.UDP glucosyl and glucoronyl transferases									
**265**	F6I106	XP_002278281.1	225428009	L-ascorbate peroxidase 3, peroxisomal	21	21.2.1	redox.ascorbate and glutathione.ascorbate	6,7	2	22,82	**0,063**	0,00689	**0,059**	0,00311	**-1,08**	**0,569**
**266**	D7SIX7	CBI15438.3	297734191	Serine/threonine-protein phosphatase 2A 65 kDa regulatory subunit A beta isoform	29	29.4	protein.postranslational modification	5,9	2	22,65	**0,006**	0,00634	**0,036**	0,00724	**5,60**	**0,013**
**267**	A5BR79	XP_002285103.1	225428869	Glycosyltransferase	26	26.2	misc.UDP glucosyl and glucoronyl transferases	4,8	2	22,19	**0,099**	0,00994	**0,077**	0,01576	**-1,29**	**0,264**
**268**	F6HL42	XP_002272909.1	225440478	NADPH-dependent aldo-keto reductase, chloroplastic	3	3.5	minor CHO metabolism.others	5,7	2	22,17	**0,038**	0,02106	**0,004**	0,00417	**-9,16**	**0,144**
**269**	F6HGK8	XP_002283629.2	359484336	3-hydroxyisobutyrate dehydrogenase-like 1, mitochondrial	7	7.1.3	OPP.oxidative PP.6-phosphogluconate dehydrogenase	12,4	2	22,09	**0,019**	0,01212	**0,003**	0,00274	**-6,86**	**0,225**
**270**	A5C8F3	CAN75406.1	147828269	Ras-related protein Rab7	30	30.5	signalling.G-proteins	10,1	2	21,93	**0,034**	0,01670	**0,004**	0,00407	**-8,39**	**0,111**
**271**	F6H0K6	XP_002285865.1	225459591	L-ascorbate peroxidase 6, chloroplastic isoform X1	21	21.2.1	redox.ascorbate and glutathione.ascorbate	5,2	2	21,84	**0,009**	0,00573	**0,020**	0,01622	**2,25**	**0,531**
**272**	F6HBY3	XP_002271902.2	731403637	Nudix hydrolase 3	23	23.3.3	nucleotide metabolism.salvage.NUDIX hydrolases	2,6	2	21,81	**0,017**	0,00766	**0,001**	0,00078	**-21,82**	**0,061**
**273**	D7T0M7	XP_002266488.1	225445670	Proteasome subunit beta type	29	29.5.11.20	protein.degradation.ubiquitin.proteasom	8,1	2	21,8	**0,013**	0,00614	**0,010**	0,00621	**-1,36**	**0,695**
**274**	A5BV56	XP_010652106.1	731395250	Elongation factor Tu	29	29.2.4	protein.synthesis.elongation	9,5	2	21,71	**0,008**	0,00821	**0,006**	0,00450	**-1,27**	**0,855**
**275**	A5C9Z5	CAN66540.1	147800093	V-type proton ATPase subunit E3	29	29.2.3	protein.synthesis.initiation	7,1	2	21,58	**0,031**	0,01162	**0,031**	0,01143	**-1,01**	**0,990**
**276**	D7SJF5	CBI15781.3	297745725	CBS domain-containing protein CBSX3, mitochondrial (Fragment)	35	35.1	not assigned.no ontology	8	2	21,43	**0,080**	0,02049	**0,100**	0,00345	**1,25**	**0,354**
**277**	F6H2W4	XP_002283440.1	225429870	Aspartate--tRNA ligase 2, cytoplasmic	29	29.1.12	protein.aa activation.aspartate-tRNA ligase	3,4	2	20,31	**0,000**	0,00000	**0,010**	0,00266	**New**	**0,003**
**278**	D7SGV3	XP_002283508.1	225456268	Adenylate kinase 4	23	23.4.1	nucleotide metabolism.phosphotransfer and pyrophosphatases.adenylate kinase	13	2	19,99	**0,012**	0,00390	**0,010**	0,00255	**-1,19**	**0,695**
**279**	D7TNE5	CBI32018.3	296086429	Hypersensitive-induced response protein 1-like isoform X1	20	20.1.99	stress.biotic.misc	4,5	2	19,84	**0,107**	0,05160	**0,162**	0,03932	**1,52**	**0,412**
**280**	D7SJV3	CBI15929.3	297745873	Clathrin heavy chain 2	31	31.4	cell.vesicle transport	2	2	18,17	**0,000**	0,00000	**0,006**	0,00308	**New**	**0,086**

SUPPLEMENTARY DATA 1

Root proteomic analysis of two grapevine rootstock genotypes showing different susceptibility to salt stress.

Bhakti Prinsi, Osvaldo Failla, Attilio Scienza, Luca Espen
Department of Agricultural and Environmental Sciences—Production, Landscape, Agroenergy (DiSAA), Università degli Studi di Milano, Via Celoria 2, 20133 Milano, Italy

Table S2. Protein quantification by LC-ESI-MS/MS in the M4 root proteome. Protein quantification was obtained by summing the spectrum intensity (SI) of all the identified peptides in the protein. Protein abundance was normalized as the percentage with respect to the abundance of all validated proteins in the sample [%(SI)]. The results are the mean of three biological samples (n=3).

#	Accession SwisProt	Accession NCBI	Accession GI	Name	BINCODE		Function	amino acid coverage %	# unique identified peptide	Protein MS/MS Search Score	Control Mean %(SI)	Control SE	WS Mean %(SI)	WS SE	Fold changes (ΔSS/C)	t-test
1	F6HNX5	XP_002283532.2	359486799	Heat shock cognate 70 kDa protein 2	20	20.2.1	stress.abiotic.heat	47,6	21	437,5	3,755	0,23536	3,507	0,12976	-1,07	0,378
					29	29.6	protein.folding									
2	F6GSG7	XP_002263145.2	359491599	Glyceraldehyde-3-phosphate dehydrogenase	4	4.1.8	glycolysis.cytosolic branch.glyceraldehyde 3-phosphate dehydrogenase (GAP-DH)	68,5	20	319,34	3,554	0,09428	3,964	0,10734	1,12	0,017
3	F6GTT2	XP_002280824.1	225456079	ATP synthase subunit beta, mitochondrial	9	9.9	mitochondrial electron transport / ATP synthesis.F1-ATPase	57,4	19	375,31	4,702	0,19710	4,497	0,17595	-1,05	0,455
4	F6HMN8	XP_002276438.2	225439223	5-methyltetrahydropteroyltriglutamate--homocysteine methyltransferase	13	13.1.3.4.3	amino acid metabolism.synthesis.aspartate family.methionine.methionine synthase	26,7	18	294,6	1,277	0,10493	1,549	0,08216	1,21	0,069
5	A5B118	CAN71905.1	147781269	Fructose-bisphosphate aldolase	13	1.3.6	PS.calvin cycle.aldolase	47,7	15	282	1,236	0,06885	1,480	0,05767	1,20	0,022
					4	4.1.10	glycolysis.cytosolic branch.aldolase									
6	B6VJY3	YP_002608395.1	224365668	ATP synthase subunit alpha	9	9.9	mitochondrial electron transport / ATP synthesis.F1-ATPase	28,4	15	225,82	1,985	0,11044	1,719	0,06843	-1,15	0,068
7	A5BZF5	XP_010664458.1	731428825	Tubulin alpha-4 chain	31	31.1	cell.organisation	45,4	14	252,49	2,016	0,14033	1,789	0,08469	-1,13	0,195
8	F6I0I5	XP_002282516.1	225431585	Actin-8	31	31.1	cell.organisation	33,2	13	218,69	2,858	0,15484	2,010	0,06951	-1,42	0,001
9	A5C3B4	CAN81774.1	147856322	14-3-3-like protein GF14 psi	30	30.7	signalling.14-3-3 proteins	48,4	13	208,94	2,065	0,15292	1,849	0,06003	-1,12	0,218
10	D7SS06	CBI18438.3	297735751	V-type proton ATPase catalytic subunit A	34	34.1.1	transport.p- and v-ATPases.H+-transporting two-sector ATPase	27,2	13	207,98	0,581	0,04574	0,419	0,03584	-1,39	0,006
11	A5ATG8	XP_002283056.1	225440131	Tubulin beta chain	31	31.1	cell.organisation	35,1	13	204,44	1,214	0,12231	1,062	0,06455	-1,14	0,298
12	A5BTZ8	CAN81470.1	147861246	Annexin	31	31.1	cell.organisation	40,1	12	212,92	1,115	0,04758	1,592	0,02530	1,43	0,000
13	A5B8T3	CAN67659.1	147820522	Fructokinase	2	2.2.1.1	major CHO metabolism.degradation.sucrose.fructokinase	41,5	12	212,86	1,116	0,05408	0,601	0,03418	-1,86	0,000
14	F6HKH3	XP_002283632.1	225441000	Enolase 1	4	4.1.11	glycolysis.cytosolic branch.3-phosphoglycerate kinase (PGK)	37,1	12	208,59	1,245	0,12645	1,631	0,07889	1,31	0,027
15	A5CAF6	XP_002263950.1	225464999	Phosphoglycerate kinase	4	4.1.11	glycolysis.cytosolic branch.3-phosphoglycerate kinase (PGK)	41,8	12	206,41	1,103	0,05640	1,110	0,01956	1,01	0,919
16	Q8S568	NP_001268098.1	526117723	Catalase	21	21.6	redox.dismutases and catalases	30	12	202,76	0,626	0,06134	1,636	0,10925	2,61	0,000
17	F6GWF3	XP_002266276.1	225433510	Serine hydroxymethyltransferase	13	13.1.5.2	amino acid metabolism.synthesis.serine-glycine-cysteine group.glycine	32,4	12	175,76	0,404	0,03667	0,553	0,03315	1,37	0,013
					25	25.1	C1-metabolism.glycine hydroxymethyltransferase									
18	A0A1Z2THL4	CBI28009.3	297738764	NADP-dependent malic enzyme	8	8.2.10	TCA / organic transformation.other organic acid transformations.malic	30,7	12	174,74	0,256	0,03327	0,573	0,05102	2,24	0,000
19	D7TFJ4	XP_002271514.1	225425280	Transaldolase	7	7.2.2	OPP.non-reductive PP.transaldolase	33,1	11	216,45	1,072	0,06463	1,038	0,07631	-1,03	0,747
20	F6HTU8	XP_002275990.1	225451235	Cysteine synthase	13	13.1.5.3.1	amino acid metabolism.synthesis.serine-glycine-cysteine group.cysteine.OASTL	26,1	11	203,37	0,990	0,06960	0,841	0,03079	-1,18	0,079
21	A0A1Z2THL9	NP_001268095.1	526117711	Malate dehydrogenase	6	6.3	gluconeogenesis / glyoxylate cycle.malate dehydrogenase	63,3	11	203,15	1,605	0,11006	1,568	0,10011	-1,02	0,812
					8	8.1.9	TCA / organic transformation.TCA.malate DH									
22	F6H5H5	XP_002278190.1	225446489	Chavicol O-methyltransferase	16	16.2	secondary metabolism.phenylpropanoids	36,6	11	200,29	1,817	0,09137	1,498	0,03032	-1,21	0,008
23	F6I0H8	XP_002282276.1	225431563	UTP--glucose-1-phosphate uridylyltransferase	4	4.1.1	glycolysis.cytosolic branch.UGPase	28,4	11	187,59	0,538	0,03234	0,773	0,03545	1,44	0,001
24	D7SYK8	XP_002278181.1	225431960	ATP-citrate synthase beta chain protein 2	8	8.2.11	TCA / organic transformation.other organic acid transformations.atp-citrate lyase	23,3	11	178,16	0,688	0,05286	0,639	0,01335	-1,08	0,381
25	A5C5K3	CAN64242.1	147783188	Adenosylhomocysteinase	13	13.2.3.4	amino acid metabolism.degradation.aspartate family.methionine	28	11	171,18	0,396	0,03365	0,567	0,01653	1,43	0,001
26	P51119	P51119.1	1707959	Glutamine synthetase cytosolic isozyme 2	12	12.2.2	N-metabolism.ammonia metabolism.glutamine synthetase	38,2	11	156,36	1,036	0,14248	0,602	0,04248	-1,72	0,015
27	A5B038	AAZ79355.1	73647513	Aldehyde dehydrogenase	5	5.10	fermentation.aldehyde dehydrogenase	23,7	11	154,48	0,220	0,05111	0,189	0,01086	-1,17	0,559
					29	29.2.2	protein.synthesis.ribosome biogenesis									
28	D7TCM7	CBI27885.3	297738640	UPI00053F79C7	27	27.1.2	RNA.processing.RNA helicase	26,3	11	153,25	0,396	0,03895	0,588	0,03215	1,48	0,004
29	F6GTE2	XP_002263490.1	225456550	Alpha-1,4-glucan-protein synthase [UDP-forming], putative	10	10.5.5	cell wall.cell wall proteins.RGP	37,4	10	151,4	0,532	0,03406	0,532	0,03055	1,00	0,993
30	F6H4T7	XP_002266780	225462164	Elongation factor 2	29	29.2.4	protein.synthesis.elongation	16,2	10	149,77	0,637	0,04407	1,378	0,13981	2,16	0,000
31	F6HGH4	XP_002270984	225425053	6-phosphogluconate dehydrogenase, decarboxylating	7	7.1.3	OPP.oxidative PP.6-phosphogluconate dehydrogenase	21,8	10	141,7	0,460	0,04940	0,704	0,04150	1,53	0,004
32	A5JPK7	NP_001267971.1	526118253	Monodehydroascorbate reductase	21	21.2.1	redox.ascorbate and glutathione.ascorbate	26,2	9	167,52	0,552	0,04079	0,765	0,06783	1,39	0,023
33	A5BV65	CAN70587.1	147784332	Triosephosphate isomerase	4	4.1.7	glycolysis.cytosolic branch.triosephosphate isomerase (TPI)	52,7	9	163,72	0,792	0,05258	0,771	0,03488	-1,03	0,744
34	F6HZK0	XP_002278712.1	225438145	Malate dehydrogenase, cytoplasmic	8	8.2.9	TCA / organic transformation.other organic acid transformations.cyt MDH	32,8	9	161,67	1,506	0,03798	1,400	0,05512	-1,08	0,146
35	E0CQW9	XP_002285897.1	225459744	V-type proton ATPase subunit B1	34	34.1.1.1	transport.p- and v-ATPases.H+-transporting two-sector ATPase.subunit B	23,2	9	153,58	0,405	0,04287	0,349	0,02306	-1,16	0,282
36	F6HR72	XP_002262767.1	225462609	Glutathione S-transferase	26	26.9	misc.glutathione S transferases	39,7	9	148,97	0,865	0,12512	0,449	0,01586	-1,93	0,008
37	Q9M6B5	AAF44335.1	7264740	Alcohol dehydrogenase 6	26	26.11.1	misc.alcohol dehydrogenases.cinnamyl alcohol dehydrogenase	26,5	9	147,21	0,476	0,10217	0,579	0,02932	1,22	0,353
38	F6GT74	XP_002284909.1	225457407	Malate dehydrogenase, mitochondrial	8	8.1.9	TCA / organic transformation.TCA.malate DH	32,4	9	145,21	0,753	0,04278	0,788	0,05093	1,05	0,609
39	F6HBF2	XP_002279748.1	225450149	ADP,ATP carrier protein, mitochondrial	2	2.1.2.5	major CHO metabolism.synthesis.starch.transporter	20,7	9	140,37	0,436	0,05403	1,471	0,11187	3,37	0,000
					34	34.14	transport.unspecified cations									
40	Q3KN68	NP_001268171.1	526118006	Isoflavone reductase-like protein 5	16	16.8.5.1	secondary metabolism.flavonoids.isoflavonols.isoflavone reductase	33,9	9	139,61	0,813	0,05641	0,932	0,03078	1,15	0,093
41	A5BX54	CBI27352.3	297738151	Isocitrate dehydrogenase [NADP]	8	8.1.4	TCA / organic transformation.TCA.IDH	23,7	9	128,29	0,213	0,02814	0,285	0,01528	1,34	0,047
42	D7T0U8	CBI24168.3	297736130	Glyceraldehyde-3-phosphate dehydrogenase	1	1.3.4	PS.calvin cycle.GAP	24,2	9	127,41	0,767	0,05412	0,493	0,01600	-1,56	0,001
43	D7TS57	CBI33329.3	297741894	Chaperonin CPN60-2, mitochondrial	20	20.2.1	stress.abiotic.heat	22,9	9	116,37	0,486	0,06369	0,884	0,05979	1,82	0,001
					29	29.4	protein.postranslational modification									
					29	29.6	protein.folding									
44	F6H440	XP_002275325.1	225451581	Mitochondrial phosphate transporter	34	34.9	transport.metabolite transporters at the mitochondrial membrane	22,8	9	115,68	0,652	0,07483	0,554	0,02278	-1,18	0,240
45	F6HFL6	XP_010662289.1	731369971	Fructose-bisphosphate aldolase	4	4.1.10	glycolysis.cytosolic branch.aldolase	24,9	9	115,15	0,560	0,06442	0,326	0,02479	-1,72	0,007
46	F6I134	XP_002274871.1	225427917	Triosephosphate isomerase, chloroplastic	1	1.3.5	PS.calvin cycle.TPI	35,1	9	115,04	0,271	0,05732	0,088	0,01303	-3,06	0,011
47	F6HDW4	XP_002283898.1	225432858	GDP-mannose 3,5-epimerase	10	10.1.5	cell wall.precursor synthesis.UXS	25,4	8	153,79	0,458	0,02189	0,370	0,01604	-1,24	0,009
					21	21.2.1.1	redox.ascorbate and glutathione.ascorbate.GME									
48	F6I4V3	CBI39638.3	296089819	ADP-ribosylation factor 1-like 2	26	26.1	misc.misc2	26,7	8	135,89	0,945	0,06542	0,627	0,01953	-1,51	0,001
					33	33.99	development.unspecified									
49	Q9M563	ABB82365.1	82547239	Beta-1,3-glucanase	26	26.4.1	misc.beta 1,3 glucan hydrolases.glucan endo-1,3-beta-glucosidase	25,8	8	135,32	0,651	0,11927	0,851	0,02604	1,31	0,131
50	F6H775	NP_001268100.1	526117731	Class I-like SAM-binding methyltransferase superfamily	16	16.2.1.9	secondary metabolism.phenylpropanoids.lignin biosynthesis.COMT	12,8	8	114,06	0,183	0,05495	0,037	0,00270	-4,95	0,024
					26	26.6	misc.O-methyl transferases									
51	F6GTG3	XP_002274334.1	225455784	Enolase 1, chloroplastic-like	4	4.1.13	glycolysis.cytosolic branch.enolase	19,9	8	113,62	0,216	0,04904	0,134	0,00715	-1,61	0,131
52	F6HYJ1	XP_002276114.1	225445867	leucine aminopeptidase 1 isoform X1	29	29.5	protein.degradation	22,6	8	104,11	0,216	0,02298	0,149	0,02166	-1,45	0,061
53	D7T7Y3	CBI26642.3	297737441	Aconitate hydratase 3, mitochondrial	8	8.1.3	TCA / organic transformation.TCA.aconitase	9	8	100,11	0,326	0,04641	0,401	0,03820	1,23	0,238
54	A3QRB7	NP_001267891.1	526117918	Chitinase class I basic	20	20.1.7	stress.biotic.PR-proteins	31,2	7	137,29	0,644	0,08684	1,392	0,03679	2,16	0,000
55	Q2HZF5	NP_001268056.1	526117561	Aquaporin PIP2	34	34.19.1	transport.major intrinsic proteins.PIP	39,6	7	131,63	1,278	0,03928	1,709	0,05251	1,34	0,000
56	A5AKD8	CAN64643.1	147787082	Peptidyl-prolyl cis-trans isomerase	26	26.8	misc.nitrilases, nitrile lyases, berberine bridge enzymes, reticuline oxidases, troponine reductases	48,8	7	129,41	1,564	0,09599	1,949	0,02129	1,25	0,003
					31	31.3.1	cell.cycle.peptidylprolyl isomerase									
57	F6I6R4	XP_002264183.2	359485890	Beta-xylosidase/alpha-L-arabinofuranosidase 2	10	10.6.1	cell wall.degradation.cellulases and beta-1,4-glucanases	11,2	7	127,74	0,309	0,05245	0,444	0,01414	1,44	0,032
					10	10.6.2	cell wall.degradation.mannan-xylose-arabinose-fucose									
58	E0CR49	CBI19003.3	302141800	Protein disulfide-isomerase	21	21.1	redox.thioredoxin	20,8	7	123,72	0,218	0,01695	0,256	0,01338	1,17	0,113
59	F6HAM6	XP_002266494.2	359490179	Transketolase, chloroplastic	7	7.2.1	OPP.non-reductive PP.transketolase	13	7	119,58	0,817	0,08893	0,811	0,06325	-1,01	0,958
60	F6HLJ7	XP_019077386.1	1105499058	Enoyl-[acyl-carrier-protein] reductase [NADH] 1, chloroplastic isoform X1	11	11.1.6	lipid metabolism.FA synthesis and FA elongation.enoyl ACP reductase	21,5	7	119,45	0,594	0,07258	0,352	0,04040	-1,68	0,016
61	A5ANT9	A5ANT9.1	158514257	Chalcone--flavonone isomerase 2	16	16.8.2.2	secondary metabolism.flavonoids.chalcones.chalcone isomerase	38,4	7	115,41	0,411	0,03783	0,407	0,03623	-1,01	0,944
62	F6HJH9	XP_002264055.2	359489076	probable glutathione S-transferase	26	26.9	misc.glutathione S transferases	29	7	113,77	0,901	0,08759	0,832	0,02543	-1,08	0,468

63	F6H7H1	XP_003632941.1	359483345	Procardosin-A	29	29.5.4	protein.degradation.aspartate protease	16,6	7	113,43	0,687	0,06118	0,324	0,02386	-2,12	0,000
64	F6HFF7	XP_002284729.1	225424316	Phosphoglucomutase, cytoplasmic 1	4	4.1.2	glycolysis.cytosolic branch.phosphoglucomutase (PGM)	14,8	7	108,03	0,075	0,01593	0,260	0,05535	3,46	0,009
65	F6H9P9	CBI24059.3	297736021	Biotin carboxylase 1, chloroplastic	11	11.1.1.2.4	lipid metabolism.FA synthesis and FA elongation.acetyl CoA carboxylation.heteromeric complex.biotin carboxylase	16	7	106,66	0,544	0,02572	0,309	0,01197	-1,76	0,000
66	A5C7J5	CAN70962.1	147821099	Thioredoxin family protein	21	21.1	redox.thioredoxin	26	7	106,4	0,313	0,02458	0,302	0,04424	-1,04	0,827
67	A0A024FS61	BAO79387.1	633259653	Polyphenol_oxidase	26	26.7	misc.oxidases - copper, flavone etc	11,2	7	101,58	0,521	0,07684	0,274	0,03133	-1,90	0,014
68	F6HUS6	XP_003633800.1	359488672	Uncharacterized protein	35	35.2	not assigned.unknown	8,6	7	97,4	0,180	0,05558	0,166	0,01103	-1,08	0,809
69	F6HVD5	XP_002264626.1	225465837	aspartyl protease AED3	27	27.3.67	RNA.regulation of transcription.putative transcription regulator	20,3	7	96,45	0,407	0,02975	0,346	0,01252	-1,17	0,091
70	F6GTA6	XP_002272188.1	225456674	Hypersensitive-induced response protein 1 isoform X2	20	20.1.99	stress.biotic.misc	23,3	7	94,27	0,822	0,07864	0,594	0,05653	-1,38	0,040
71	F6GTP0	XP_002279101.1	225456004	Heat shock protein, putative	20	20.1.5	stress.biotic.regulation of transcription	11,4	7	86,86	0,211	0,03234	0,122	0,01023	-1,73	0,026
					20	20.2.1	stress.abiotic.heat									
					27	27.3.3	RNA.regulation of transcription.AP2/EREBP, APETALA2/ethylene-responsive element binding protein family									
72	D7TJI9	CBI30661.3	297740479	Pyruvate decarboxylase 1	5	5.2	fermentation.PDC	12,8	7	83,53	0,193	0,03116	0,102	0,00644	-1,90	0,017
73	A5AS18	CBI20462.3	302143167	Putative quinone reductase	11	11.8	lipid metabolism.exotics (steroids, squalene etc)	40,3	6	125,57	0,906	0,04301	0,638	0,04198	-1,42	0,001
74	F6HNF4	XP_002272669.1	225449018	Adenosine kinase 2	23	23.3.2.1	nucleotide metabolism.salvage.nucleoside kinases.adenosine kinase	25,2	6	115,34	0,277	0,03063	0,292	0,00933	1,05	0,653
75	F6HNS4	CBI31594.3	296086153	Peptidase	29	29.5.1	protein.degradation.subtilases	6,6	6	114,48	1,034	0,11639	1,154	0,04073	1,12	0,355
76	F6I5Y5	XP_002285358.1	225428898	D-3-phosphoglycerate dehydrogenase	13	13.1.5.1.1	amino acid metabolism.synthesis.serine-glycine-cysteine group.serine.phosphoglycerate dehydrogenase	16,5	6	111,79	0,387	0,04651	0,465	0,03435	1,20	0,209
77	C5DB50	XP_002266205.1	225439064	Putative 2,3-bisphosphoglycerate-independent phosphoglycerate mutase	4	4.1.12	glycolysis.cytosolic branch.phosphoglycerate mutase	12,7	6	103,68	0,449	0,01055	0,390	0,02604	-1,15	0,064
78	A5BH43	CAN63486.1	147811059	Carboxypeptidase	29	29.5.5	protein.degradation.serine protease	15,5	6	101,81	0,342	0,06177	0,427	0,03814	1,25	0,265
79	A5BVL9	CAN63896.1	147799894	Glutathione S-transferase GST 19	26	26.9	misc.glutathione S transferases	25,3	6	97,67	0,375	0,01933	0,351	0,02563	-1,07	0,484
					28	28.1	DNA.synthesis/chromatin structure									
					33	33.99	development.unspecified									
80	A5BUU4	A5BUU4.1	229891614	40S ribosomal protein SA	29	29.2.1.2.1.31	protein.synthesis.ribosomal protein.eukaryotic.40S subunit.SA	23	6	97,49	0,163	0,02812	0,317	0,01602	1,95	0,001
					29	29.2.2	protein.synthesis.ribosome biogenesis									
81	F6H5Z7	CBI22354.3	302143601	UPI0008FECB57 - Ubiquitin	29	29.2.2	protein.synthesis.ribosome biogenesis	5,9	6	95,25	0,753	0,05285	0,855	0,03467	1,13	0,140
82	F6GTY8	XP_002284964.1	225435233	Elongation factor 1-alpha	29	29.2.4	protein.synthesis.elongation	10,4	6	86,85	0,513	0,04605	0,603	0,04789	1,18	0,205
83	F6HHQ7	XP_002265690.1	225447510	Acetyl-CoA acetyltransferase 1	13	13.2.3.5	amino acid metabolism.degradation.aspartate family.lysine	26,2	6	86,37	0,194	0,02588	0,241	0,01628	1,24	0,156
					13	13.2.4.5	amino acid metabolism.degradation.branched chain group.isoleucine									
					16	16.1.2.1	secondary metabolism.isoprenoids.mevalonate pathway.acetyl-CoA C-acyltransferase									
84	D7TBL7	XP_002284313.1	225445206	Aspartate aminotransferase	13	13.1.1.2.1	amino acid metabolism.synthesis.central amino acid metabolism.aspartate.aspartate aminotransferase	16,5	6	82,86	0,135	0,01862	0,167	0,01745	1,24	0,239
85	A5AKK0	CAN75773.1	147789187	Putative ripening-related protein	20	20.1	stress.biotic	35,7	6	82,6	0,215	0,06022	0,196	0,02163	0,91	0,770
86	F6GT84	XP_002269118.1	225456761	Glutathione S-transferase U9	26	26.9	misc.glutathione S transferases	25,5	6	80,97	0,606	0,05563	0,364	0,03124	-1,66	0,004
87	F6HPC0	XP_002275338.2	359473386	Glutathione S-transferase U10	26	26.9	misc.glutathione S transferases	23,3	6	80,27	0,330	0,03953	0,226	0,03147	-1,46	0,067
88	D7TAP7	CBI27570.3	297738369	3-oxoacyl-[acyl-carrier-protein] reductase 2, chloroplastic	11	11.1.4	lipid metabolism.FA synthesis and FA elongation.ACP oxoacyl reductase	22,7	6	79,58	0,205	0,02520	0,064	0,01462	-3,19	0,001
					26	26.8	misc.nitrilases, nitrile lyases, berberine bridge enzymes, reticuline oxidases, troponine reductases									
89	A5C6H7	CAN68704.1	147800323	Sucrose synthase	2	2.2.1.5	major CHO metabolism.degradation.sucrose.Susy	7,2	6	79,39	0,020	0,00790	0,220	0,01755	10,91	0,000
90	D7UC33	XP_002267348.2	359489616	UPI0008FEC8A7 - PREDICTED: (+)-neomenthol dehydrogenase isoform X1	26	26.22	misc.short chain dehydrogenase/reductase (SDR)	27,9	6	78,54	0,230	0,04557	0,207	0,02360	-1,11	0,656
91	F6I6W5	XP_002276269.1	225457674	Pyrophosphate--fructose 6-phosphate 1-phosphotransferase subunit alpha	4	4.1.5	glycolysis.cytosolic branch.pyrophosphate-fructose-6-P phosphotransferase	10,2	6	78,09	0,109	0,00822	0,215	0,00909	1,97	0,000
92	F6H0X2	NP_001268096.1	526117715	Phospho-2-dehydro-3-deoxyheptonate aldolase	13	13.1.6.1.1	amino acid metabolism.synthesis.aromatic aa.chorismate.3-deoxy-D-arabino-heptulosonate 7-phosphate synthase	14	6	75,25	0,049	0,02064	0,112	0,02827	2,28	0,103
					34	34.16	transport.ABC transporters and multidrug resistance systems									
93	A5ANH8	CAN71501.1	147765656	Probable mitochondrial-processing peptidase subunit beta, mitochondrial	29	29.3.2	protein.targeting.mitochondria	16,4	6	74,93	0,150	0,01143	0,163	0,00733	1,08	0,377
94	F6I4H0	XP_010659744.1	731416007	S-adenosylmethionine synthase 4	13	13.1.3.4.11	amino acid metabolism.synthesis.aspartate family.methionine.S-adenosylmethionine synthetase	15	6	72,47	0,145	0,03750	0,059	0,01488	-2,43	0,061
95	A9CSL9	BAF95875	163914213	Pathogenesis-related protein 10	20	20.1.7	stress.biotic.PR-proteins	37,3	5	96,94	2,425	0,04355	2,654	0,02637	1,09	0,001
					27	27.1.19	RNA.processing.ribonucleases									
					34	34.16	transport.ABC transporters and multidrug resistance systems									
96	A5AVX9	XP_010661131.1	731419756	UDP-glucose 6-dehydrogenase 1	10	10.1.4	cell wall.precursor synthesis.UGD	13,3	5	85,98	0,093	0,01769	0,080	0,01726	-1,17	0,595
97	C5DB68	CAQ58629.1	239056192	Pyruvate kinase	4	4.1.14	glycolysis.cytosolic branch.pyruvate kinase (PK)	15	5	84,86	0,164	0,02408	0,261	0,00843	-0,63	0,003
					11	11.1.30	lipid metabolism.FA synthesis and FA elongation.pyruvate kinase									
98	L7NSW9	NP_001267918.1	526118016	Plasma membrane 11 aquaporin	34	34.19.1	transport.major intrinsic proteins.PIP	33,9	5	84,27	0,573	0,04353	0,593	0,01129	1,03	0,668
99	A5C8L8	CAN64338.1	147834040	Monodehydroascorbate reductase 5, mitochondrial isoform X1	21	21.2.1	redox.ascorbate and glutathione.ascorbate	6,6	5	80,15	0,136	0,01756	0,176	0,01506	1,30	0,113
100	D7TR81	CBI33004.3	297741775	Pyrophosphate--fructose 6-phosphate 1-phosphotransferase subunit beta	4	4.2.5	glycolysis.plastid branch.pyrophosphate-fructose-6-P phosphotransferase	13,9	5	79,63	0,084	0,01687	0,028	0,00879	-2,96	0,015
101	A0A0M5I8D0	NP_001268034.1	526117485	Flavanone 3-hydroxylase	16	16.8.3.2	secondary metabolism.flavonoids.dihydroflavonols.flavanone 3-hydroxylase	20,9	5	79,41	0,223	0,02597	0,137	0,01944	-1,63	0,024
					17	17.5.1	hormone metabolism.ethylene.synthesis-degradation									
					29	29.2.3	protein.synthesis.initiation									
102	F6GUN2	XP_002285722.1	225435760	Mitochondrial dicarboxylate/tricarboxylate transporter DTC	34	34.9	transport.metabolite transporters at the mitochondrial membrane	16,2	5	79,07	0,181	0,01759	0,204	0,00439	1,13	0,221
103	A5AGI7	CAN66724.1	147767381	Citrate synthase	8	8.1.2	TCA / organic transformation.TCA.CS	7,1	5	75,4	0,188	0,02225	0,183	0,02388	-1,03	0,888
104	D7TBD9	XP_002283310.1	225445041	Probable mitochondrial-processing peptidase subunit alpha-2, chloroplastic/mitochondrial	29	29.3.2	protein.targeting.mitochondria	18,1	5	74,52	0,126	0,02144	0,138	0,02303	1,09	0,712
105	F6I455	XP_002278756.2	359493457	Probable elongation factor 1-gamma 2	29	29.2.4	protein.synthesis.elongation	16,9	5	71,79	0,050	0,01875	0,219	0,03870	4,34	0,003
106	D7TQB0	CBI32630.3	297741498	Glutathione S-transferase	26	26.9	misc.glutathione S transferases	22,3	5	71,36	0,188	0,00938	0,161	0,01397	-1,17	0,140
107	A5AZ36	CBI27343.3	297738142	Glutathione S-transferase U25	26	26.9	misc.glutathione S transferases	20,9	5	69,85	0,421	0,03354	0,133	0,03171	-3,16	0,000
108	D7TP00	XP_002263386.1	225436699	Glutathione S-transferase F13	26	26.9	misc.glutathione S transferases	30,8	5	69,58	0,428	0,02551	0,462	0,01642	1,08	0,290
109	D7SZ58	CBI23542.3	296083547	Ras-related protein RABD2b	30	30.5	signalling.G-proteins	32,8	5	68,49	0,430	0,05535	0,345	0,02417	-1,25	0,191
110	D7T8R2	XP_002284570.1	225424272	MLP-like protein 34	20	20.1	stress.biotic	38,4	5	67,37	0,746	0,06672	1,152	0,08277	1,54	0,003
					20	20.2.99	stress.abiotic.unspecified									
111	F6HXC8	NP_001267897.1	526117940	Phospholipase D	1	1.1.5.1	PS.lightreaction.other electron carrier (ox/red).plastocyanin	8	5	66,15	0,026	0,01270	0,092	0,01136	3,51	0,003
					11	11.9.3.1	lipid metabolism.lipid degradation.lysophospholipases.phospholipase D									
					27	27.3.29	RNA.regulation of transcription.TCP transcription factor family									
112	F6GSZ7	XP_003634222.1	359491108	Omega-hydroxypalmitate O-feruloyl transferase	16	16.2	secondary metabolism.phenylpropanoids	10,2	5	64,76	0,180	0,01708	0,209	0,01930	1,17	0,275
113	F6HIK4	CBI28956.3	297739305	Peroxidase	26	26.12	misc.peroxidases	8	5	64,41	0,089	0,01887	0,119	0,01215	1,34	0,212
114	F6HL42	XP_002272909.1	225440478	NADPH-dependent aldo-keto reductase, chloroplastic	3	3.5	minor CHO metabolism.others	15,4	5	48,83	0,082	0,02221	0,037	0,00827	-2,24	0,083
					16	16.8.2	secondary metabolism.flavonoids.chalcones									
115	D7SLM9	CBI16557.3	297746501	UPI000B789CD1 (1, 29)	1	1.3.13	PS.calvin cycle.rubisco interacting	8,2	4	77,56	0,116	0,01452	0,137	0,01486	1,18	0,350
					29	29.6	protein.folding									
116	A0T2X7	ABK81651.1	118406886	Glutathione S-transferase	26	26.9	misc.glutathione S transferases	23,3	4	76,8	1,268	0,13336	1,010	0,12800	-1,26	0,192
117	A0A0F7J172	ABH09331.1	111379088	Aquaporin TIP2;1	34	34.19.2	transport.major intrinsic proteins.TIP	23,2	4	76,54	1,523	0,12518	1,828	0,13137	1,20	0,124
118	A5B427	CAN60921.1	147838052	Cyclase	28	28.99	DNA.unspecified	15,8	4	72,73	0,244	0,01611	0,343	0,00910	1,41	0,000
119	A5BM68	XP_010649714.1	731388721	Translationally-controlled tumor protein homolog	33	33.99	development.unspecified	28,1	4	71,27	0,433	0,02267	0,374	0,02163	-1,16	0,090
120	F6HLE8	XP_002281550.1	225441583	PREDICTED: 40S ribosomal protein S5	29	29.2.1.2.1.5	protein.synthesis.ribosomal protein.eukaryotic.40S subunit.S5	28,8	4	70,99	0,205	0,01507	0,203	0,02144	-1,01	0,954
121	F6GZY7	XP_002284973.1	225458701	Cysteine proteinase RD21A	29	29.5.3	protein.degradation.cysteine protease	11,9	4	70,73	0,467	0,02112	0,319	0,02178	-1,47	0,001
					34	34.19.1	transport.major intrinsic proteins.PIP									

**122**	D7TRS2	XP_002272479.1	225469754	Mannitol dehydrogenase	16	16.2.1.10	secondary metabolism.phenylpropanoids.lignin biosynthesis.CAD	17,7	4	70,17	**0,369**	0,05821	**0,231**	0,03502	**-1,60**	**0,069**
**123**	F6HPF2	XP_002276636.1	225424908	Mitochondrial outer membrane protein porin 1	34	34.20	transport.porins	19,2	4	68,84	**0,272**	0,01308	**0,251**	0,00396	**-1,08**	**0,154**
**124**	A5BAY1	CAN61170.1	147781964	Germin-like protein 9-2	15	15.2	metal handling.binding, chelation and storage	34,6	4	68,22	**0,420**	0,05674	**0,311**	0,03180	**-1,35**	**0,125**
					20	20.2.99	stress.abiotic.unspecified									
**125**	A5C5V3	CAN81862.1	147860330	Dihydroceramide fatty acyl 2-hydroxylase FAH1	11	11.2.2	lipid metabolism.FA desaturation.a hydroxylase	17	4	67,97	**0,203**	0,05901	**0,029**	0,00735	**-7,02**	**0,015**
					11	11.8.1	lipid metabolism.exotics (steroids, squalene etc).sphingolipids									
**126**	A5AKB1	CAN69132.1	147787229	Plastid-lipid-associated protein 1, chloroplastic (31)	31	31.1	cell.organisation	25,9	4	66,96	**0,091**	0,02330	**0,038**	0,00675	**-2,41**	**0,054**
**127**	F6HFN8	XP_002282287.1	225423947	Dihydrolipoamide acetyltransferase component of pyruvate dehydrogenase complex (8, 11)	8	8.1.1.2	TCA / organic transformation.TCA.pyruvate DH.E2	11,4	4	65,92	**0,220**	0,02595	**0,167**	0,03278	**-1,32**	**0,230**
					11	11.1.31	lipid metabolism.FA synthesis and FA elongation.pyruvate DH									
**128**	D7TVI4	CBI34509	297742360	3-oxoacyl-[acyl-carrier-protein] synthase I, chloroplastic	11	11.1.3	lipid metabolism.FA synthesis and FA elongation.ketoacyl ACP synthase	13,1	4	64,05	**0,113**	0,02916	**0,002**	0,00196	**-57,38**	**0,004**
**129**	Q1AFF4	AAZ79357.1	73647738	Ascorbate peroxidase	21	21.2.1	redox.ascorbate and glutathione.ascorbate	20,8	4	63,03	**0,408**	0,01757	**0,409**	0,02824	**1,00**	**0,984**
**130**	F6H9T6	XP_002265514.1	225462297	Succinate-semialdehyde dehydrogenase, mitochondrial	8	8.1.99	TCA / organic transformation.TCA.misc	10,5	4	62,98	**0,142**	0,03271	**0,348**	0,04781	**2,45**	**0,005**
					8	8.2.99	TCA / organic transformation.other organic acid transformations.misc									
**131**	F6H710	XP_002279647.1	225432012	Galactokinase, putative	3	3.8.1	minor CHO metabolism.galactose.galactokinases	10,9	4	62,71	**0,076**	0,02082	**0,099**	0,01135	**1,29**	**0,366**
**132**	A5BEM8	CBI25677.3	297736660	Oxidoreductase GLYR1	7	7.1.3	OPP.oxidative PP.6-phosphogluconate dehydrogenase	23,3	4	61,84	**0,173**	0,03227	**0,067**	0,01323	**-2,60**	**0,012**
**133**	D7T300	CBI24881.3	297736243	ATP synthase subunit O, mitochondrial	9	9.9	mitochondrial electron transport / ATP synthesis.F1-ATPase	18,7	4	58,69	**0,065**	0,01251	**0,200**	0,01571	**3,06**	**0,000**
**134**	E0CV68	CBI23029.3	302143924	importin subunit beta-1	29	29.3.1	protein.targeting.nucleus	9	4	58,35	**0,201**	0,03205	**0,306**	0,02289	**1,52**	**0,024**
**135**	A5B878	XP_002271352.1	225453350	Nucleoside diphosphate kinase	23	23.4.10	nucleotide metabolism.phosphotransfer and pyrophosphatases.nucleoside diphosphate kinase	27	4	56,77	**0,298**	0,09164	**0,168**	0,02402	**-1,77**	**0,200**
**136**	A5AXI6	CAN80537.1	147843260	60S acidic ribosomal protein P0	29	29.2.1.2.2.80	protein.synthesis.ribosomal protein.eukaryotic.60S subunit.P0	26,8	4	56,7	**0,359**	0,02975	**0,324**	0,09012	**-1,11**	**0,721**
**137**	F6HXK4	CAN80223.1	147853720	Plasma membrane ATPase	34	34.1.2	transport.p- and v-ATPases.H+-exporting ATPase	5,3	4	55,85	**0,074**	0,01020	**0,132**	0,03034	**1,77**	**0,102**
**138**	F6HJB9	XP_002265072.1	225468829	Uncharacterized protein	35	35.1	not assigned.no ontology	14,2	4	55,76	**0,107**	0,01451	**0,082**	0,00323	**-1,31**	**0,124**
**139**	A5CAL1	CAN84153.1	147843670	Glyoxylate/hydroxypyruvate reductase A HPR2	1	1.2.6	PS.photorespiration.hydroxypyruvate reductase	12,1	4	54,65	**0,159**	0,00911	**0,110**	0,00636	**-1,45**	**0,001**
					13	13.2.5.2	amino acid metabolism.degradation.serine-glycine-cysteine group.glycine									
					26	26.1	misc.misc2									
**140**	F6HXL1	CBI35797.3	297742930	Proteasome subunit alpha type	29	29.5.11.20	protein.degradation.ubiquitin.proteasom	15	4	53,62	**0,097**	0,00831	**0,089**	0,01144	**-1,09**	**0,585**
**141**	D7TVI4	XP_002265207.2	731378531	3-oxoacyl-[acyl-carrier-protein] synthase I, chloroplastic	11	11.1.3	lipid metabolism.FA synthesis and FA elongation.ketoacyl ACP synthase	14,3	4	53,02	**0,082**	0,02872	**0,000**	0,00000	**d.**	**0,017**
**142**	A5ALB2	XP_002271929.1	225442079	Proteasome subunit alpha type	29	29.5.11.20	protein.degradation.ubiquitin.proteasom	18,2	4	49,47	**0,014**	0,00864	**0,019**	0,00875	**1,36**	**0,694**
**143**	D7TGR6	XP_002264444.1	225465200	Ras-related protein RABA2a	30	30.5	signalling.G-proteins	20	4	49,29	**0,221**	0,02682	**0,164**	0,00722	**-1,35**	**0,067**
**144**	A5BV59	XP_002281279.1	225456096	Guanine nucleotide-binding protein subunit beta-like protein	27	27.3.99	RNA.regulation of transcription.unclassified	15,9	4	48,94	**0,057**	0,01025	**0,076**	0,01213	**1,33**	**0,263**
					29	29.4	protein.postranslational modification									
					30	30.5	signalling.G-proteins									
					33	33.99	development.unspecified									
**145**	F6GWS4	XP_002275309.1	225431269	Peroxidase 52	26	26.12	misc.peroxidases	15	4	48,86	**0,023**	0,01312	**0,086**	0,02944	**3,75**	**0,078**
**146**	F6HE11	XP_002263180.1	225432110	Dihydrolipoyl dehydrogenase	8	8.1.1.3	TCA / organic transformation.TCA.pyruvate DH.E3	9,5	4	48,75	**0,051**	0,01455	**0,081**	0,00750	**1,57**	**0,102**
					21	21.2.2	redox.ascorbate and glutathione.glutathione									
**147**	E0CTI4	CBI21899.3	302143338	26S proteasome non-ATPase regulatory subunit 2 homolog	29	29.5.11.20	protein.degradation.ubiquitin.proteasom	7,4	4	48,49	**0,029**	0,00689	**0,042**	0,01030	**1,44**	**0,329**
**148**	D7SL25	XP_002285368.1	225435395	Cinnamoyl-CoA reductase-like protein	16	16.8.3	secondary metabolism.flavonoids.dihydroflavonols	10,8	4	48,08	**0,077**	0,01319	**0,094**	0,01167	**1,22**	**0,367**
**149**	F6I581	XP_002273244.1	225462013	Heat shock protein 90-2	20	20.2.1	stress.abiotic.heat	7,3	4	47,66	**0,125**	0,00513	**0,098**	0,02569	**-1,27**	**0,339**
**150**	E0CSF7	XP_019072855.1	1104683316	D-amino-acid transaminase, chloroplastic isoform X1	13	13.1	amino acid metabolism.synthesis	12,5	4	46,72	**0,088**	0,01128	**0,105**	0,00217	**1,19**	**0,167**
**151**	F6HC76	CAN61687.1	147839972	Superoxide dismutase	21	21.6	redox.dismutases and catalases	15,9	4	46,55	**0,086**	0,01455	**0,084**	0,00490	**-1,02**	**0,896**
**152**	F6HGZ9	XP_002275155.1	225444613	Sucrose synthase	2	2.2.1.5	major CHO metabolism.degradation.sucrose.Susy	4,6	4	46,26	**0,032**	0,01094	**0,100**	0,02448	**3,11**	**0,030**
**153**	A5AY42	XP_002277452.1	225449132	ATP synthase subunit d, mitochondrial	9	9.9	mitochondrial electron transport / ATP synthesis.F1-ATPase	22,6	4	46,22	**0,079**	0,02723	**0,030**	0,01882	**-2,64**	**0,171**
**154**	A5BXT5	XP_002280606.1	225434259	Guanosine nucleotide diphosphate dissociation inhibitor	30	30.5	signalling.G-proteins	12,3	4	45,44	**0,029**	0,00839	**0,054**	0,01546	**1,87**	**0,183**
**155**	A5ATW2	CAN60563.1	147837952	Bifunctional 3-dehydroquinate dehydratase/shikimate dehydrogenase	13	13.1.6.1.10	amino acid metabolism.synthesis.aromatic aa.chorismate.3-dehydroquinate dehydratase/shikimate dehydrogenase	5,1	4	42,45	**0,062**	0,00877	**0,038**	0,00356	**-1,61**	**0,033**
**156**	A5BYC0	XP_003632316.1	359479647	Proteasome subunit beta type	29	29.5.11.20	protein.degradation.ubiquitin.proteasom	19	4	38,84	**0,142**	0,00502	**0,131**	0,00273	**-1,08**	**0,088**
**157**	A5BR79	XP_002285103.1	225428869	Glycosyltransferase	26	26.2	misc.UDP glucosyl and glucoronyl transferases	10,7	4	37,54	**0,068**	0,01411	**0,095**	0,00949	**1,40**	**0,144**
**158**	D7U3W2	XP_002270126.1	225427465	Bifunctional nitrilase/nitrile hydratase NIT4B	16	16.5.1.3.3	secondary metabolism.sulfur-containing.glucosinolates.degradation.nitrilase	15,5	3	61,15	**0,230**	0,07164	**0,316**	0,02706	**1,37**	**0,288**
					26	26.8	misc.nitrilases, nitrile lyases, berberine bridge enzymes, reticuline oxidases, troponine reductases									
**159**	D7U2H1	XP_002275765.1	225437537	GTP-binding protein SAR1A	30	30.5	signalling.G-proteins	23,8	3	59,79	**0,288**	0,03147	**0,324**	0,01465	**1,13**	**0,319**
**160**	D7SW04	CBI21453.3	296082448	Bifunctional aspartate aminotransferase and glutamate/aspartate-prephenate aminotransferase isoform X2	13	13.1.1.2.1	amino acid metabolism.synthesis.central amino acid metabolism.aspartate.aspartate aminotransferase	11,7	3	57,75	**0,206**	0,03148	**0,114**	0,01439	**-1,80**	**0,024**
					13	13.2.6.2	amino acid metabolism.degradation.aromatic aa.tyrosine									
**161**	A5C9G0	XP_002274407.1	225449052	60S ribosomal protein L12	15	15.2	metal handling.binding, chelation and storage	22,2	3	56,75	**0,271**	0,02164	**0,283**	0,00471	**1,04**	**0,609**
					29	29.2.1.2.2.12	protein.synthesis.ribosomal protein.eukaryotic.60S subunit.L12									
					29	29.2.2	protein.synthesis.ribosome biogenesis									
**162**	D7TRU0	CBI33216.3	297741856	Cinnamyl alcohol dehydrogenase 8	16	16.2.1.10	secondary metabolism.phenylpropanoids.lignin biosynthesis.CAD	9,6	3	56,55	**0,304**	0,02781	**0,271**	0,00289	**-1,12**	**0,266**
**163**	D7TKM8	CBI31050.3	297740868	Germin-like protein 2-1	12	12.2.2	N-metabolism.ammonia metabolism.glutamine synthetase	20,9	3	55,51	**0,227**	0,03532	**0,361**	0,03458	**1,59**	**0,022**
					20	20.2.99	stress.abiotic.unspecified									
					27	27.3.69	RNA.regulation of transcription.SET-domain transcriptional regulator family									
					30	30.3	signalling.calcium									
					34	34.12	transport.metal									
**164**	F6HC36	XP_002280158.1	225448675	Chalcone-flavonone isomerase family protein	16	16.8.2	secondary metabolism.flavonoids.chalcones	19	3	55,23	**0,191**	0,03753	**0,119**	0,01173	**-1,60**	**0,098**
**165**	E0CR38	CBI18992.3	302141789	Proteasome subunit beta type	29	29.5.11.20	protein.degradation.ubiquitin.proteasom	24,5	3	55,07	**0,160**	0,02107	**0,094**	0,02028	**-1,71**	**0,046**
**166**	F6HL77	CBI30508.3	297740326	Tropinone reductase homolog At1g07440	26	26.8	misc.nitrilases, nitrile lyases, berberine bridge enzymes, reticuline oxidases, troponine reductases	15,2	3	51,37	**0,091**	0,03186	**0,076**	0,00276	**-1,20**	**0,649**
**167**	F6HZD8	CBI37013.3	297744043	Short-chain dehydrogenase reductase 3b-like	26	26.22	misc.short chain dehydrogenase/reductase (SDR)	10,2	3	50,56	**0,307**	0,05128	**0,029**	0,01856	**-10,74**	**0,000**
**168**	D7STF0	CBI20064.3	296081541	3-hydroxyacyl-[acyl-carrier-protein] dehydratase FabZ	11	11.1.5	lipid metabolism.FA synthesis and FA elongation.beta hydroxyacyl ACP dehydratase	15,3	3	49,74	**0,231**	0,03318	**0,119**	0,01540	**-1,94**	**0,012**
**169**	A5AGN5	XP_002280094.1	225446579	Ketol-acid reductoisomerase	13	13.1.4.1.2	amino acid metabolism.synthesis.branched chain group.common.ketol-acid reductoisomerase	6,6	3	49,73	**0,101**	0,01836	**0,082**	0,01207	**-1,23**	**0,417**
**170**	F6HP23	XP_002264455.1	225470723	Stilbene synthase 1	16	16.8.2.1	secondary metabolism.flavonoids.chalcones.naringenin-chalcone synthase	7,3	3	49,13	**0,207**	0,00986	**0,178**	0,00755	**-1,16**	**0,041**
**171**	A5ARL2	NP_001268191.1	526118089	Peroxiredoxin	21	21.5	redox.peroxiredoxin	20,9	3	48,87	**0,250**	0,01991	**0,261**	0,02087	**1,04**	**0,727**
**172**	D7SPF1	CBI17530.3	297735168	Succinate dehydrogenase [ubiquinone] flavoprotein subunit, mitochondrial	8	8.1.7	TCA / organic transformation.TCA.succinate dehydrogenase	5,8	3	48,1	**0,081**	0,01307	**0,149**	0,01961	**1,85**	**0,015**
**173**	D7U7S6	XP_002279266.1	225453909	Proteasome subunit beta type	17	17.6.3	hormone metabolism.gibberelin.induced-regulated-responsive-activated	23,7	3	47,75	**0,154**	0,01646	**0,160**	0,02411	**1,03**	**0,862**
					26	26.10	misc.cytochrome P450									
					29	29.5.11.20	protein.degradation.ubiquitin.proteasom									
**174**	A5B729	CAN78723.1	147860147	Uncharacterized protein	35	35.2	not assigned.unknown	11,4	3	47,29	**0,224**	0,03932	**0,011**	0,00495	**-21,21**	**0,000**
**175**	F6HXK8	XP_002265171.1	225441977	Pectinesterase	10	10.8.1	cell wall.pectinesterases.PME	5	3	47,18	**0,079**	0,01150	**0,096**	0,01151	**1,21**	**0,324**
**176**	F6GX20	XP_002277981.1	225430941	4-hydroxy-4-methyl-2-oxoglutarate aldolase (25)	25	25	C1-metabolism	15,1	3	47,14	**0,100**	0,01367	**0,103**	0,00922	**1,03**	**0,851**
**177**	A0A172QNQ9	XP_003631658.1	359475330	Glycine-rich RNA-binding protein 2A	27	27.3.75	RNA.regulation of transcription.GRP	22,2	3	46,81	**0,215**	0,02482	**0,157**	0,03144	**-1,37**	**0,179**
					27	27.4	RNA.RNA binding									
**178**	A5BQN6	XP_003631573.1	359475042	Peptidyl-prolyl cis-trans isomerase	26	26.8	misc.nitrilases, nitrile lyases, berberine bridge enzymes, reticuline oxidases, troponine reductases	20,3	3	46,35	**0,028**	0,02147	**0,088**	0,02812	**3,15**	**0,119**
					31	31.3.1	cell.cycle.peptidylprolyl isomerase									
**179**	F6I1P0	XP_002264210.1	225425166	Pyruvate dehydrogenase E1 component subunit beta (8)	8	8.1.1.1	TCA / organic transformation.TCA.pyruvate DH.E1	10,3	3	45,69	**0,032**	0,00860	**0,041**	0,00664	**1,25**	**0,468**
**180**	D7U5C1	NP_001268084.1	526117669	Ripening-related protein-like	13	13.1.3.4	amino acid metabolism.synthesis.aspartate family.methionine	23,3	3	44,99	**0,407**	0,07019	**0,238**	0,04061	**-1,71**	**0,065**

**181**	D7T2N7	CBI24768.3	296084380	Late embryogenesis abundant protein Lea14-A (3)	33	33.2	development.late embryogenesis abundant	7,5	3	44,83	**0,115**	0,01075	**0,097**	0,00323	**-1,19**	**0,139**
**182**	F6HZ27	XP_002262842.1	225437288	Glutathione S-transferase	26	26.9	misc.glutathione S transferases	13	3	44,55	**0,517**	0,02535	**0,423**	0,01994	**-1,22**	**0,016**
**183**	D7U564	XP_002278162.1	225428005	Proteasome subunit alpha type	17	17.8.1	hormone metabolism.salicylic acid.synthesis-degradation	17,2	3	43,42	**0,087**	0,01630	**0,074**	0,01193	**-1,18**	**0,533**
					29	29.4	protein.postranslational modification									
					29	29.5.11.20	protein.degradation.ubiquitin.proteasom									
					33	33.99	development.unspecified									
**184**	A5ASS2	CAN71616.1	147797244	Thaumatin (20)	20	20.1	stress.biotic	17,8	3	43,2	**0,199**	0,03955	**0,224**	0,02820	**1,13**	**0,616**
					20	20.2	stress.abiotic									
**185**	F6H1D7	CBI19568.3	302142365	Carboxypeptidase	29	29.5.5	protein.degradation.serine protease	7,1	3	42,14	**0,440**	0,06695	**0,080**	0,02099	**-5,53**	**0,000**
**186**	A5C2G6	XP_002277763.1	225434588	Peptidyl-prolyl cis-trans isomerase	31	31.3.1	cell.cycle.peptidylprolyl isomerase	16,7	3	41,35	**0,078**	0,00836	**0,055**	0,01223	**-1,41**	**0,158**
**187**	F6I5I7	XP_002272730.1	225436253	Methylenetetrahydrofolate reductase	25	25.6	C1-metabolism.methylenetetrahydrofolate reductase	6,2	3	40,2	**0,054**	0,01458	**0,121**	0,01709	**2,24**	**0,014**
**188**	D7T8G2	CBI26783.3	297737582	Purple acid phosphatase	26	26.13	misc.acid and other phosphatases	10,4	3	40,01	**0,087**	0,01111	**0,002**	0,00119	**-54,69**	**0,000**
**189**	H2DL86	AEY70471.1	373431037	Plastid lipid-associated protein	35	35.2	not assigned.unknown	11,7	3	39,89	**0,073**	0,01683	**0,037**	0,00485	**-1,95**	**0,069**
**190**	D7T9L8	CBI27189.3	297737988	Coatomer subunit delta	31	31.4	cell.vesicle transport	5,7	3	39,63	**0,137**	0,02575	**0,062**	0,00614	**-2,20**	**0,018**
**191**	D7TWQ4	XP_002278444.1	225452472	Formate dehydrogenase, mitochondrial	25	25.10	C1-metabolism.formate dehydrogenase	10,7	3	39,6	**0,071**	0,03911	**0,152**	0,03346	**2,13**	**0,148**
**192**	F6HI27	XP_002269441.1	225430650	Pyruvate dehydrogenase E1 component subunit beta-2, chloroplastic	1	1.3.8	PS.calvin cycle.transketolase	8,6	3	39,04	**0,124**	0,01741	**0,033**	0,00510	**-3,75**	**0,001**
					8	8.1.1.1	TCA / organic transformation.TCA.pyruvate DH.E1									
					11	11.1.31	lipid metabolism.FA synthesis and FA elongation.pyruvate DH									
**193**	F6H8F3	XP_003635669.1	359497849	Phosphoserine aminotransferase	13	13.1.5.1.2	amino acid metabolism.synthesis.serine-glycine-cysteine group.serine.phosphoserine aminotransferase	10	3	38,9	**0,046**	0,02667	**0,013**	0,00314	**-3,45**	**0,255**
					27	27.3.22	RNA.regulation of transcription.homeobox transcription factor family (HB)									
**194**	F6H727	XP_002277653.1	225431940	Universal stress protein PHOS32 isoform X1	20	20.2.2	stress.abiotic.cold	20	3	38,69	**0,084**	0,01474	**0,052**	0,00578	**-1,60**	**0,075**
**195**	Q944W8	AAL09046.1	15811654	Stilbene synthase 1	16	16.8.2.1	secondary metabolism.flavonoids.chalcones.naringenin-chalcone synthase	7,6	3	38,26	**0,205**	0,03197	**0,185**	0,00326	**-1,11**	**0,541**
**196**	D7SUQ2	CBI21001.3	296081996	GTP-binding nuclear protein	30	30.5	signalling.G-proteins	9,5	3	38,26	**0,109**	0,01150	**0,097**	0,00401	**-1,13**	**0,328**
**197**	A5AKL4	CAN64655.1	147788834	Cysteine protease, putative	29	29.5.3	protein.degradation.cysteine protease	9,3	3	35,68	**0,122**	0,04129	**0,000**	0,00000	**d**	**0,015**
					34	34.19.1	transport.major intrinsic proteins.PIP									
**198**	A5BE54	XP_002282471.1	225452700	Mitochondrial outer membrane protein porin 3	18	18.5.2.1	Co-factor and vitamine metabolism.folate and vitamine K.vitamine K.isochorismate synthase	8,3	3	35,45	**0,148**	0,00864	**0,148**	0,00849	**1,00**	**0,993**
					34	34.2	transport.sugars									
					34	34.20	transport.porins									
**199**	F6H116	XP_002285696.1	225459126	Glucose-6-phosphate isomerase	4	4.2.3	glycolysis.plastid branch.glucose-6-phosphate isomerase	6,2	3	33,24	**0,036**	0,00909	**0,028**	0,00653	**-1,31**	**0,458**
**200**	F6HDM0	XP_002280658.1		Stem-specific protein TSJT1	15	15	metal handling	16,2	3	29,01	**0,007**	0,00745	**0,005**	0,00339	**-1,61**	**0,738**
					17	17.2.3	hormone metabolism.auxin.induced-regulated-responsive-activated									
**201**	D7TW90	CBI34765.3	297742616	Cucumsin	29	29.5.1	protein.degradation.subtilases	5,3	3	28,92	**0,068**	0,01223	**0,000**	0,00000	**d.**	**0,000**
**202**	A5C3G7	XP_002282021.1	225458237	gamma carbonic anhydrase 1, mitochondrial	9	9.1.1.5	mitochondrial electron transport / ATP synthesis.NADH-DH (type I).complex I.carbonic anhydrase	18,7	3	26,26	**0,010**	0,00559	**0,000**	0,00000	**d.**	**0,117**
**203**	D7T4I1	CBI25413.3	297736542	Biotin carboxyl carrier protein of acetyl-CoA carboxylase 2, chloroplastic	11	11.1.1	lipid metabolism.FA synthesis and FA elongation.acetyl CoA carboxylation	15,2	2	42,49	**0,160**	0,02622	**0,054**	0,01027	**-2,98**	**0,004**
**204**	F6HS56	XP_002285645.1	225433414	Potassium channel beta, putative	17	17.2.3	hormone metabolism.auxin.induced-regulated-responsive-activated	10	2	42,23	**0,095**	0,00668	**0,094**	0,01785	**-1,02**	**0,923**
					34	34.15	transport.potassium									
**205**	F6GU75	XP_002276130.2	359478860	26S proteasome regulatory subunit 6B homolog	29	29.5.11.20	protein.degradation.ubiquitin.proteasom	7,4	2	39,98	**0,177**	0,01992	**0,179**	0,02782	**1,01**	**0,957**
**206**	D7SY46	CBI22903.3	296083267	Dihydrolipoyl dehydrogenase 2, chloroplastic (8, 11, 21)	8	8.1.1.3	TCA / organic transformation.TCA.pyruvate DH.E3	5,6	2	37,93	**0,112**	0,01431	**0,103**	0,01528	**-1,09**	**0,680**
					11	11.1.31	lipid metabolism.FA synthesis and FA elongation.pyruvate DH									
					21	21.2.2	redox.ascorbate and glutathione.glutathione									
**207**	C0KY93	ACN38270.1	224038270	Leucoanthocyanidin dioxygenase	1	1.2.6	PS.photorespiration.hydroxypyruvate reductase	7,8	2	37,48	**0,158**	0,03353	**0,048**	0,00197	**-3,29**	**0,008**
					7	7.1.3	OPP.oxidative PP.6-phosphogluconate dehydrogenase									
					13	13.2.5.1	amino acid metabolism.degradation.serine-glycine-cysteine group.serine									
					13	13.2.5.2	amino acid metabolism.degradation.serine-glycine-cysteine group.glycine									
					16	16.8.1.1	secondary metabolism.flavonoids.anthocyanins.leucocyanidin dioxygenase									
					17	17.5.1	hormone metabolism.ethylene.synthesis-degradation									
					26	26.1	misc.misc2									
**208**	F6I675	XP_002279182.1	225454385	Class III chitinase	20	20.1.7	stress.biotic.PR-proteins	14,7	2	35,53	**0,120**	0,04917	**0,032**	0,00859	**-3,78**	**0,107**
					26	26.2	misc.UDP glucosyl and glucoronyl transferases									
**209**	D7U318	XP_002272576.3	1105495218	Dolichyl-diphosphooligosaccharide--protein glycosyltransferase subunit 2	33	33.99	development.unspecified	4,4	2	35,22	**0,222**	0,05391	**0,282**	0,05313	**1,27**	**0,447**
**210**	F6HZ19	CBI36810.3	296088365	Germin-like protein subfamily 1 member 17	12	12.2.2	N-metabolism.ammonia metabolism.glutamine synthetase	12,2	2	35,16	**0,048**	0,00855	**0,057**	0,00491	**1,18**	**0,402**
					20	20.2.99	stress.abiotic.unspecified									
					27	27.3.69	RNA.regulation of transcription.SET-domain transcriptional regulator family									
					34	34.12	transport.metal									
**211**	D7TS82	XP_002285653.1	225433424	Acetyl-CoA C-acyltransferase - 3-ketoacyl-CoA thiolase 2, peroxisomal	11	11.9.4.5	lipid metabolism.lipid degradation.beta-oxidation.acyl-CoA thioesterase	6,9	2	34,23	**0,043**	0,01895	**0,024**	0,00059	**-1,79**	**0,335**
					13	13.2.4.1	amino acid metabolism.degradation.branched chain group.shared									
					13	13.2.4.5	amino acid metabolism.degradation.branched chain group.isoleucine									
**212**	F6I1D6	XP_002265749.2	359489134	Non-specific phospholipase C3 isoform X1	11	11.9.3.5	lipid metabolism.lipid degradation.lysophospholipases.phosphoinositide phospholipase C	4,6	2	34,19	**0,050**	0,01098	**0,052**	0,00102	**1,03**	**0,892**
**213**	A5BVM7	CAN83622.1	147864968	O-methyltransferase YrrM	16	16.2.1.6	secondary metabolism.phenylpropanoids.lignin biosynthesis.CCoAOMT	1,4	2	34,12	**0,046**	0,01080	**0,006**	0,00246	**-7,23**	**0,005**
**214**	D7UBH2	XP_002266527.1	225452196	Glucose-6-phosphate 1-dehydrogenase	7	7.1.1	OPP.oxidative PP.glucose-6-phosphate 1-dehydrogenase (G6PD)	6,3	2	34,03	**0,081**	0,00851	**0,068**	0,01183	**-1,20**	**0,367**
**215**	D7SYB5	XP_010649574.1	731388373	Epoxide hydrolase, putative	26	26.1	misc.misc2	8,2	2	33,78	**0,078**	0,03828	**0,000**	0,00000	**d.**	**0,070**
**216**	F6HX49	CBI36206.3	297743339	Gibberellin 20 oxidase 1 (16, 17, 26)	16	16.5.1.1.1.11	secondary metabolism.sulfur-containing.glucosinolates.synthesis.aliphatic.glucosinolate 2-oxoglutarate-dependent dioxygenase (AOP)	7,9	2	33,7	**0,099**	0,01440	**0,062**	0,01492	**-1,59**	**0,107**
					17	17.5.1	hormone metabolism.ethylene.synthesis-degradation									
					17	17.6.1	hormone metabolism.gibberelin.synthesis-degradation									
					17	17.6.1.11	hormone metabolism.gibberelin.synthesis-degradation.GA20 oxidase									
					26	26.14	misc.oxygenases									
**217**	A5C997	XP_002262780.1	225470692	Membrane steroid-binding protein 2	21	21.2	redox.ascorbate and glutathione	13,9	2	33,28	**0,153**	0,03727	**0,146**	0,02152	**-1,05**	**0,867**
**218**	F6HIC8	CBI28822.3	297739171	Dienelactone hydrolase	26	26.1	misc.misc2	6,5	2	32,04	**0,052**	0,00623	**0,074**	0,00297	**1,42**	**0,011**
**219**	F6H120	XP_002283889.2	359492937	Aspartyl protease AED3	27	27.3.99	RNA.regulation of transcription.unclassified	10,4	2	31,85	**0,053**	0,01919	**0,041**	0,01035	**-1,28**	**0,613**
					29	29.5.4	protein.degradation.aspartate protease									
**220**	D7TRJ7	XP_010646198.1	731439308	fumarylacetoacetase	13	13.2.6.2.6	amino acid metabolism.degradation.aromatic aa.tyrosine.fumarylacetoacetase	6,2	2	31,65	**0,078**	0,01070	**0,069**	0,01288	**-1,12**	**0,631**
**221**	A5BIH7	CAN67965.1	147839059	Peptidase_S10 domain-containing protein	29	29.5.5	protein.degradation.serine protease	5,2	2	30,61	**0,027**	0,00906	**0,003**	0,00345	**-7,81**	**0,036**
**222**	D7SGX1	CBI15651.3	297734404	Elongation factor 1-delta 1	29	29.2.4	protein.synthesis.elongation	8,1	2	30,26	**0,095**	0,01881	**0,108**	0,01429	**1,13**	**0,613**
**223**	F6HTU0	XP_002276988.1	225451257	26S proteasome non-ATPase regulatory subunit 11 homolog	29	29.5.11.20	protein.degradation.ubiquitin.proteasom	6,3	2	30,17	**0,069**	0,01224	**0,053**	0,00830	**-1,31**	**0,298**
**224**	D7SJV3	CBI15929.3	297745873	Clathrin heavy chain	31	31.4	cell.vesicle transport	1,9	2	29,57	**0,000**	0,00000	**0,035**	0,00658	**New**	**0,000**
**225**	A5ARE0	CAN80347.1	147858030	Glutelin type-A 1-like	28	28.2	DNA.repair	8,9	2	29,42	**0,009**	0,00543	**0,064**	0,01275	**7,40**	**0,003**
					33	33.1	development.storage proteins									
**226**	D7SJF5	CBI15781.3	297745725	Uncharacterized protein	35	35.1	not assigned.no ontology	8	2	29,25	**0,057**	0,02005	**0,111**	0,00271	**1,96**	**0,022**
**227**	A5BF93	CBI37148.3	297744178	Succinate--CoA ligase [ADP-forming] subunit beta, mitochondrial	8	8.1.6	TCA / organic transformation.TCA.succinyl-CoA ligase	6,1	2	29,1	**0,046**	0,01718	**0,158**	0,02351	**3,44**	**0,003**
**228**	D7T9K4	CBI27175.3	297737974	Uncharacterized protein	35	35.1	not assigned.no ontology	8,9	2	28,2	**0,205**	0,10510	**0,625**	0,01356	**3,04**	**0,003**
**229**	A5C9Z5	CAN66540.1	147800093	V-type proton ATPase subunit E3	29	29.2.3	protein.synthesis.initiation	7,1	2	28,16	**0,036**	0,01340	**0,022**	0,01017	**-1,59**	**0,448**
					34	34.1.1.4	transport.p- and v-ATPases.H+-transporting two-sector ATPase.subunit E									
**230**	I6VD70	AFN21526.1	393717979	Stilbene synthase	16	16.8.2.1	secondary metabolism.flavonoids.chalcones.naringenin-chalcone synthase	5,6	2	27,99	**0,094**	0,02697	**0,093**	0,02351	**-1,01**	**0,976**
**231**	F6HQA7	NP_001268194.1	526118101	Nitrite reductase 1	12	12.1.2	N-metabolism.nitrate metabolism.nitrite reductase	3	2	27,71	**0,010**	0,00969	**0,040**	0,00677	**4,11**	**0,029**

**232**	Q0MYQ7	NP_001267914.1	526118000	Germin-like protein 2 (15)	15	15.2	metal handling.binding, chelation and storage	9,2	2	27,42	**0,077**	0,05031	**0,112**	0,02251	**1,46**	**0,536**
					20	20.2.99	stress.abiotic.unspecified									
**233**	F6I390	XP_002264156.1	225435874	Pectinesterase	10	10.8.1	cell wall.pectinesterases.PME	5,2	2	27,36	**0,007**	0,00723	**0,042**	0,01054	**5,87**	**0,020**
**234**	D7TE48	CBI28771.3	297739120	Soluble epoxide hydrolase	26	26.1	misc.misc2	10,5	2	27,09	**0,049**	0,01367	**0,010**	0,00664	**-4,66**	**0,030**
**235**	D7SHY3	CBI15093.3	297733846	Betaine aldehyde dehydrogenase 1, chloroplastic	5	5.10	fermentation.aldehyde dehydrogenase	4,9	2	26,31	**0,005**	0,00493	**0,042**	0,01062	**8,62**	**0,009**
					16	16.4.2.1	secondary metabolism.N misc.betaine.betaine-aldehyde dehydrogenase									
**236**	A5ACR7	XP_002282568.1	225452712	V-type proton ATPase subunit	34	34.1.1	transport.p- and v-ATPases.H+-transporting two-sector ATPase	7,1	2	26,13	**0,040**	0,01085	**0,025**	0,01020	**-1,61**	**0,335**
**237**	D7SVJ2	XP_002275510.1	225451579	V-type proton ATPase subunit C	34	34.1	transport.p- and v-ATPases	6,4	2	26,06	**0,053**	0,01119	**0,087**	0,01255	**1,62**	**0,077**
**238**	F6HBQ2	XP_002275510.1	225451579	Pyruvate kinase	34	34.1	transport.p- and v-ATPases	2,9	2	26,03	**0,052**	0,01158	**0,063**	0,00292	**1,21**	**0,389**
**239**	F6HZ29	XP_010652165.1	731395413	bifunctional protein FolD 2	25	25.5	C1-metabolism.methylenetetrahydrofolate dehydrogenase and methenyltetrahydrofolate cyclohydrolase	10,4	2	25,86	**0,031**	0,00549	**0,035**	0,00452	**1,12**	**0,617**
**240**	D7SHR5	XP_002282681.2	359491538	Carbonic anhydrase	8	8.3	TCA / organic transformation.carbonic anhydrases	9,1	2	25,79	**0,000**	0,00000	**0,050**	0,02710	**New**	**0,098**
					16	16.99	secondary metabolism.unspecified									
**241**	A5B174	CAN78540.1	147853311	Perakine reductase (hypothetical protein VITISV_025505)	17	17.2.3	hormone metabolism.auxin.induced-regulated-responsive-activated	2,8	2	25,18	**0,006**	0,00612	**0,066**	0,00398	**10,76**	**0,000**
**242**	D7TSX2	XP_002266288.2	359488227	probable S-adenosylmethionine-dependent methyltransferase At5g38780	13	13.1.3.4.13	amino acid metabolism.synthesis.aspartate family.methionine.methionine S-methyltransferase	5,3	2	24,94	**0,025**	0,01209	**0,009**	0,00218	**-2,69**	**0,235**
					17	17.8.1	hormone metabolism.salicylic acid.synthesis-degradation									
**243**	A5B0N6	XP_002264813.1	225462628	Glutathione S-transferase parA	26	26.9	misc.glutathione S transferases	6,7	2	24,67	**0,128**	0,02782	**0,132**	0,01904	**1,03**	**0,907**
**244**	F6I407	XP_002267428.1	225460394	Patellin-3	29	29.3.4.99	protein.targeting.secretory pathway.unspecified	4,1	2	24,55	**0,038**	0,00765	**0,077**	0,02340	**2,01**	**0,149**
					34	34.99	transport.misc									
**245**	F6H0J2	XP_002285876.2	359492398	DPP6 N-terminal domain-like protein (35)	35	35.1	not assigned.no ontology	3,3	2	24,21	**0,020**	0,00970	**0,000**	0,00000	**d.**	**0,069**
**246**	D7T9I6	XP_002278126.1	225423722	Proteasome subunit alpha type-2-B	29	29.5.11.20	protein.degradation.ubiquitin.proteasom	10,6	2	23,98	**0,036**	0,00553	**0,035**	0,00286	**-1,03**	**0,876**
**247**	F6GT83	CBI15230.3	297733983	Glutathione S-transferase	26	26.9	misc.glutathione S transferases	9	2	23,94	**0,092**	0,03579	**0,044**	0,02791	**-2,07**	**0,320**
**248**	F6HBY3	XP_002271902.2	731403637	Nudix hydrolase 3	23	23.3.3	nucleotide metabolism.salvage.NUDIX hydrolases	2,6	2	23,41	**0,019**	0,00651	**0,027**	0,00608	**1,41**	**0,399**
**249**	D7TZC8	XP_002271007.4	1105501277	Uncharacterized protein LOC100247307	35	35.2	not assigned.unknown	13,2	2	23,08	**0,073**	0,02043	**0,028**	0,00803	**-2,61**	**0,069**
**250**	D7T0M7	XP_002266488.1	225445670	Proteasome subunit beta type	29	29.5.11.20	protein.degradation.ubiquitin.proteasom	8,1	2	23,05	**0,010**	0,00483	**0,009**	0,00586	**-1,12**	**0,885**
**251**	D7UE33	CBI40998.3	296090614	PLAT domain-containing protein 3-like	20	20.2	stress.abiotic	10,8	2	22,92	**0,138**	0,01213	**0,076**	0,01540	**-1,81**	**0,011**
**252**	A5AKX5	CAN78023.1	147794812	SOUL heme-binding protein	19	19.99	tetrapyrrole synthesis.unspecified	2,2	2	22,83	**0,104**	0,01706	**0,061**	0,00161	**-1,71**	**0,030**
					29	29.5.11.5	protein.degradation.ubiquitin.ubiquitin protease									
**253**	A5BII5	CAN61395.1	147838865	Pribosyltran domain-containing protein	23	23.3.1.1	nucleotide metabolism.salvage.phosphoribosyltransferases.adenine phosphoribosyltransferase (APRT)	9,2	2	22,43	**0,037**	0,00758	**0,028**	0,00258	**-1,31**	**0,304**
**254**	A5BV56	XP_010652106.1	731395250	elongation factor Tu, mitochondrial	29	29.2.4	protein.synthesis.elongation	9,5	2	22,32	**0,012**	0,00547	**0,012**	0,00692	**-1,01**	**0,987**
**255**	F6I106	XP_002278281.1	225428009	L-ascorbate peroxidase 3, peroxisomal	21	21.2.1	redox.ascorbate and glutathione.ascorbate	6,7	2	21,96	**0,053**	0,00694	**0,064**	0,00639	**1,22**	**0,251**
**256**	F6HCS7	CBI25698.3	297736681	Organellar oligopeptidase A, chloroplastic/mitochondrial	29	29.5	protein.degradation	3,4	2	21,8	**0,001**	0,00079	**0,016**	0,01404	**20,31**	**0,305**
**257**	F6GX19	AEP17008.1	347664495	Isopentenyl-diphosphate Delta-isomerase I	16	16.1.2.7	secondary metabolism.isoprenoids.mevalonate pathway.isopentenyl pyrophosphate:dimethyllallyl pyrophosphate isomerase	10,5	2	21,77	**0,043**	0,00881	**0,006**	0,00626	**-6,85**	**0,007**
**258**	D7SGV3	XP_002283508.1	225456268	Adenylate kinase 4	23	23.4.1	nucleotide metabolism.phosphotransfer and pyrophosphatases.adenylate kinase	13	2	21,7	**0,010**	0,00283	**0,013**	0,00294	**1,34**	**0,432**
**259**	F6HHP3	CBI28440.3	296085025	Glucose-6-phosphate 1-dehydrogenase	7	7.1.1	OPP.oxidative PP.glucose-6-phosphate 1-dehydrogenase (G6PD)	4,4	2	21,55	**0,008**	0,00378	**0,023**	0,00369	**3,01**	**0,017**
					30	30.11	signalling.light									
**260**	D7SIX7	CBI15438.3	297734191	Serine/threonine-protein phosphatase 2A 65 kDa regulatory subunit A beta isoform	29	29.4	protein.postranslational modification	4	2	20,77	**0,000**	0,00000	**0,040**	0,01015	**New**	**0,003**
**261**	F6I5L8	XP_002265881.1	225452186	Prohibitin-3, mitochondrial	9	9.1.1	mitochondrial electron transport / ATP synthesis.NADH-DH (type I).complex I	7	2	20,62	**0,060**	0,00284	**0,050**	0,00457	**-1,19**	**0,103**
					31	31.2	cell.division									
**262**	A0A125R711	YP_009235351.1	1002165223	Ribulose bisphosphate carboxylase large chain	1	1.3.1	PS.calvin cycle.rubisco large subunit	3,7	2	20,51	**0,038**	0,01675	**0,001**	0,00127	**-29,81**	**0,054**
					29	29.2.1.1.1.1.15	protein.synthesis.ribosomal protein.prokaryotic.chloroplast.30S subunit.S15									
**263**	F6GUF3	XP_002285652.2	359478431	Peroxidase 53	26	26.12	misc.peroxidases	6,1	2	20,24	**0,000**	0,00000	**0,061**	0,02693	**New**	**0,048**
**264**	D7TNE5	CBI32018.3	296086429	Hypersensitive-induced response protein 1-like isoform X1	20	20.1.99	stress.biotic.misc	4,5	2	19,66	**0,286**	0,01396	**0,175**	0,03418	**-1,64**	**0,013**
**265**	F6GY71	XP_002272615.1	225439520	Pyruvate decarboxylase 1	5	5.2		4,6	2	19,64	**0,007**	0,00121	**0,000**	0,00037	**-18,60**	**0,000**
**266**	D7TUE8	CBI34123.3	296087534	Glycosyltransferase	26	26.2	misc.UDP glucosyl and glucoronyl transferases	7	2	19,58	**0,118**	0,00517	**0,075**	0,01510	**-1,56**	**0,024**
**267**	A3QRC1	NP_001267900.1	526117952	Allene oxide cyclase 2, chloroplastic (17, 20)	17	17.7.1.4	hormone metabolism.jasmonate.synthesis-degradation.allene oxidase cyclase	9	2	19,26	**0,093**	0,01789	**0,055**	0,00149	**-1,69**	**0,061**
					20	20.2.3	stress.abiotic.drought/salt									
**268**	F6GZK4	XP_002285605.1	225459014	Serine hydroxymethyltransferase	1	1.2.5	PS.photorespiration.serine hydroxymethyltransferase	6	2	18,73	**0,015**	0,00556	**0,012**	0,00691	**-1,24**	**0,740**
					13	13.1.5.2	amino acid metabolism.synthesis.serine-glycine-cysteine group.glycine									
					25	25.1	C1-metabolism.glycine hydroxymethyltransferase									
**269**	F6GUM1	XP_002275617.3	731391766	Ubiquitin-activating enzyme E1 1	29	29.5.11.2	protein.degradation.ubiquitin.E1	3,7	2	17,59	**0,000**	0,00000	**0,005**	0,00271	**New**	**0,093**
**270**	F6HGK8	XP_002283629.2	359484336	3-hydroxyisobutyrate dehydrogenase-like 1, mitochondrial	7	7.1.3	OPP.oxidative PP.6-phosphogluconate dehydrogenase	12,4	2	15,56	**0,011**	0,00647	**0,022**	0,00743	**1,95**	**0,311**
**271**	F6HYJ2	XP_002278700.1	225445861	Transmembrane 9 superfamily member	28	28.99	DNA.unspecified	3,7	2	13,46	**0,023**	0,01102	**0,000**	0,00000	**d.**	**0,061**
